# Exploring the Photophysics and Photocatalytic Activity of Heteroleptic Rh(III) Transition-Metal Complexes Using High-Throughput Experimentation

**DOI:** 10.1021/acs.inorgchem.4c02420

**Published:** 2024-07-20

**Authors:** Stephen DiLuzio, Mitchell Baumer, Rafael Guzman, Husain Kagalwala, Eric Lopato, Savannah Talledo, Joshua Kangas, Stefan Bernhard

**Affiliations:** Department of Chemistry, Carnegie Mellon University, Pittsburgh, Pennsylvania 15213, United States

## Abstract

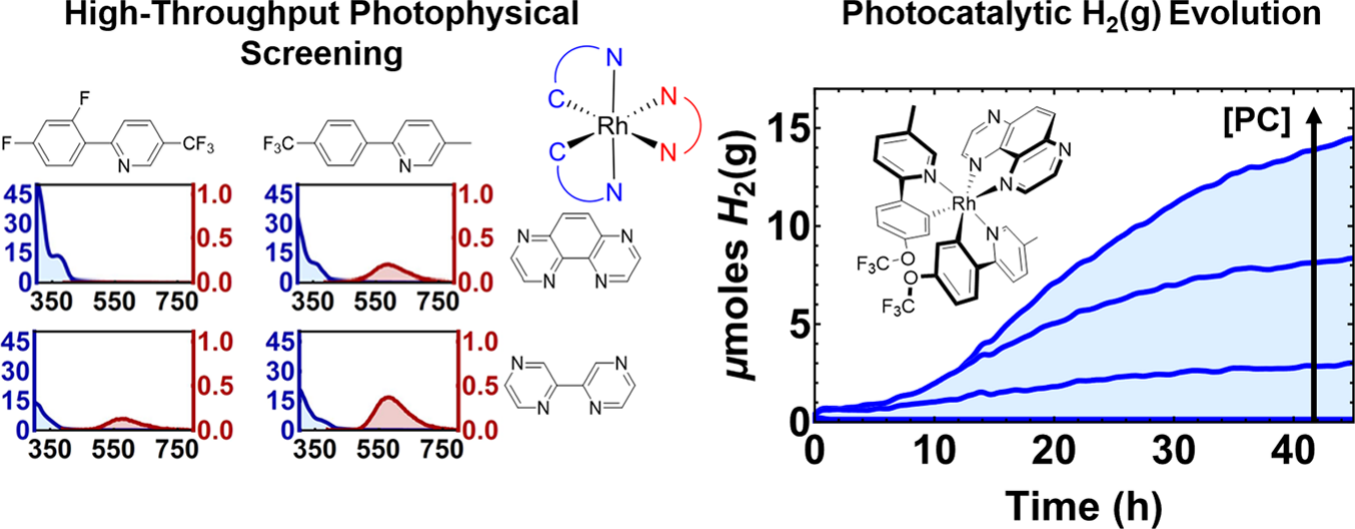

High-throughput synthesis and screening (HTSS) methods were used to investigate the photophysical properties of 576 heteroleptic Rh(III) transition-metal complexes through measurement of the UV–visible absorption spectra, deaerated excited-state lifetime, and phosphorescent emission spectra. While 4d transition-metal photophysics are often highly influenced by deleterious metal-centered deactivation channels, the HTSS of structurally diverse cyclometalating and ancillary ligands attached to the metal center facilitated the discovery of photoactive complexes exhibiting long-lived charge-transfer phosphorescence (0.15–0.95 μs) spanning a substantial portion of the visible region (546–620 nm) at room temperature. Further photophysical and electrochemical investigations were then carried out on select complexes with favorable photophysics to understand the underlying features controlling these superior properties. Heteroleptic Ir(III) complexes with identical ligand morphology were also synthesized to compare these features to this family of well understood chromophores. A number of these Rh(III) complexes contained the requisite properties for photocatalytic activity and were consequently tested as photocatalysts (PCs) in a water reduction system using a Pd water reduction cocatalyst. Under certain conditions, the activity of the Rh(III) PC actually surpassed that of the Ir(III) PC, uncovering the potential of this often-overlooked class of transition metals as both efficient photoactive chromophores and PCs.

## Abstract 2

High-throughput synthesis and screening methods uncovered emissive heteroleptic Rh(III) complexes possessing long-lived, charge-transfer excited states that enabled them to act as photosensitizers in water reduction systems. The desirable photophysical/electrochemical properties were compared to those of Ir(III) congeners to establish clear periodic trends. Spin-orbit interactions largely control the absorption and emission features of the Rh(III) luminophores, while suppressing thermal mixing between the lowest-lying excited-state and metal-centered states is crucial for phosphorescence quantum yields.

## Introduction

Light-to-chemical-energy conversion systems have been and continue to be heavily researched as a potential pathway to a sustainable energy future. Absorption of a photon by a photocatalyst (PC) generates a highly reactive electronic excited state possessing the potent reduction potential needed for light-driven single-electron-transfer (SET) reactions. To overcome diffusion requirements for biomolecular transformations, 4d and 5d transition-metal complexes in combination with strong-field ligand architectures are frequently adopted to both (1) take advantage of the large spin–orbit coupling constant available with heavy-metal centers to access a long-lived triplet excited-state manifold and (2) destabilize metal-centered (MC), antibonding e_g_*-like orbitals that rapidly deactivate the populated excited state.^1−4^ Early photocatalytic systems consequently utilized the archetypical d^6^ [Ru(bpy)_3_]^2+^ PC (bpy = 2,2′-bipyridine), followed by octahedral Ir(III) PCs, whose versatile organometallatic synthetic preparations afford a wide breadth of structural diversity and, in turn, broadly tunable excited-state properties.^5−10^ The resulting advantageous photophysical and electrochemical properties of these transition-metal complexes generally make them robust PCs in a range of photochemical reactions (e.g., solar energy conversion, organic photoredox transformations, CO_2_ reduction, photodynamic therapy, etc.).^11−26^

**Figure 1:**
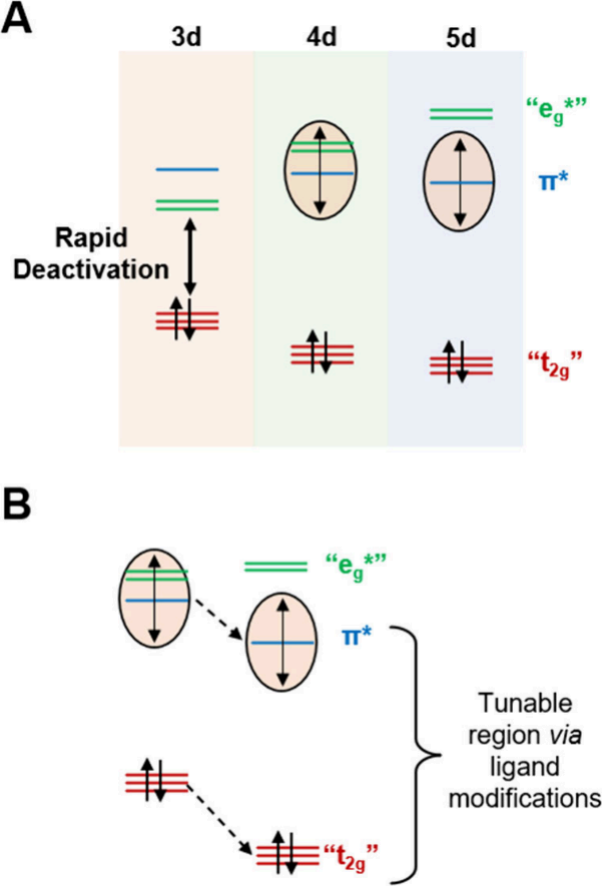
General trends in ligand-field-splitting diagrams observed in first-to-third-row transition-metal complexes. The π* orbitals critical for observing the desirable metal-to-ligand charge-transfer transitions are also added (A). The strong crystal-field splitting of 5d transition metals allows tuning of the LUMO energy levels without the interference of detrimental π*/e_g_* interactions, while 4d central ion ligand-field-based color tuning is commonly limited by such deteriorating effects. Ligand design strategies to tune the emission energy of second-row metal complex excited states that circumvent MC deactivation channels are also depicted (B).

The complicated interplay between the transition-metal center identity and ligand coordination sphere renders the facile generation of versatile PCs challenging (i.e., tunable excited-state redox properties, long-lived, and/or charge-transfer excited state) when moving from 5d transition-metal complexes to those with 3d and some 4d metal centers (Figure 1A). Photoactive 3d PCs are stymied with excited-state deactivation rate constants that inhibit bimolecular reactivity (i.e., >10^10^ s^–1^). Nevertheless, examples of successful design strategies for first- and second-row transition-metal complexes exist: When employing bidentate and tridentate ligands with strongly σ-donating carbanion ligands possessing minimal coordination steric effects, Cr(0), Mn(I), Co(III), Fe(II), and Mo(0) complexes have exhibited both room temperature emission and photocatalytic activity, which resulted from the strongly destabilized MC states;^27−31^ however, recent reports have highlighted more facile methods to generate active PCs.^32,33^ On the other hand, detrimental MC activation has been circumvented through the utilization of d^0^ complexes comprised of Zr(IV) metal centers or Fe(III) metal centers exhibiting favorable room temperature photophysical properties and photocatalytic activity through ligand-to-metal charge-transfer excited states.^34−38^ The ligand molecular structure needed to generate photoactive, first-row transition-metal PCs is often very specific to the transition metal at hand, making comparisons and the modeling of periodic trends challenging when changing the transition-metal identity.

Rh(III) chromophores represent a class of 4d transition-metal complexes that are also largely hindered by MC excited-state dynamics; however, heteroleptic [Rh(C^N)_2_(N^N)]^+^ (where C^N represents a strongly σ-donating cyclometalating ligand and N^N is an ancillary 1,2-diimine ligand) are accessible complexes that mirror the bonding configurations of the widely adopted class of [Ir(C^N)_2_(N^N)]^+^ counterparts that possess highly tunable ground/excited-state properties.^39−45^ This utility arises as a consequence of the spatially separated frontier orbitals that allow independent orbital-energy-level tuning through functional group and ligand morphology variation.^8,39^ In these compounds, the highest occupied molecular orbital (HOMO) resides on the Ir(III) atom and the aryl ring of the organometallic cyclometalating ligand, while the lowest unoccupied molecular orbital (LUMO) is π*-like and localized on the diimine ancillary ligand. Strong mixing between pure metal-ligand-to-ligand charge-transfer (MLLCT) and ligand-centered (LC) excited states results in highly tunable excited-state dynamics dependent on the ligand molecular structure.^8,39^ This unparalleled control over excited-state properties provides the basis for its widespread adoption in diverse light-driven transformations. Extension into Rh(III) systems provides a platform to observe how a diverse C^N/N^N ligand molecular structure can facilitate the generation of chromophores with favorable excited-state properties needed for photocatalysis.

Data-rich methodologies are advantaged approaches to chemical discovery when desirable overall properties are dependent on numerous, interconnected features (e.g., emission excited states and active PCs).^22,24,39,40^ We consequently developed high-throughput synthesis and screening (HTSS) methods to generate a data-rich library on 576 heteroleptic [Rh(C^N)_2_(N^N)]^+^ complexes that not only discovered phosphorescent charge-transfer emission but also exhibit photocatalytic activity in water reduction systems. Our group has developed data-rich methods on [Ir(C^N)_2_(N^N)]^+^ analogues, and we are able to extend it to Rh(III) systems to discover ligand frameworks capable of observing long-lived, charge-transfer phosphorescence at room temperature.^22,24,39,40^ Select [Rh(C^N)_2_(N^N)]^+^ complexes were synthesized and characterized alongside their [Ir(C^N)_2_(N^N)]^+^ counterparts to gain a deeper understanding of the underlying photophysical and electrochemical trends when transitioning from 5d to 4d metal centers. Active chromophores are observed by suppressing mixing of T_1_ and MC states via stabilization of the π* orbitals on the ancillary ligand while also increasing the T_1_/S_0_ energy gap through the introduction of electron-deficient cyclometalating ligands that remove nonradiative deactivation channels. Charge-transfer luminescence for these Rh(III) compounds is rare to observe, and most highly photoactive Rh(III) complexes were previously synthesized with ligands containing extensive π systems.^46−48^ One of the selected complexes exhibited lifetimes suitable for SET reactions as well as sufficient molar absorptivity for visible light (450 nm). HTSS was again used to uncover successful light-driven water reduction systems to form H_2_(g). Although bimetallic Rh(II) species have been used before as molecular PCs for proton reduction in solution, this represents the first report using highly photostable Rh(III) complexes as photosensitizers in water reduction systems.^49−54^

## Results and Discussion

### HTSS Library and Photophysical Screening

**Figure 2:**
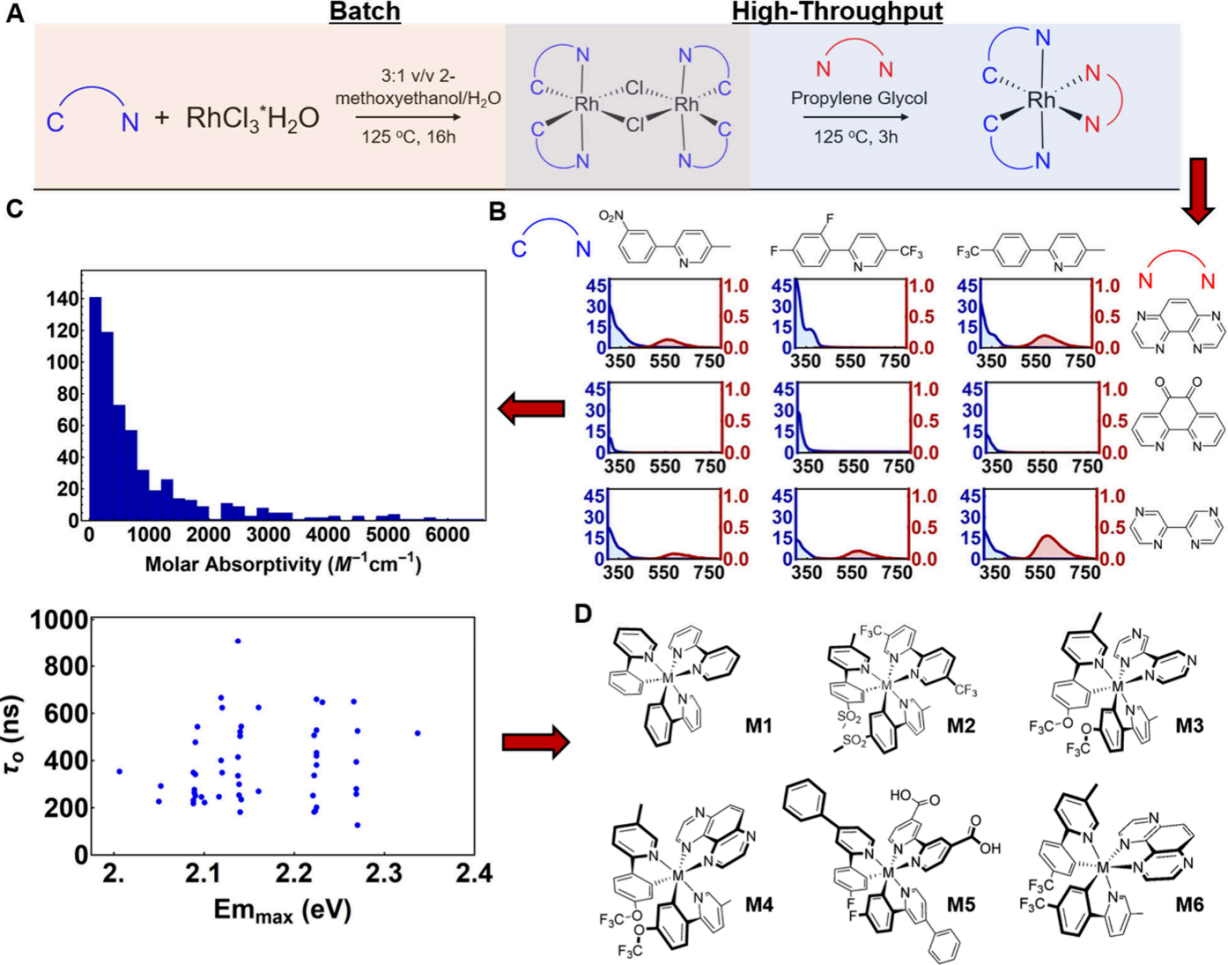
Experimental workflow diagram to elucidate structure activity relationships and discover room temperature emissive Rh(III) complexes through high-throughput experimentation. Batch Rh(III) dimer preparation and high-throughput [Rh(C^N)_2_(N^N)]^+^ synthesis were performed (A). Experimental data collection (UV–visible/steady-state emission spectra) was based on the C^N/N^N ligand identity (B). High-throughput and automated raw data analysis elucidated structure–activity relationships (C). Active ligands capable of generating Rh(III) complexes that are emissive at room temperature [M = Rh(III), Ir(III)] are given (D).

For the parallel synthesis and screening of 576 Rh(III) complexes, a total of 36 cyclometalating ligands (C^N) and 16 ancillary 1,2-diimine ligands (N^N) were used. C^N/N^N ligands tested here included a subset of ligands previously used for a larger library, and all structures are included in Tables S1 and S2.^22,39,40^ Suppressing the thermal population of MC states can be achieved by increasing the energy difference between the highest singly occupied molecular orbital (HSOMO) of the excited state and the e_g_*-like orbitals involved in the ^3^MC state. The spatially separated frontier orbital structure of this class of compounds allows the independent stabilization of the HOMO that is commonly located on the C^N ligand and the Rh(III) central ion. On the other hand, N^N ligands decorated with electron-withdrawing substituents or containing electronegative heteroatoms lower the energy of the ligand’s π* orbital and stabilize the LUMO of the complex (Figure 1B). This approach forces the population of the energetically lower ^3^MLLCT, which also prevents mixing with the photochemically less active ^3^LC excited states.^8,39^ Altogether, the incorporation of electron-deficient C^N/N^N ligands suppresses the rates of MC population while maintaining a suitable T_1_/S_0_ energy gap to slow the rates of nonradiative decay, providing an avenue for charge-transfer emission. The resulting HTSS workflow is depicted in Figure 2: [Rh(Cl)(C^N)_2_]_2_ dimers were synthesized in batch and used in a modified HTS protocol where the Rh dimeric species was heated in propylene glycol to 125 °C in the presence of 2 equiv of ancillary N^N ligand for 3 h (Figure 2A). HTS reaction progress, monitored with ^19^F NMR spectroscopy for a subset of these complexes, indicated successful ancillary ligand binding by the complete emergence of a new signal compared to the original dimeric Rh species (Figures S1–S4).^39^

### HTSS Results

UV–visible results from the HTSS screen indicate that visible-light absorption is quite poor for most Rh(III) complexes, an observation that was documented earlier.^41−45^ Before performing these experiments, we compared the absorption spectra between traditional (see the Experimental Section) and high-throughput methods and found excellent agreement (Figure S5). Example spectra are shown in Figure 2B for nine Rh(III) complexes. Around 73% of the screened [Rh(C^N)_2_(N^N)]^+^ complexes show molar absorptivities between 0 and 1000 M^–1^ cm^–1^ at 450 nm (Figure 2C), a coloration that is unusual when considering most of the previously published [Rh(C^N)_2_(N^N)]^+^ complexes.^41−48^ A smaller percentage of the studied compounds even exhibit molar absorptivity exceeding 1000 M^–1^ cm^–1^, a property that was typically achieved by inserting electron-deficient nitrogen heterocycles with extended conjugation systems (Table S2, **NN56**, **NN57**, and **NN58**). A similar strategy was previously utilized for Ir(III) complexes.^55^ In the investigation of the light emission properties, 62 (∼10%) of the 576 screened compounds exhibit structureless phosphorescence ranging from 620 to 546 nm originating from long-lived excited states with lifetimes ranging between 0.15 and 0.95 μs (Figure 2C). Synthetic tailoring of the frontier orbitals of these complexes therefore achieved a better tunability of the phosphorescence color from red to green compared to that of the aforementioned ruthenium(II) tris(diimine) complexes (e.g., [Ru(bpy)_3_]^2+^), where emission is exclusively observed in shades of orange and red. The broad and structureless emission spectral profiles of the Rh(III) luminophores at room temperature indicate that the emission originates from the desired ^3^MLLCT excited state, which is unique when considering that [Rh(C^N)_2_(N^N)]^+^ or [Rh(N^N)_3_]^3+^ complexes rarely exhibit efficient charge-transfer luminescence at room temperature and their lowest-lying excited state is commonly ^3^LC in character.^41−48,56^ Some compounds did exhibit vibronic substructure, typical for such ^3^LC excited states, which is an indication of the possible presence of mixed excited states, similar to those observed in their Ir(III) cousins.^39^ All spectra are reported in Figures S6–S11. Interestingly, there is no clear correlation between the emission maxima and excited-state lifetime, as one would expect following energy gap law arguments. This is also an indication of a mixed nature (^3^MLLCT/^3^LC) of the excited state and some active participation of MC states in nonradiative decay channels.

It was discovered that the luminescence properties were largely controlled by the ancillary 1,2-diimine ligand identity, where strong emission and long-lived excited-state lifetimes were observed in complexes with electron-deficient ligands such as 1,4,5,8-tetraazaphenanthrene (TAP, **NN55**), 2,2′-bipyrazine (**NN59**), 2,2′-biquinoline (**NN21**), and 5,5′-bis(trifluoromethyl)-2,2′-bipyridine (**NN7**) (Figure 2B,D). Cyclometalating ligands facilitating emission required electron-deficient functional groups linked to the phenyl ring of the cyclometalating ligand (e.g., 5-methyl-2-[(4-trifluoromethyl)phenyl]pyridine, **CN111**), confirming the hypothesis that HOMO stabilization would suppress nonradiative deactivation channels (Figure 2B). Transitioning to an electron-rich C^N/N^N ligand combination did not achieve emissive complexes, likely due rapid MC nonradiative decay or a small T_1_/S_0_ energy gap, forcing tunability capability to exist only when using the electron-deficient ligands. In order to better understand how some of the ligands are capable of supporting emissive and long-lived [Rh(C^N)_2_(N^N)]^+^ excited states, six Rh(III) complexes with a set of judiciously selected ligands that roughly span the λ_max_ and τ_0_ ranges observed in the HTSS were synthesized and isolated traditionally (Figure 2D). The corresponding Ir(III) analogues were also prepared for feature comparison, and Ir(III) and Rh(III) parent complexes were assembled with 2-phenylpyridine as the C^N ligand and 2,2′-bipyridine as the ancillary ligand (**Rh1**/**Ir1**) to provide a baseline for the study. All traditionally synthesized complexes were fully characterized, and all analytical data are reported in the Supporting Information.

#### Photophysics

**Figure 3:**
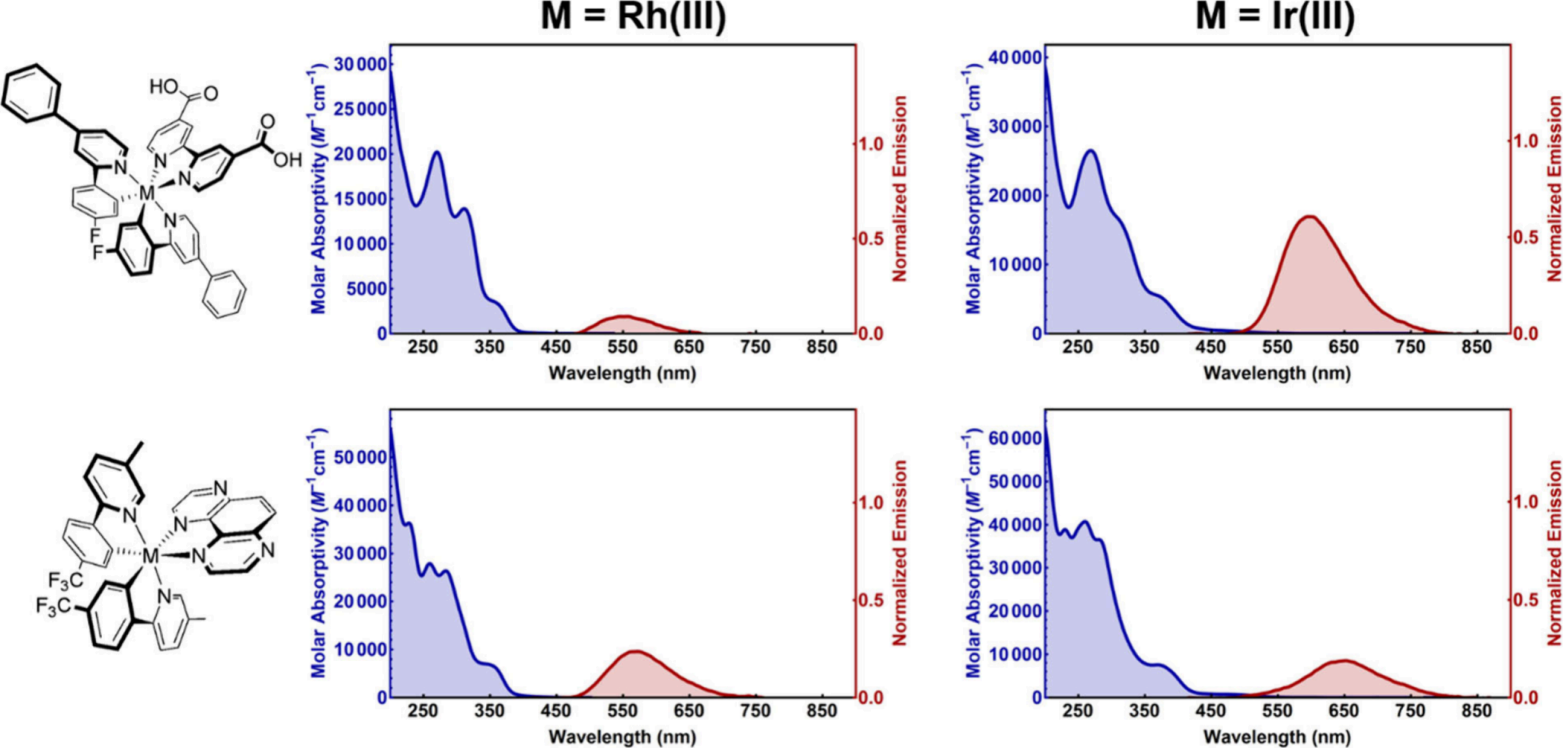
UV–visible absorption and emission spectra for two Rh(III) and Ir(III) transition-metal complexes (**M5** and **M6**). Absorption spectra were measured in aerated ACN, while emission spectra were collected in deaerated ACN. The structureless spectral profile indicates charge-transfer emission in both complexes. Emission spectra are normalized relative to the 12 complex sets to allow the comparison of the emission intensities. Absorption spectra are bathochromically shifted when Rh(III) is replaced with Ir(III), a result of the less prominent direct excitation into low-lying triplet states.

The absorption spectra, emission spectra, and excited-state lifetime were measured for all six Rh(III)/Ir(III) complexes studied here (Table 1 and Figures S12–S14). Comparisons of the absorption spectra of Rh(III)/Ir(III) complexes with an identical set of C^N/N^N ligands confirmed that the absorption of Rh(III) is hypsochromically shifted in all cases, as expected from the HTSS investigation. There are, however, notable differences between the Ir(III) and Rh(III) data that highlight the influence of spin–orbit coupling on the absorption tails that extend into the visible region. Although the lack of observed vibronic structure in this region makes assigning transitions not straightforward, the lowest-energy transitions are often assigned to direct S_0_ → T_*x*_ excitations, which allow the direct population of ^3^MLLCT or ^3^LC excited states.^57^ The data presented here show less prominent mixing between singlet and triplet states as a result of the lower spin–orbit coupling constant of Rh(III), which will diminish the probability of spin-forbidden processes, rendering the triplet excited states less accessible through direct light absorption (Figure 3).^57^

**Table 1:** Photophysical Properties of Heteroleptic [Rh(C^N)_2_(N^N)]PF_6_ and [Ir(C^N)_2_(N^N)]PF_6_ Studied Here

	ε^a^	λ_max_(nm)	τ_0_(μs)^b^	QY^c^ (%)	*k*_nr_(×10^6^_,_s^–1^)	*k*_r_(×10^6^_,_s^–1^)		ε^a^	λ_max_(nm)	τ_o_(μs)^b^	QY^c^ (%)	*k*_nr_(×10^6^_,_s^–1^)	*k*_r_(×10^6^_,_s^–1^)
**Rh1**	0	544		0.08			**Ir1**	520	588	0.37	6.04	2.54	0.16
**Rh2**	0	559	0.14	0.37	7.12	0.03	**Ir2**	200	561	0.62	1.26	1.59	0.02
**Rh3**	0	559	0.62	0.85	1.60	0.01	**Ir3**	960	653	0.14	0.5	7.11	0.04
**Rh4**	360	574	0.73	0.72	1.36	0.01	**Ir4**	880	653	0.19	0.75	5.22	0.04
**Rh5**	0	556	0.41	0.59	2.42	0.01	**Ir5**	520	596	0.49	3.98	1.96	0.08
**Rh6**	40	571	0.75	1.54	1.31	0.02	**Ir6**	800	653	0.18	1.23	5.49	0.07

a Measured value for 450 nm: units are M^–1^ cm^–1^.

b Measured in deaerated CH_3_CN (argon).

c Measured using [Ru(bpy)_3_]^2+^·2PF_6_ as a standard.

Steady-state emission spectra indicate that the emission maxima of the ^3^MLLCT excited state were, on average, 0.20 ± 0.11 eV higher in energy for the Rh(III) complex compared to the Ir(III) analogue (Table 1). Most notably, the ligand selection used in this study resulted in structureless emission spectral profiles, which are the hallmark of charge-transfer excited states. In contrast, emission with a vibronic substructure typically arises from π* → π phosphorescent deactivation pathways. In Ir(III) complexes, this is commonly observable in ligand environments with extended π systems or at low temperatures with central Ir(III) or Rh(III) central ions. It was remarkable that the HTSS protocols uncovered an abundance of ^3^MLLCT excited-state phosphorescence in Rh(III) photosensitizers, where efficient phosphorescence at room temperature typically occurs from a ^3^LC state.^46−48^ Analogous trends are observed with Ru(II) and Os(II) chromophores with similar ligand molecular structure (i.e., a bathochromic shift in the UV–visible spectra due to more facile S_0_ → T_*n*_ transitions for Os(II), while higher energy emission is observed for the Ru(II) luminophores).^58−61^

The room temperature excited states for these complexes were long-lived, with lifetimes ranging from ∼0.14 to 0.75 μs, which is similar or in some cases even longer compared to the equivalent Ir(III) complexes (Table 1). The phosphorescence quantum yields range between 0.37 and 1.5%, which are low compared to luminescent Ir(III) complexes but compare well with the previously published heteroleptic Rh(III) complexes.^45^ Interestingly, the lower quantum yields for these complexes do not seem to occur from rapid rates of nonradiative decay from the triplet state because these rates are comparable to those of the Ir(III) congeners (Table 1). Instead, it is likely that the low spin–orbit coupling of Rh(III) hinders mixing between the excited triplet state and singlet states, therefore lowering the rate of intersystem crossing (*k*_isc_) and forcing deactivation via internal conversion pathways from the singlet manifold.^46^ The photophysical features compare well with the identical features measured from HTSS (Table S3), confirming the validity of the HTSS method, where minor differences are attributed to solvatochromic effects.

#### Electrochemistry

**Figure 4:**
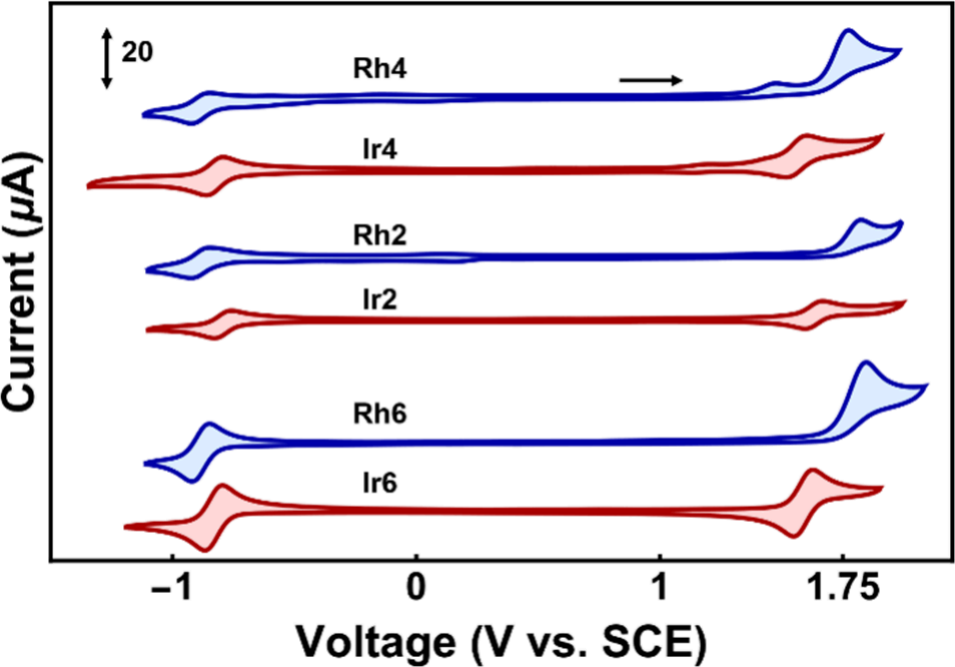
Cyclic voltammograms of 6 representative Rh(III) and Ir(III) PCs selected from the group of 12 traditionally synthesized complexes. Scans were collected with 0.1 M tetrabutylammonium hexafluorophosphate supporting electrolyte solutions (in ACN) containing 2.0 mM analyte at a 0.1 V s^–1^ scan rate and referenced to the SCE using an internal ferrocene standard.

The impact that changing of the metal center has on frontier orbital energy levels was evaluated using cyclic voltammetry on all 12 complexes. Negative scans resulted in quasi-reversible cathodic peaks (PC^+^/PC^0^) assigned to a ligand reduction process and ranging from −0.88 to −1.45 V for the Rh(III) complexes and from −0.79 to −1.4 for the Ir(III) complexes (Table 2, vs saturated calomel electrode (SCE)). On the other hand, metal- and C^N-centered anodic peaks (PC^2+^/PC^+^) range from +1.44 to +1.77 V and from +1.32 to +1.66 V for Rh(III) and Ir(III), respectively (Table 2, vs SCE). Comparing the data between the two transition metals highlights two distinct differences: (1) The oxidation of the Rh(III) complexes proceeds irreversibly (Figure 4) compared to the Ir(III) complexes, and (2) the electrochemically measured potential gaps between oxidation and reduction are wider for the Rh(III) complexes compared to the Ir(III) coordinated to identical ligands (Table 2). The two observations are confirming previously reported trends.^42,43^ Both HOMO stabilization and LUMO destabilization are responsible because the (PC^2+^/PC^+^) redox couples are, on average, shifted toward more positive potentials by 137 mV (±19 mV), while the (PC^+^/PC^0^) redox couples are, on average, shifted to more negative potentials; an average of −75 mV (±19 mV) is observed for the Rh(III) complexes. Similar electrochemical observations are again observed between Ru(II) and Os(II) transition metals, suggesting that periodic trends exist when moving from 5d to 4d complexes.^61^

**Table 2:** Tabulated Ground-State Reduction Potentials vs SCE for the Heteroleptic [Rh(C^N)_2_(N^N)]PF_6_ and [Ir(C^N)_2_(N^N)]PF_6_ Transition-Metal Complexes Studied Here

.	*E*_red_(PC^+^/PC^0^)	*E*_ox_(PC^2+^/PC^+^)^a^	PC*^+^/PC^0^	PC^–^/PC*		*E*_red_(PC^+^/PC^0^)	*E*_1/2_(PC^2+^/PC^+^)	PC*^+^/PC^0^	PC^–^/PC*
**Rh1**	–1.45(89)	+1.44	N/A	N/A	**Ir1**	–1.4(71)	+1.32(95)	+0.71	–0.78
**Rh2**	–0.89(79)	+1.76	+1.33	–0.46	**Ir2**	–0.79(64)	+1.63(70)	+1.42	–0.58
**Rh3**	–0.88(76)	+1.74	+1.34	–0.48	**Ir3**	–0.79(69)	+1.58(87)	+1.11	–0.32
**Rh4**	–0.91(70)	+1.70	+1.25	–0.46	**Ir4**	–0.83(63)	+1.56(87)	+1.07	–0.34
**Rh5**	–0.88(12)	+1.77	+1.35	–0.46	**Ir5**	–0.8(81)	+1.66(87)	+1.28	–0.42
**Rh6**	–0.88(72)	+1.75	+1.29	–0.42	**Ir6**	–0.83(74)	+1.59(81)	+1.07	–0.31

a Irreversible peaks were assigned using the inflection point of the anodic scan.

These electrochemical data are generally in good agreement with the observed higher energy phosphorescence peaks of the Rh(III) complexes compared to the Ir(III) complexes as well as the influence of spin-orbital coupling needed for direct S_0_ → T_*n*_ excitation. The optical gap defined by the absorption onset (ε = 150 M^–1^ cm^–1^) is smaller than the electrochemical gap for the Ir(III) complexes. On the other hand, the optical gap for the Rh(III) complexes using the same criterion is larger than the electrochemical gap (Figure S15). Estimating the excited-state reduction potentials at room temperature suggests that the Rh(III) excited states are potent photooxidants, albeit weak photoreductants (Table 2). Because heteroleptic complexes are often used as strong photooxidants, substitution of Ir(III) metal centers for Rh(III) offers the ability to extend the available redox window needed for reductive quenching pathways.

#### Density Functional Theory (DFT) Calculations

**Figure 5:**
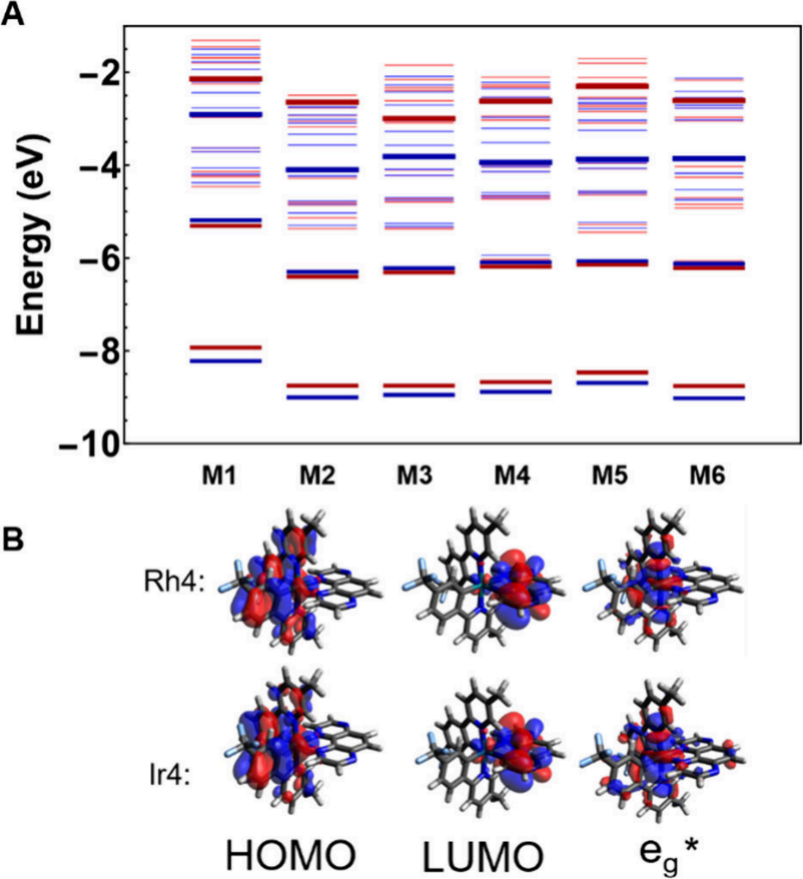
Molecular orbital diagrams for all 12 Rh(III) (blue) and Ir(III) (red) complexes studied in this work (A). Frontier orbitals and virtual metal-centered states are in boldface to highlight their energetic position. Orbital representations of the HOMO/LUMO showing a similar electronic distribution in the frontier orbital structure for **Rh4**/**Ir4** (B). Antibonding orbitals centered on the metal (e_g_*) are also shown.

Ground-state singlet electronic structure calculations of the frontier orbitals were performed for all 12 complexes to provide a more descriptive picture of the excited-state and electrochemical properties. Results agree with the electrochemical data: HOMO energy levels are stabilized for the Rh(III) complexes, while LUMO energy levels are destabilized compared to the Ir(III) derivatives with the same ligand structure (Figure 5A). All complexes exhibit HOMO electronic density centered on the Ir(III)/Rh(III) d orbitals and the phenyl ring of the cyclometalating ligand, while the LUMO are π* in character located predominantly on the ancillary ligand (Figures 5B and S16 and S17). Bond-length analysis indicates that the calculated Rh–C_(C^N)_ bonds are always shorter than the Ir–C_(C^N)_ bonds (Table S4). This could indicate stronger cyclometalated bonding and, in conjunction with the higher electronegativity of Rh, would explain the more positive *E*_ox_(PC^2+^/PC^+^) oxidation potentials. Given this stronger bonding and the prominence of the Rh(III)–C bond in HOMO, the irreversible oxidation could be a reflection of these findings (Figure 4). Interestingly, the Rh(III)–N_(C^N)_ and Rh(III)–N_(N^N)_ bond lengths are longer than the Ir(III)–N_(C^N)_ and Ir(III)–N_(N^N)_ bond lengths. This could rationalize the more negative *E*_1/2_(PC^+^/PC^0^) reduction potentials observed for Rh(III): Weaker binding interactions of the ancillary ligand result in an increased π*-orbital energy located more exclusively on the N^N ligand and, consequently, a LUMO that is more challenging to reduce (Figure 5A,B).

The longer Rh–N bond lengths on both C^N and N^N ligands, in conjugation with the lower crystal-field splitting of Rh, result in lower-lying dissociative virtual orbitals on the metal (e_g_*-like) for all Rh(III) complexes (Figure 5A,B). While the excited-state lifetimes of the Rh(III) complexes are generally longer than those of the Ir(III) complexes, the quantum yields are much smaller (Table 1). Nonradiative decay is a function of both the energy gap between T_1_ and S_0_ and the rates of thermal population of the rapidly deactivating MC state. Deciphering which contribution is more impactful to the overall rate of nonradiative decay is challenging. The energy gap between the LUMO and e_g_*-like state is notably smaller for the Rh(III) chromophores compared to the Ir(III) complex with the same molecular structure (Figure 5A). **Rh1** ([Rh(ppy)_2_(bpy)]^+^) is the highest energy emitter in this set yet possesses the lowest quantum yield (Table 1). The destabilized LUMO (Table 2) observed with bpy likely renders thermally populated MC states the dominant contributor to *k*_nr_. It is therefore possible, despite *k*_nr_ for the Rh(III) complexes being similar to those of the Ir(III) complexes (Table 1), that MC deactivation is a contributing term for **Rh2**–**Rh6** as well.

#### Photocatalytic Hydrogen Evolution from Water

**Figure 6:**
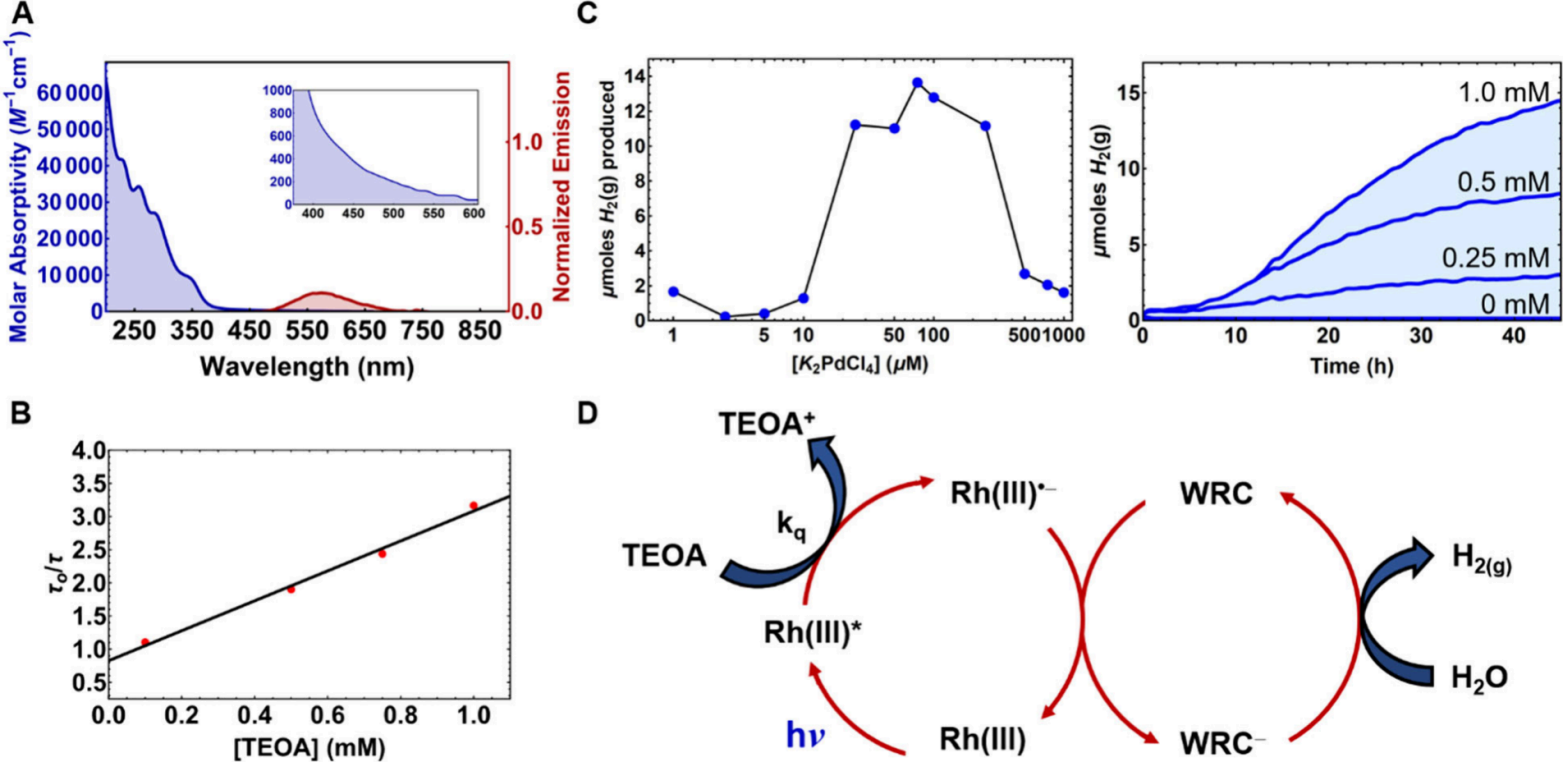
UV–visible absorption and emission spectra for **Rh4**. Inset: Absorption spectral tail extending into the visible region (A). Stern–Volmer plot depicting interactions between Rh4* and TEOA (B). Total amount of H_2_(g) produced as a function of the K_2_PdCl_4_ precatalyst concentration ([**Rh4**] = 1.0 mM; [TEOA] = 0.18 M) and H_2_(g) traces dependent on [**Rh4**] ranging from 0 to 1.0 mM ([K_2_PdCl_4_] = 0.025 mM; [TEOA] = 0.18 M) (C). Proposed dual catalytic cycle using **Rh4** as a PC with the associated *in situ* formed colloidal WRCs (D).

The long-lived excited states observed by the photophysical analysis, favorable electrochemical parameters of some of these Rh(III) complexes, and potent excited-state reduction potentials (Table 2) made them promising candidates for use as PCs in water reduction systems operating through reductive quenching pathways. In particular, **Rh4** possesses the required features for photocatalytic activity: the excited-state lifetime of 0.73 μs is sufficiently long for diffusion-based SET reactions to proceed, and light absorption is facilitated by an absorption tail extending into the visible region, a prerequisite for any solar energy conversion applications [Figure 6A (inset) and Table 1]. Further, the charge-transfer nature of this excited state allows for significant structural changes in the excited molecules, which disfavors unproductive back-electron-transfer/charge recombination effects.^40^ Given the lack of stability of Rh(III) upon oxidation (Figure 4), we opted for a photocatalytic reaction that involves a reductive quenching cycle in a standard water reduction system using triethanolamine (TEOA) as a sacrificial electron donor and K_2_PdCl_4_ as a Pd precursor for the *in situ* formation of a colloidal water reduction catalyst (WRC).^62,63^ Stern–Volmer analysis using the luminescence lifetime (τ) confirms that the TEOA reductively quenches the excited-state phosphorescence of **Rh4** with a near-diffusion-limited quenching rate constant (*k*_q_ = 2.38 × 10^9^ M^–1^ s^–1^; Figure 6B) in acetonitrile (ACN), confirming the strong oxidative power of these complexes (Table 2). Next, we mixed a solution of **Rh4** and TEOA, irradiated the deaerated solution with blue light, and observed a rapid transformation in the color of the sample, attributed to the formation of a highly absorbent radical species, most likely PC^0^ (Table S5). UV–visible spectra of these samples indicated that this new species strongly absorbs in the visible region, which indicates that **Rh4** allows high enough Cage Escape efficiencies suitable for photocatalysis (Figure S18).^40^ This radical species is also highly stable because the color saturation of these solutions is persistent (Table S5). Rapid reaction optimization was then carried out using a home-built high-throughput photoreactor capable of monitoring hydrogen evolution in parallel with hydrogen-sensitive tape.^59^ The study pinpointed conditions in which H_2_(g) evolved efficiently using dimethyl sulfoxide (DMSO) as a solvent in the presence of excess water (Figure 6C). The concentration of K_2_PdCl_4_ strongly influenced the rate of hydrogen production, where the highest performing concentrations ranged between 25 and 100 μM (Figure 6C). Optimum reaction conditions yield PC turnover numbers of 34 for **Rh4** for the proposed dual catalytic cycle (Figure 6D), which is comparable to similar, unoptimized Ir(III) systems.^11^ Control experiments indicate that TEOA, K_2_PdCl_4_, and **Rh4** were all necessary for H_2_(g) production (Table S6).

**Rh1** and **Rh6** also exhibit moderate light absorption at the irradiation wavelength (Table 1), and the two PCs were tested for their ability to photogenerate hydrogen. Although **Rh1** was observed to be inactive across all tested conditions, the formation of H_2_(g) was observed when using **Rh6** as a PC; however, lower photocatalytic activity was observed compared to **Rh4**, which can be readily rationalized by the weaker absorbance of the **Rh6** solutions at equal concentrations of PC (Figure S19). When the hydrogen evolution activities between **Rh4** and **Ir4** were compared, it became clear that **Ir4** produces more H_2_(g) at equal concentrations of PC, also following the trend in molar absorptivity (Table 1 and Figure S20). The total amount of H_2_(g) evolved for absorbance-matched samples ([**Ir4**] = 0.41[**Rh4**]) was larger when using **Rh4** as the PC [9.9 ± 1.2 μmol of H_2_(g)] compared to **Ir4** [4.6 ± 1.1 μmol of H_2_(g)]. This could indicate that the **Rh4** complex is a stronger photooxidant, better at suppressing rates of charge recombination, or overall a more robust catalyst.

## Conclusions

HTSS protocols were used to rapidly screen the photophysical properties (UV–visible absorption and emission spectra and time-resolved excited-state deactivation) of 576 heteroleptic [Rh(C^N)_2_(N^N)]^+^ complexes with a diverse ligand structure. Electron-poor ancillary ligands were selected to stabilize the LUMO in an effort to suppress thermal population of nonradiative MC and photochemically less active LC states. Cyclometalating ligands include electron-deficient functional groups to stabilize the HOMO and suppress energy-gap-law-associated nonradiative deactivation channels by blue-shifting the optical gap. Automated data analysis selected the top C^N/N^N ligand combinations for generating complexes exhibiting room temperature phosphorescence originating from charge-transfer excited states possessing long-lived lifetimes. These findings represent the first report of substantial color tunability for this class of Rh(III) compounds (red to green emission color) and document the success of the HTSS methods. Further electrochemical and theoretical analysis offered insights into the electronic structure of 12 traditionally synthesized Rh(III) and Ir(III) complexes. The effect of spin-orbital coupling differences between the Rh(III) and Ir(III) central ions was highlighted when both the light absorption tail into the visible region and the rates of radiative decay (and the resultant quantum yields) was compared. The ancillary ligand clearly controls the likelihood of generating of a photoactive transition-metal complex, suggesting that exploration of this chemical space could uncover other Rh(III) chromophores. Ancillary ligands with O^O and N^O coordination environments have created emissive Ir(III) species. These anionic bidentate ligands could destabilize MC states and, in turn, generate emissive Rh(III) complexes, and, consequently, will be subject to future investigation from our group.^64−67^ Finally, these screening techniques identified a Rh(III) complex possessing the required characteristics for PC activity, and this work represents the first report of a d^6^ Rh(III) molecular PC used to drive photocatalytic water reduction systems.

## Experimental Section

Reagents and solvents were purchased from various commercial [Sigma-Aldrich, Tokyo Chemical Industry (TCI), Acros Organics, J. T. Baker, Beantown Chemical, and Matrix Scientific] sources and used without further purification. NMR spectra were obtained using a Bruker Avance 500 MHz; ^1^H NMR spectra were referenced to residual solvent peaks.

### Synthesis of Cyclometalating Ligands

Synthesis of all C^N ligands was carried out using standard literature procedures.^39,40^

### Synthesis of Precursor [RhCl(C^N)_2_]_2_ and [IrCl(C^N)_2_]_2_ Dimers

Precursor dimer compounds for both the high-throughput screening and traditional synthesis were prepared by reacting the corresponding C^N ligand in 2.1:1 mol equiv with respect to RhCl_3_·*x*H_2_O or IrCl_3_·*x*H_2_O in a 3:1 (v/v) 2-ethoxyethanol/water solution at 125 °C for 16 h. Upon reaction, a colored precipitate precipitated from the solution. Generally, the rhodium dimers were much less colored than their Ir(III) analogues. Upon cooling, the product precipitation was completed by the addition of deionized water and isolated using vacuum filtration. The product was washed with diethyl ether, collected in good yields, and used without further characterization.

### Traditional Synthesis of [Rh(C^N)_2_(N^N)]PF_6_ and [Ir(C^N)_2_(N^N)]PF_6_ Complexes

[RhCl(C^N)_2_]_2_ and [IrCl(C^N)_2_]_2_ dimers and the corresponding N^N ligand at a 1:2.1 molar ratio were added to propylene glycol, and the mixtures were heated to 150 °C for 16 h. After cooling, a saturated solution of aqueous K^+^PF_6_^–^ was added, and the resulting complex crashed out. The solution was allowed to stir for 30 min for anion exchange and isolated through vacuum filtration. The product was washed with diethyl ether and collected. The raw product was purified via ether/ACN vapor crystallization.

### HTSS

Stock solutions of 0.5 mM [RhCl(C^N)_2_]_2_ dimer and 1 mM N^N ligand in acetone were joined in a 1 mL well, and the solvent was allowed to evaporate overnight. For emission screening, 250 μL of each stock solution was used, and subsequently 500 μL of propylene glycol was added, resulting in a final concentration of 0.50 mM. For absorption spectral screening, 10 μL of each acetone stock solution was used and subsequently 200 μL of propylene glycol was added, resulting in a final concentration of 50 μM in propylene glycol. The solutions were mixed and heated to 125 °C for 3 h in a 96-well plate.

### High-Throughput Synthetic Yield Assessment

The reaction efficiency was assessed by using ^19^F NMR spectroscopy.

### High-Throughput UV–Visible Absorption Spectroscopy

Absorption spectra were measured with a ThermoFisher Scientific microplate reader.

### UV–Visible Absorption Spectroscopy

UV–visible absorption spectra were collected with a Shimadzu UV-1800 spectrophotometer in 25.0 μM aerated ACN solutions.

### Quantum Yield Measurements

Phosphorescence quantum yields were calculated relative to those of [Ru(bpy)_3_]^2+^·2PF_6_^–^ using the following equation:
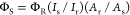
1where Φ_S_ is the quantum yield of the sample and Φ_R_ is the quantum yield of the reference compounds (Φ_R_ = 6.2% in ACN at room temperature). *A*_r_ and *A*_s_ represent the absorption of the reference and sample, respectively, at the excitation wavelength, and *I*_s_ and *I*_r_ are the corresponding integrated emission spectral integrals calculated after spectral correction for the sample and reference, respectively. Samples were degassed by bubbling argon for 15 min.

### Calculation of the Radiative and Nonradiative Decay Constants

The radiative and nonradiative decay constants of the excited state were calculated using the following equations:
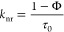
2
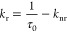
3where Φ is the phosphorescent quantum yield and τ_0_ is the deaerated phosphorescence lifetime in seconds.

### Calculation of the Excited-State Reduction Potential

The excited-state oxidation and reductive potentials were calculated using the following equations:^68^
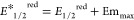
4
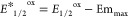
5where E*_1/2_^red^ and E*_1/2_^ox^ are the excited-state reduction and oxidation potentials, while *E*_1/2_^red^ and *E*_1/2_^ox^ are the ground-state M^I/0^ and M^II/I^ redox couples, respectively. The excited-state redox potentials were approximated using Em_max_ (the emission maxima in electronvolts) because no vibronic substructure was observed at room temperature.

### Luminescence Measurements

Both the high-throughput-synthesized Rh(III) complexes and the traditionally synthesized [Ir(C^N)_2_(N^N)]PF_6_/[Rh(C^N)_2_(N^N)]PF_6_ were irradiated with a pulsed 365 nm light-emitting diode (LED), powered by a 40 ns square pulse from a Siglent SDG1052 function generator. High-throughput samples were purged with argon for 2 h in a home-built chamber, while the traditionally synthesized samples were purged with argon for 5 min. Emission was detected with a Hamamatsu H7732-11 photomultiplier tube connected to a Tektronix TDS3032B digital oscilloscope interfaced to a Raspberry Pi 3 Model B+ computer. Scattered light from the excitation source was removed from the acquired spectra by using a plexiglass filter (365 nm long pass). Emission spectra were measured concurrently by using a StellarNet BLACK-Comet concave grating spectrometer and corrected for spectrometer sensitivity.

### Electrochemistry

Cyclic voltammetry (CV) was performed using a CH-Instrument Electrochemical Analyzer 600C potentiostat with a three-electrode system consisting of a silver wire pseudoreference electrode, a platinum coil counter electrode, and a glassy carbon working electrode. Experiments were performed at 2 mM analyte concentrations with a 0.1 M tetra-*n*-butylammonium hexafluorophosphate supporting electrolyte in ACN after purging the solution with argon until no traces of oxygen signals were visible in the CV plot. Voltammograms were collected with a scan rate of 0.1 V s^–1^, and redox potentials were referenced relative to a ferrocene internal standard [*E*^0^(Fc^+^/Fc) = 0.40 V vs SCE in ACN].

### DFT Calculations

The electronic structure was modeled with singlet- and triplet-based DFT through geometry optimization at the B3LYP level of theory using the LANL2DZ basis set in *Gaussian 16*. *LANL2DZ* was used for all atoms because it provides more accurate results compared to mixed basis sets.^69^

### Hydrogen Evolution Experiments

Parallelized hydrogen evolution experiments were performed in a home-built photoreactor.^63^ A 108-well plate was used to generate H_2_(g) evolution traces dependent on the concentration of PC, sacrificial electron donor (TEOA), and K_2_PdCl_4_ precatalyst in 1 mL shell vials. The total volume of each sample was kept constant at 440 μL in aerated 10:1 (v/v) DMSO/H_2_O solutions. Samples were irradiated with 445 nm light using two 100 W LEDs. Quantification of H_2_(g) was monitored online through a colorimetric detection system using DetecTape, a chemoselective tape sensitive to H_2_(g). Calibration of this system was used as described previously.^63^

## Supporting Information Available

All supporting figures and tables (PDF)
Extracted features from Gaussian fitting (XLSX)
