# Supplementary material for: Exploring the Photophysics and Photocatalytic Activity of Heteroleptic Rh(III) Transition-Metal Complexes Using High-Throughput Experimentation
Source: Inorg Chem. 2024 Jul 20;63(31):14267–77. doi: 10.1021/acs.inorgchem.4c02420 (PMC11304382; doi:10.1021/acs.inorgchem.4c02420)
Supplement: Supplementary file 1 — ic4c02420_si_001.pdf [file ic4c02420_si_001.pdf]

Supporting Information

Exploring the Photophysics and Photocatalytic Activity of Heteroleptic Rh(III) Transition Metal Complexes using High-Throughput Experimentation

Stephen DiLuzio, Mitchell Baumer, Rafael Guzman, Husain Kagalwala, Eric Lopato, Savannah Talledo, Joshua Kangas, Stefan Bernhard*

*Corresponding Author: bern@cmu.edu

Department of Chemistry, Carnegie Mellon University, Pittsburgh, Pennsylvania 15213, USA

Table of Contents

Table of all cyclometalating (C^N) ligands used to form heteroleptic $[Rh(C^\wedge N)_2(N^\wedge N)]^+$ complexes. Structures and corresponding "C^N" identification coding are shown …..…….S4-S5

Table of all ancillary (N^N) ligands used to form heteroleptic $[Rh(C^\wedge N)_2(N^\wedge N)]^+$ complexes. Structures and corresponding "N^N" identification coding are shown……………..…..……….S6

^{19}F NMR spectra of heteroleptic Rh(III) complexes containing **CN111** with various diimine ancillary ligands……………………………………………………………..…………..…S7

^{19}F NMR spectra of heteroleptic Rh(III) complexes containing **CN14** with various diimine ancillary ligands for in aryl-CF_3 chemical shifts……………………………..……………......S8

^{19}F NMR spectra of heteroleptic Rh(III) complexes containing **CN14** with various diimine ancillary ligands for the aryl-F chemical shifts………………………..…………...……S9

^{19}F NMR spectra of heteroleptic Rh(III) complexes containing **CN11** with various diimine ancillary ligands…………………………………………………………………………...…S10

Overlayed UV-visible spectra measured using traditional and high-throughput methods for 22 heteroleptic Ir(III) previously measured ……………………………………….....…S11-S13

High throughput screened absorption and deaerated emission spectra in propylene glycol for all ligands screened………………………………………………………………...S14-S16

UV-vis absorption and emission spectra of **Rh2 – Rh6**……………………….........………S17

UV-vis absorption and emission spectra of **Ir2 – Ir6**…………………………………….…S18

UV-vis absorption and emission spectra of **Rh1 and Ir1**……………………........………S19

Table comparing photophysical features from HTSS and traditional approach……………….S20

Dependence of the optical gap on the electrochemical gap for the 6 Rh(III) and Ir(III) studied here……………………………………………………………………………………..S21

Frontier orbital diagrams for **Rh1-Rh6**……………………………………..…..………..S22

Frontier orbital diagrams for **Ir1-Ir6**……………………………………..…………..…..S23

Tabulated Rh-$N_{C^\wedge N}$, Rh-$C_{C^\wedge N}$, Rh-$N_{N^\wedge N}$, Ir-$N_{C^\wedge N}$, Ir-$C_{C^\wedge N}$, and Ir-$N_{N^\wedge N}$ bond lengths computed from density functional theory calculations……………………………………………………S24

Tabulated pictures showing the change in solution color upon illumination of **Rh4** with blue light in the presence of Triethanolamine as well as its radical stability………………...……...…S25

Overlayed UV-visible absorption spectra of **Rh4** and the radical intermediate of **Rh4** formed after reduction quenching…………………………………………………………………S26

Tabulated results from control experiments for water reduction systems……………………...S27

Hydrogen evolution traces for **Rh4** and **Rh6**……………………………...………...…………S28

Hydrogen evolution traces for **Rh4** and **Ir4**………………………………...…….…………S29

Characterization of Rh(III)/Ir(III) Complexes……………………………………….……S30-S59

References…………………..……………………………………………………………...S60

Table S1: All cyclometalating (C^N) ligands used to form heteroleptic $[Rh(C^\wedge N)_2(N^\wedge N)]^+$ complexes. Structures and corresponding "**C^N**" identification coding are shown.

**CN1**	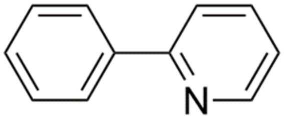	**CN67**	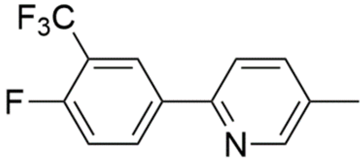
**CN2**	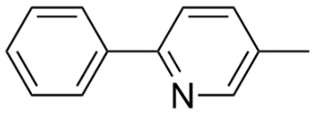	**CN70**	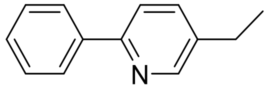
**CN5**	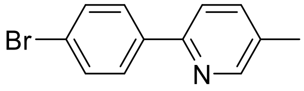	**CN74**	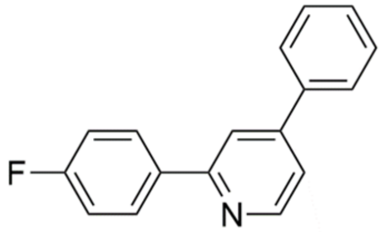
**CN7**	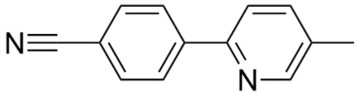	**CN95**	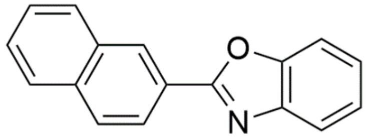
**CN8**	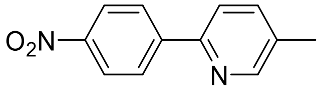	**CN101**	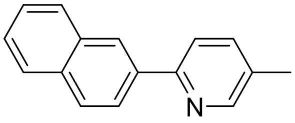
**CN9**	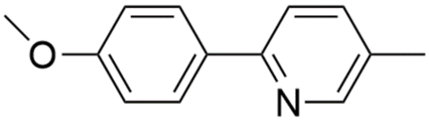	**CN104**	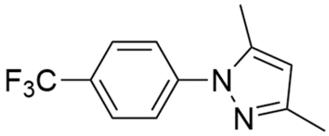
**CN11**	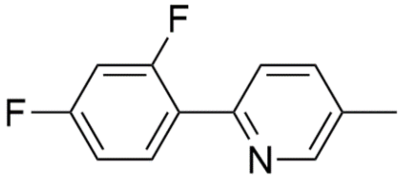	**CN105**	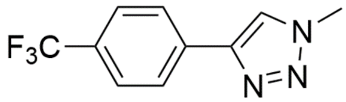
**CN12**	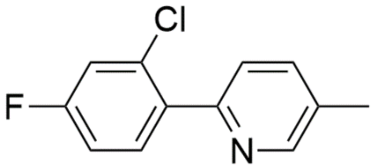	**CN106**	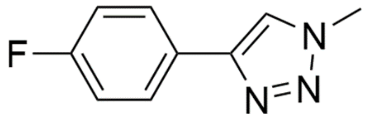
**CN13**	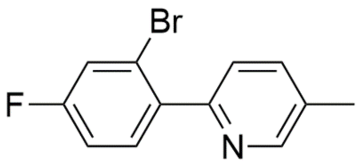	**CN109**	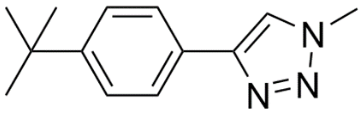

**CN14**	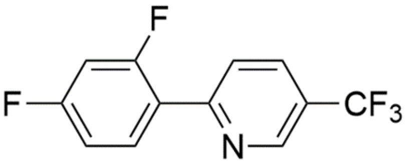	**CN110**	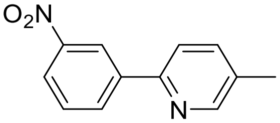
**CN29**	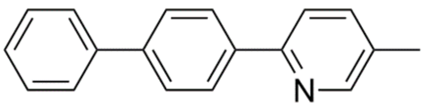	**CN111**	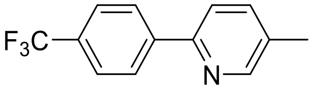
**CN40**	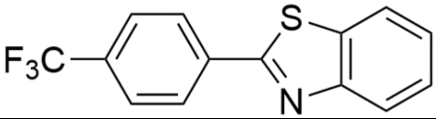	**CN112**	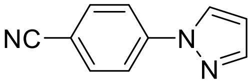
**CN42**	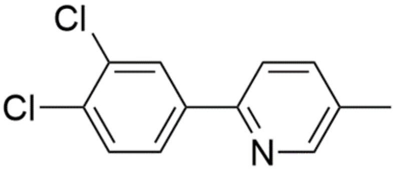	**CN113**	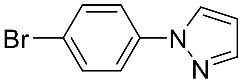
**CN44**	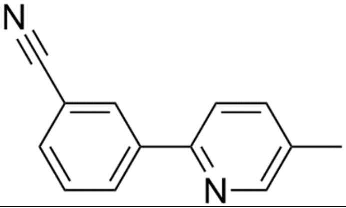	**CN114**	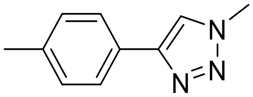
**CN48**	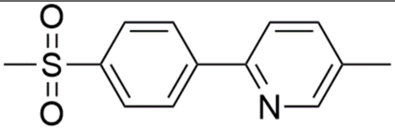	**CN115**	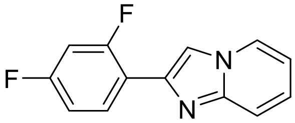
**CN49**	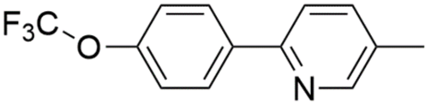	**CN116**	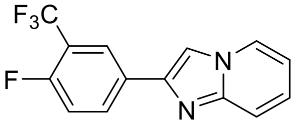
**CN54**	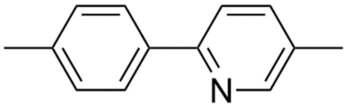	**CN117**	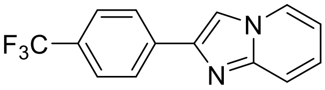
**CN66**	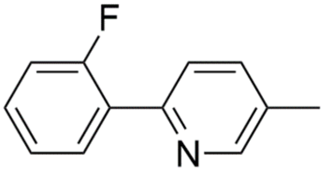	**CN118**	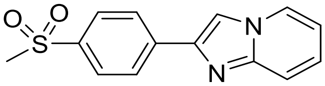

Table S2: All ancillary (N^N) ligands used to form heteroleptic $[Rh(C^\wedge N)_2(N^\wedge N)]^+$ complexes. Structures and corresponding "**N^N**" identification coding are shown.

**NN1**	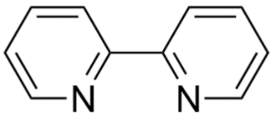	**NN55**	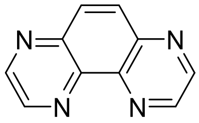
**NN5**	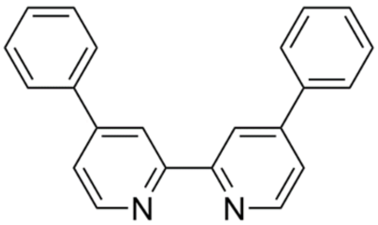	**NN56**	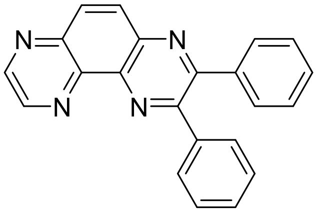
**NN7**	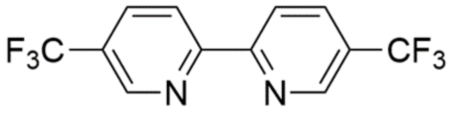	**NN57**	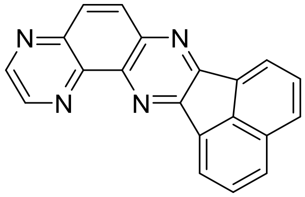
**NN11**	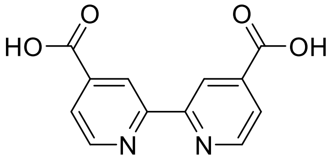	**NN58**	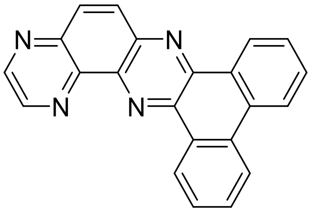
**NN14**	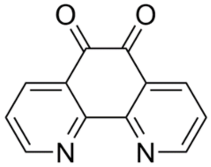	**NN59**	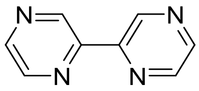
**NN16**	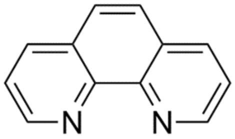	**NN60**	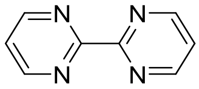
**NN21**	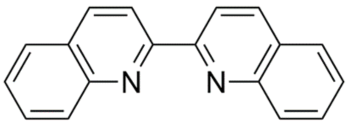	**NN61**	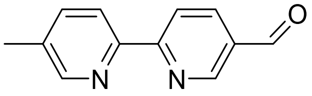
**NN24**	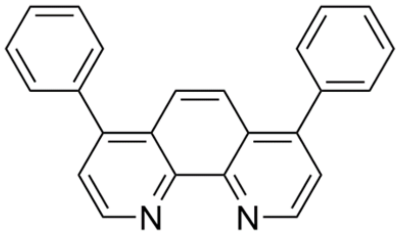	**NN62**	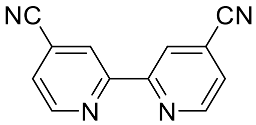

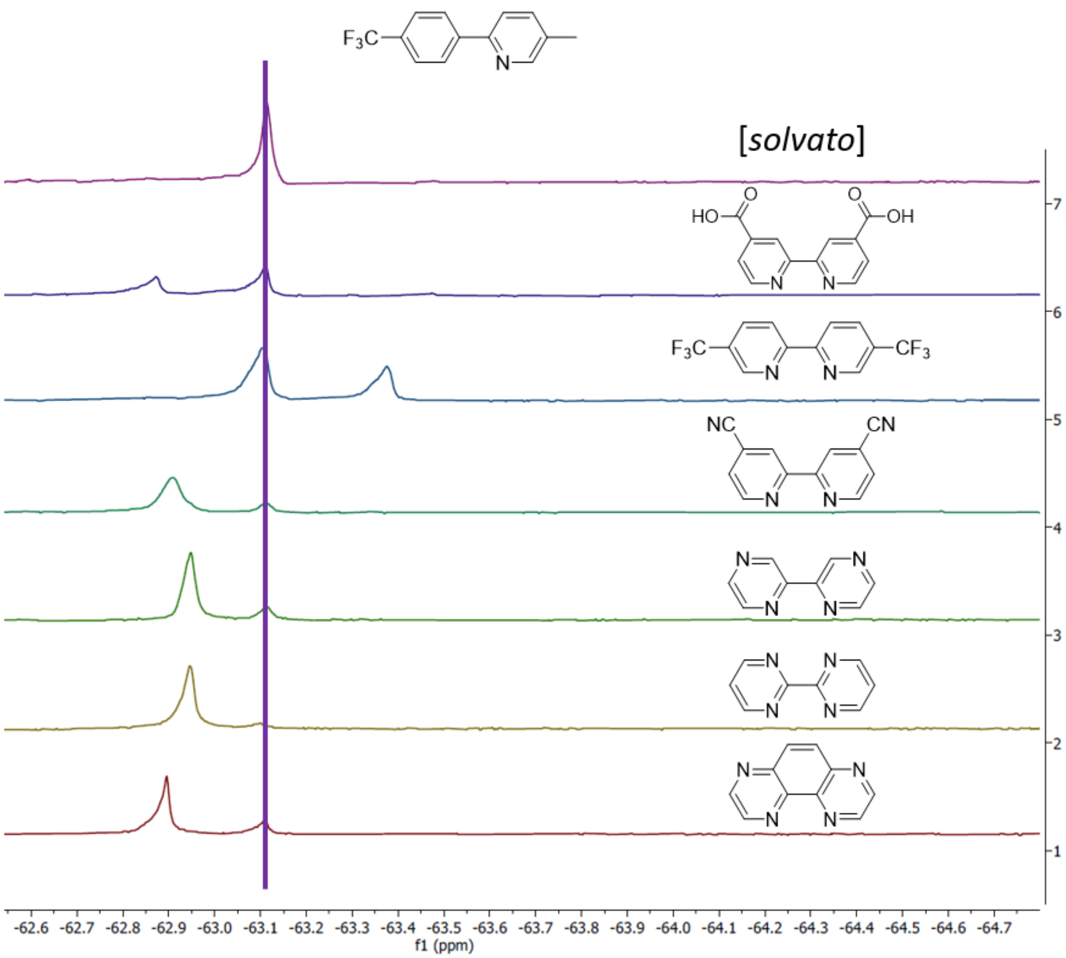

Figure S1: ^{19}F NMR spectra of heteroleptic Rh(III) complexes containing **CN111** with various diimine ancillary ligands. The *solvato* complexes represents the baseline, and the observed changes in chemical shifts indicated ancillary ligand coordination. Ancillary ligand structures are depicted for convenience.

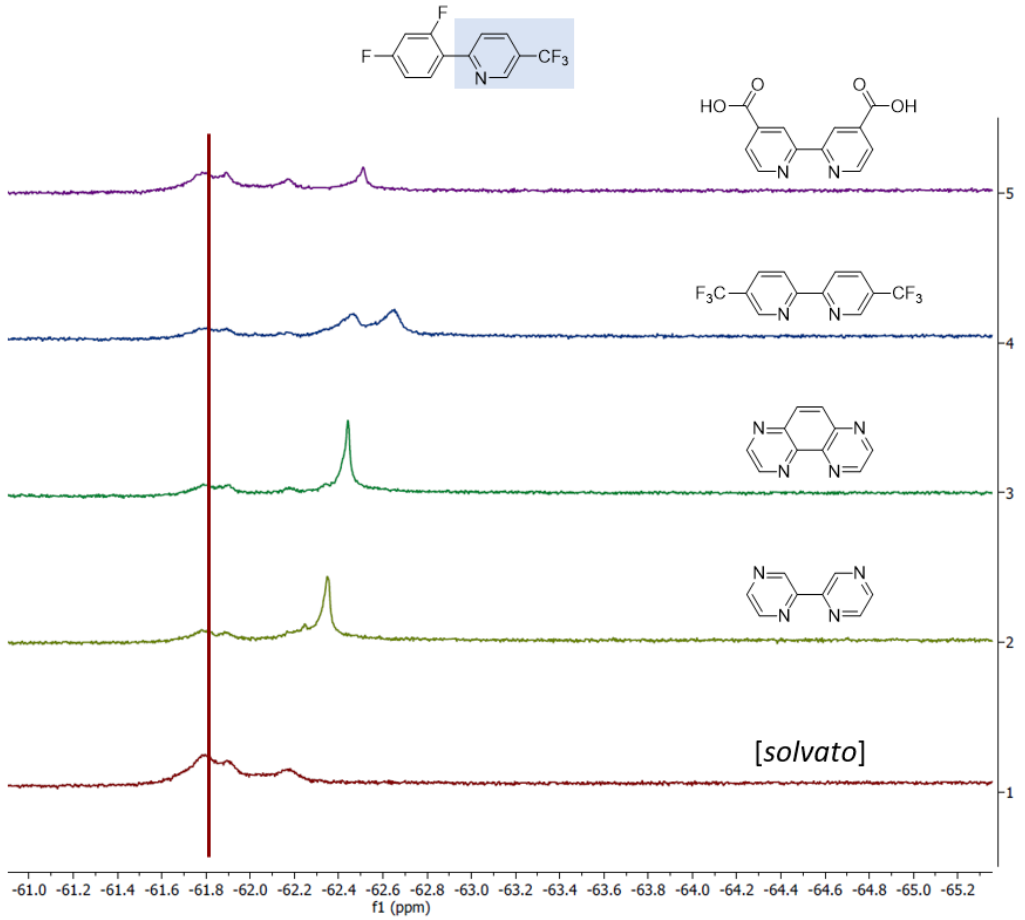

Figure S2: ^{19}F NMR spectra of heteroleptic Rh(III) complexes containing **CN14** with various diimine ancillary ligands. The aryl-CF_3 region is shown here. The *solvato* complexes represents the baseline, and the observed changes in chemical shifts indicated ancillary ligand coordination. Ancillary ligand structures are depicted for convenience.

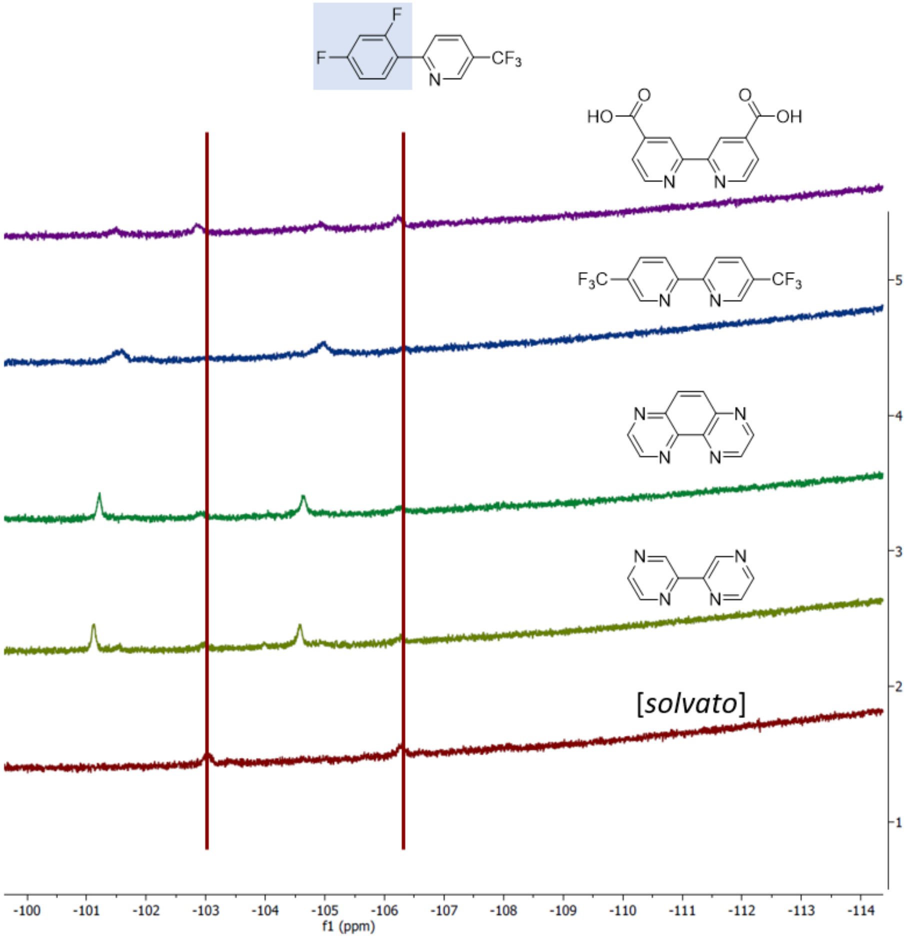

Figure S3: ^{19}F NMR spectra of heteroleptic Rh(III) complexes containing **CN14** with various diimine ancillary ligands. The aryl-F region is shown here. The *solvato* complexes represents the baseline, and the observed changes in chemical shifts indicated ancillary ligand coordination. Ancillary ligand structures are depicted for convenience.

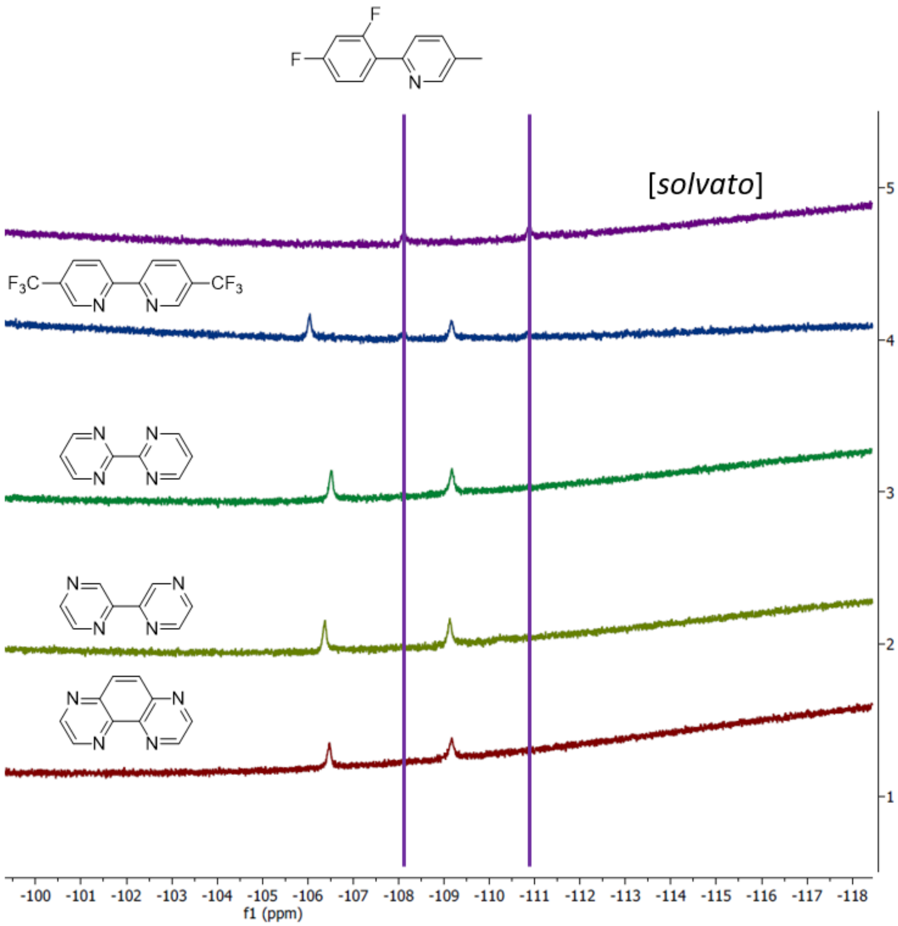

Figure S4: ^{19}F NMR spectra of heteroleptic Rh(III) complexes containing **CN11** with various diimine ancillary ligands. The *solvato* complexes represents the baseline, and the observed changes in chemical shifts indicated ancillary ligand coordination. Ancillary ligand structures are depicted for convenience.

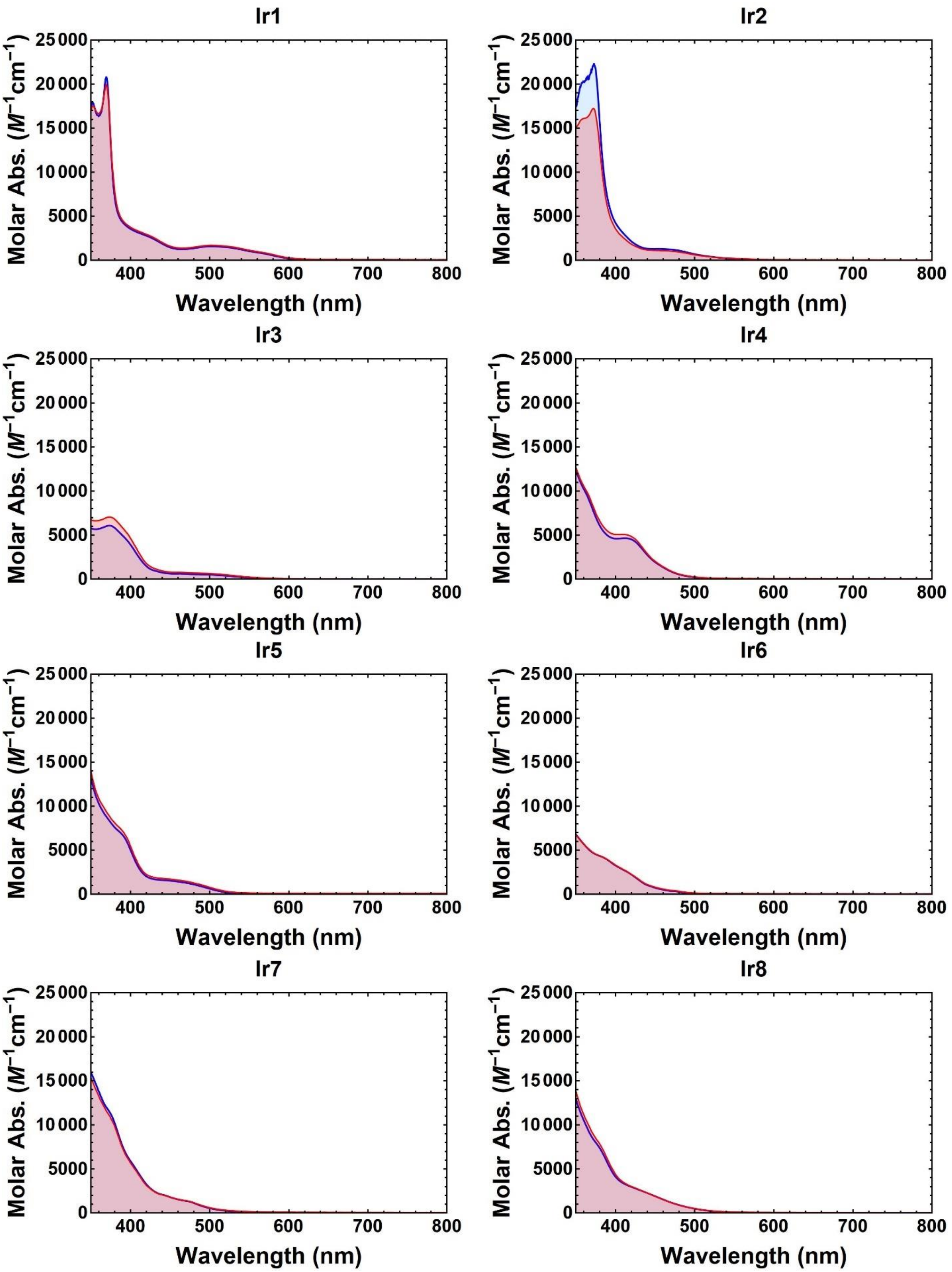

Ir1
Ir2
Ir3
Ir4
Ir5
Ir6
Ir7
Ir8
Molar Abs. (M^{-1}cm^{-1})
0
5000
10 000
15 000
20 000
25 000
Wavelength (nm)
400
500
600
700
800

Ir9

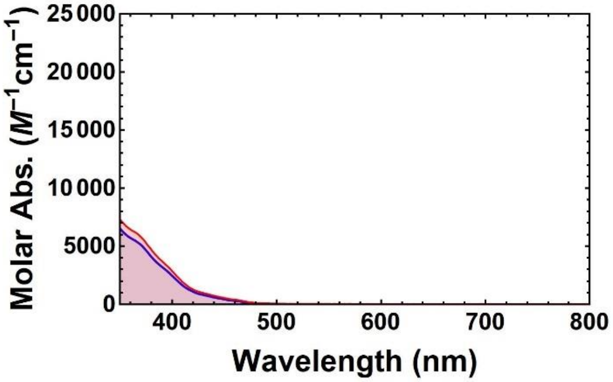

Ir10

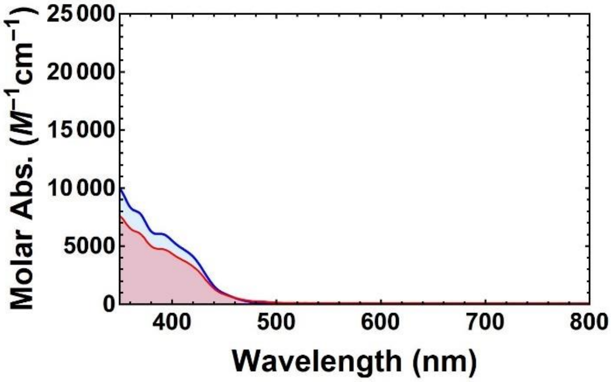

Ir11

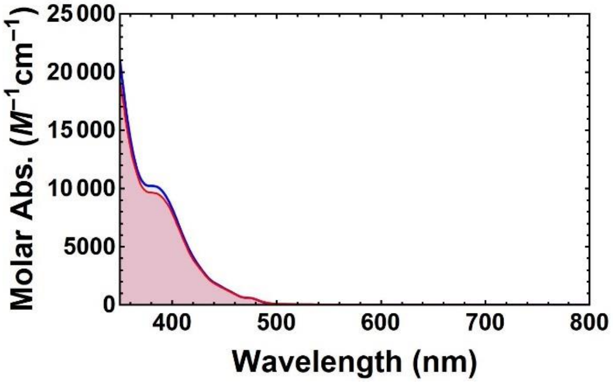

Ir12

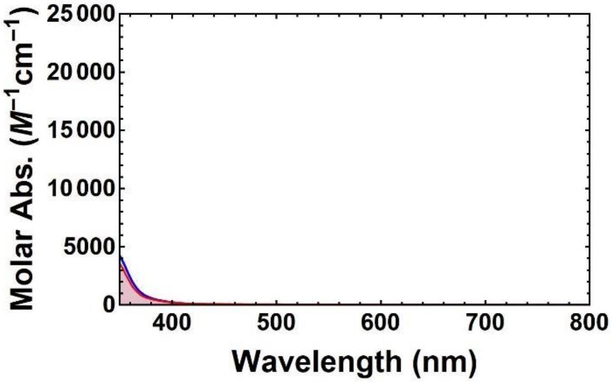

Ir13

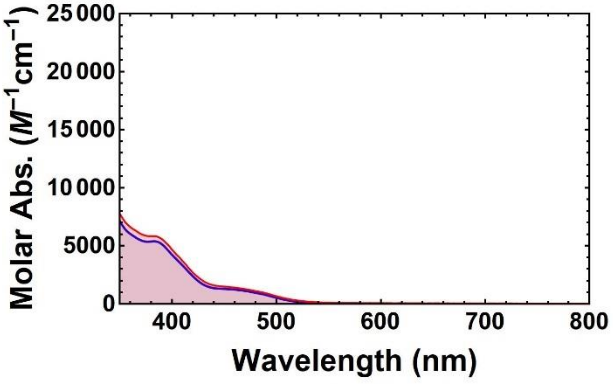

Ir14

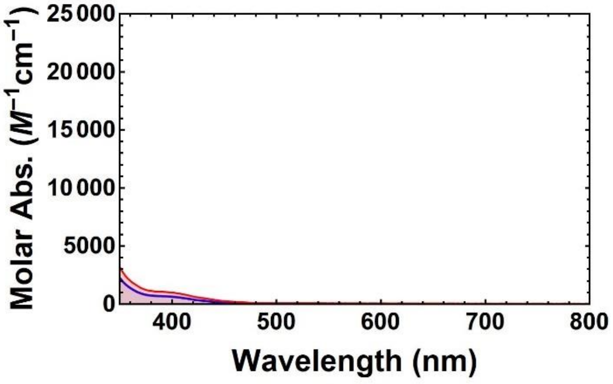

Ir15

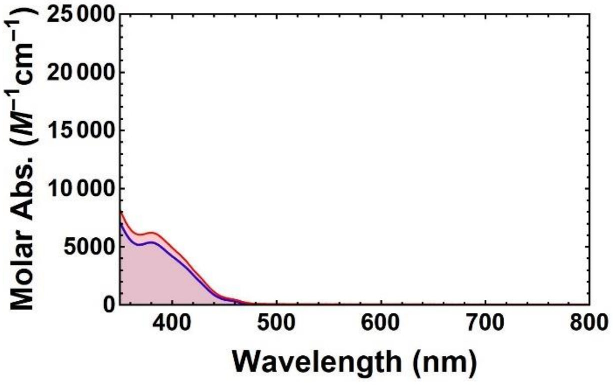

Ir16

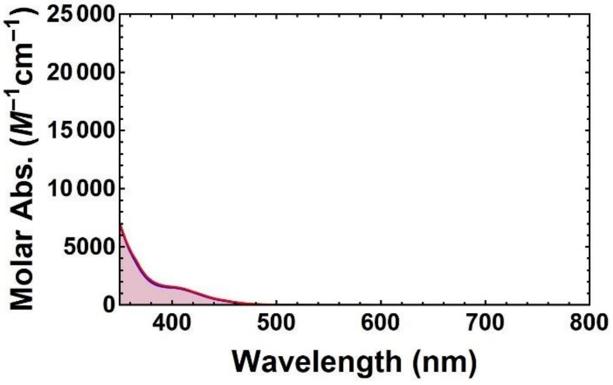

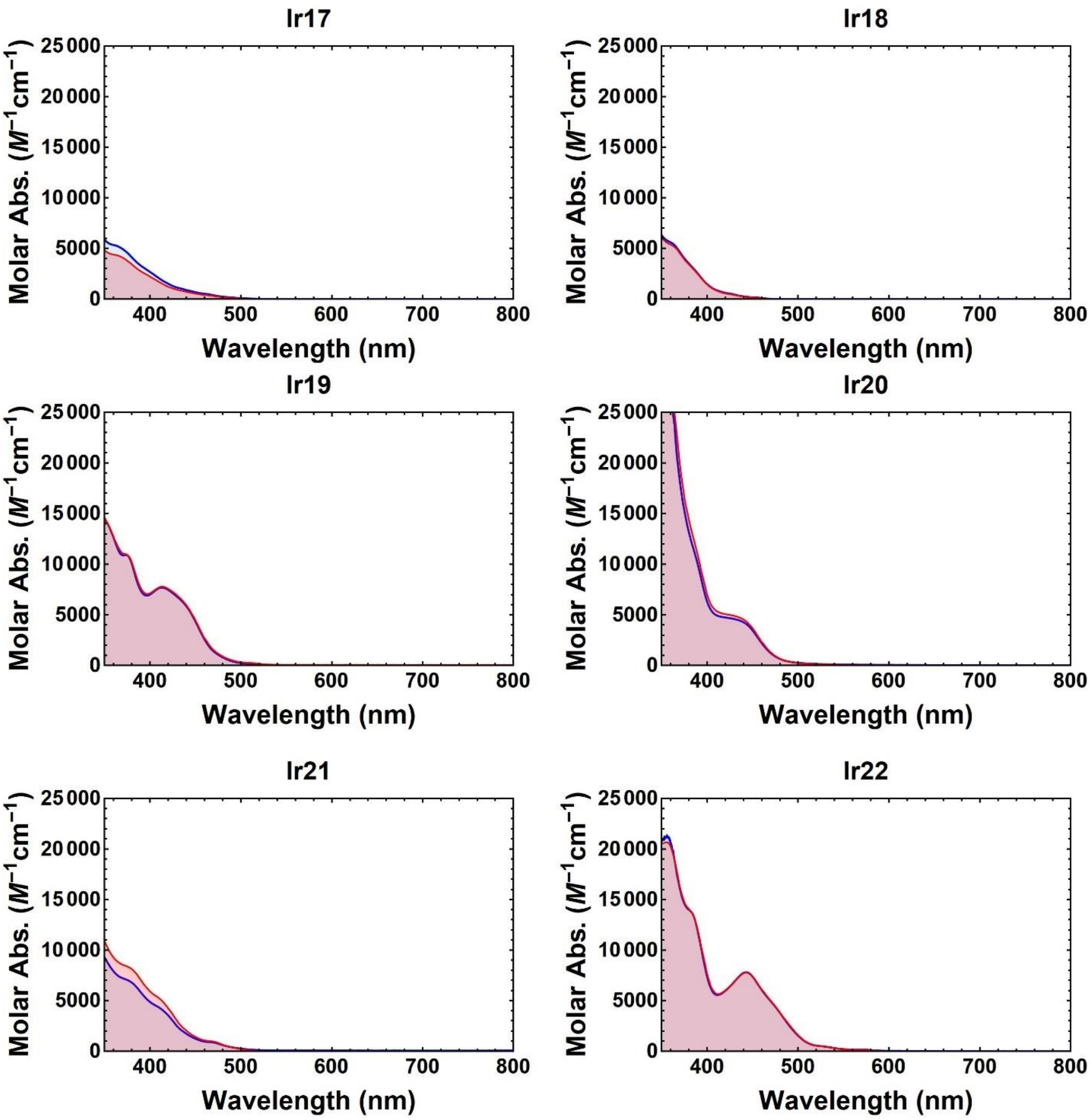

Figure S5: Overlayed UV-visible absorption spectra depicting the ThermoFisher Scientific microplate reader (blue) spectra and the spectra measured using a Shimadzu UV-1800 spectrophotometer (red) for 22 heteroleptic $[Ir(C^\wedge N)_2(N^\wedge N)]^+$ complexes previously measured. Naming systems for these Ir(III) complexes is taken from previously reported work.[8]

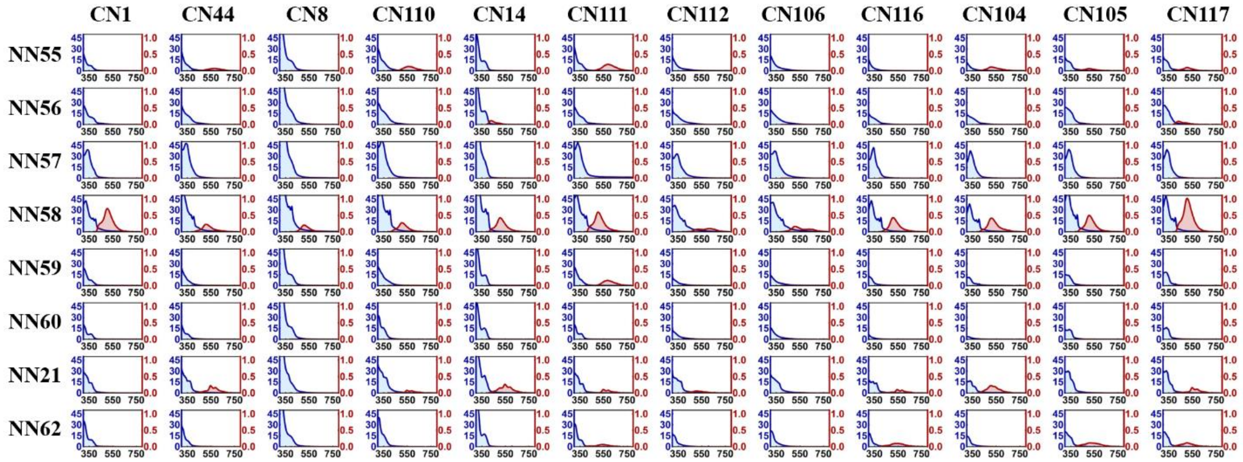

Figure S6: High-throughput screened absorption and deaerated emission spectra in propylene glycol. Measured molar absorptivity is 1000X the depicted values and emission spectra are normalized across the entire set. All **CN/NN** numbers are shown.

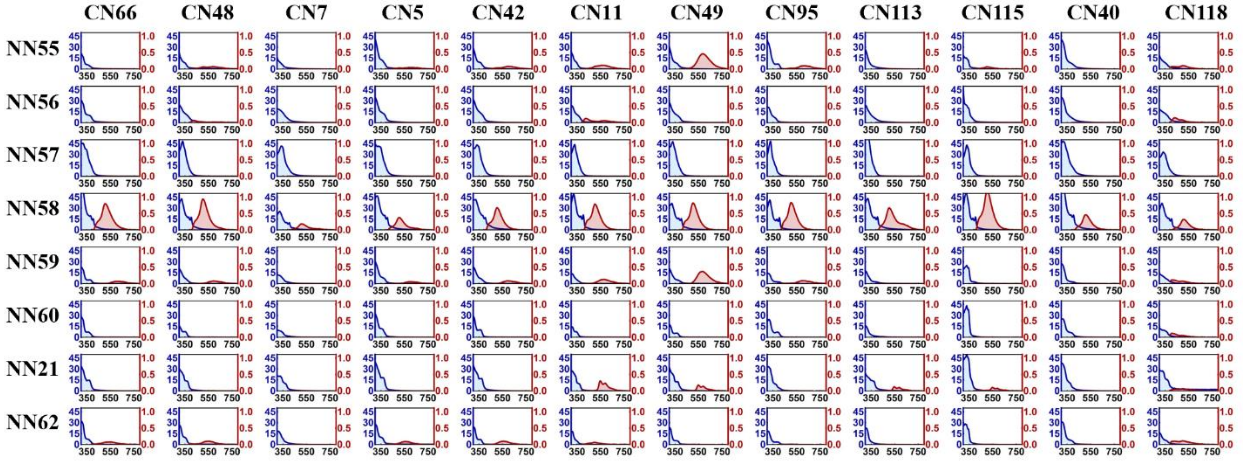

Figure S7: High-throughput screened absorption and deaerated emission spectra in propylene glycol. Measured molar absorptivity is 1000X the depicted values and emission spectra are normalized across the entire set. All **CN/NN** numbers are shown.

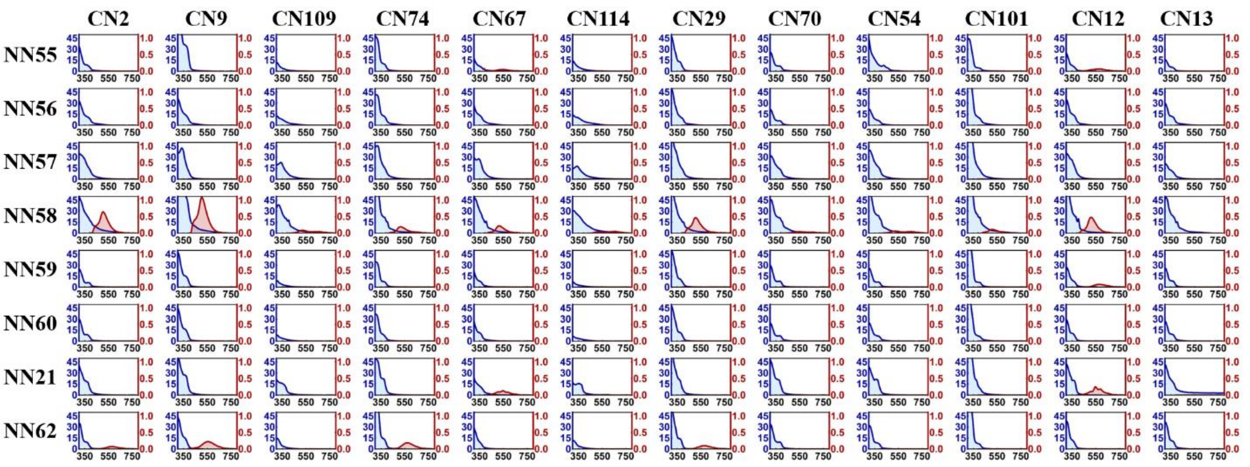

Figure S8: High-throughput screened absorption and deaerated emission spectra in propylene glycol. Measured molar absorptivity is 1000X the depicted values and emission spectra are normalized across the entire set. All **CN/NN** numbers are shown.

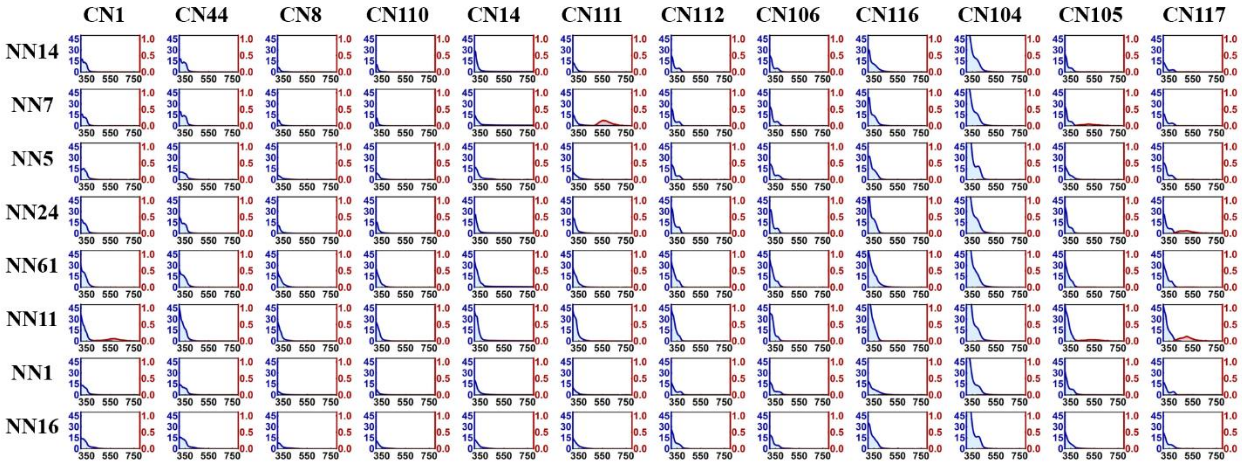

Figure S9: High-throughput screened absorption and deaerated emission spectra in propylene glycol.. Measured molar absorptivity is 1000X the depicted values and emission spectra are normalized across the entire set. All **CN/NN** numbers are shown.

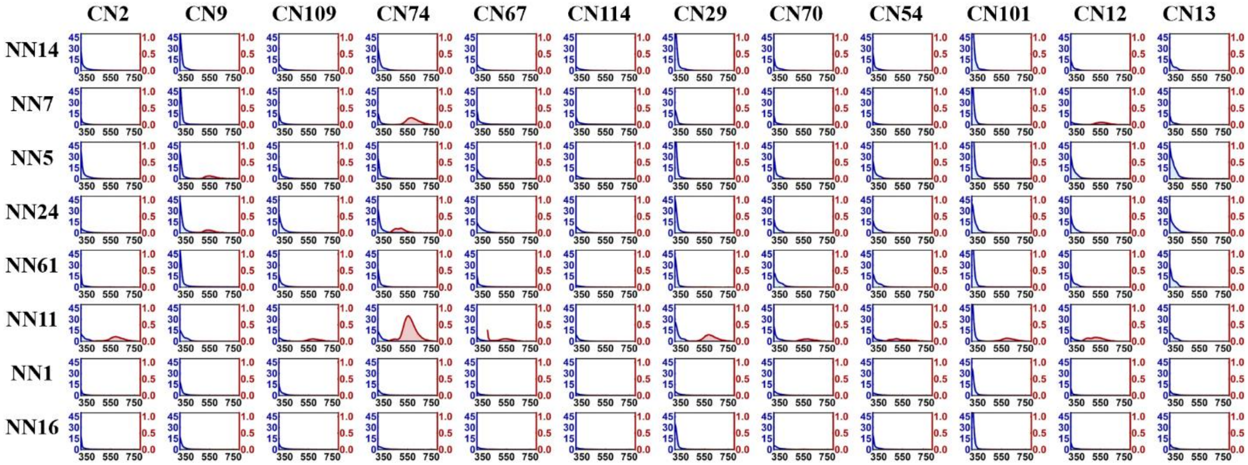

Figure S10: High-throughput screened absorption and deaerated emission spectra in propylene glycol. Measured molar absorptivity is 1000X the depicted values and emission spectra are normalized across the entire set. All **CN/NN** numbers are shown.

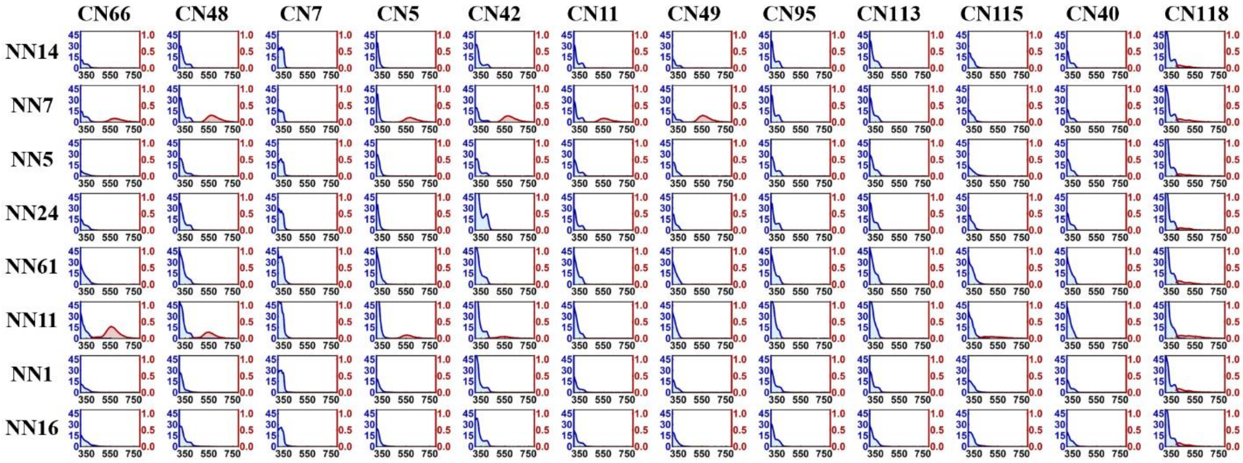

Figure S11: High-throughput screened absorption and deaerated emission spectra in propylene glycol. Measured molar absorptivity is 1000X the depicted values and emission spectra are normalized across the entire set. All **CN/NN** numbers are shown.

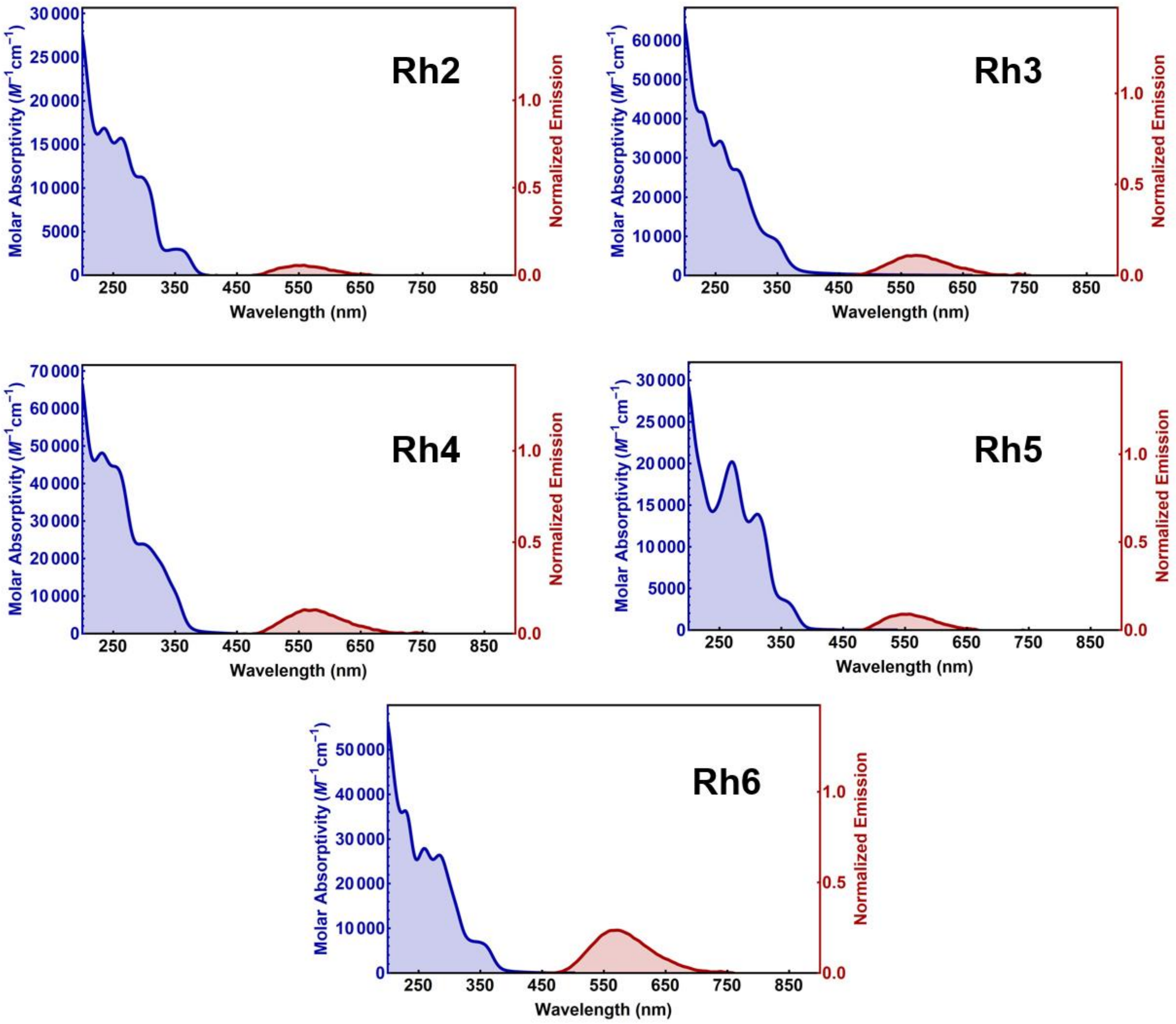

Figure S12: UV-vis absorption and emission spectra of **Rh2 – Rh6**. Emission spectra are normalized across the set to highlight differences in luminescent intensity.

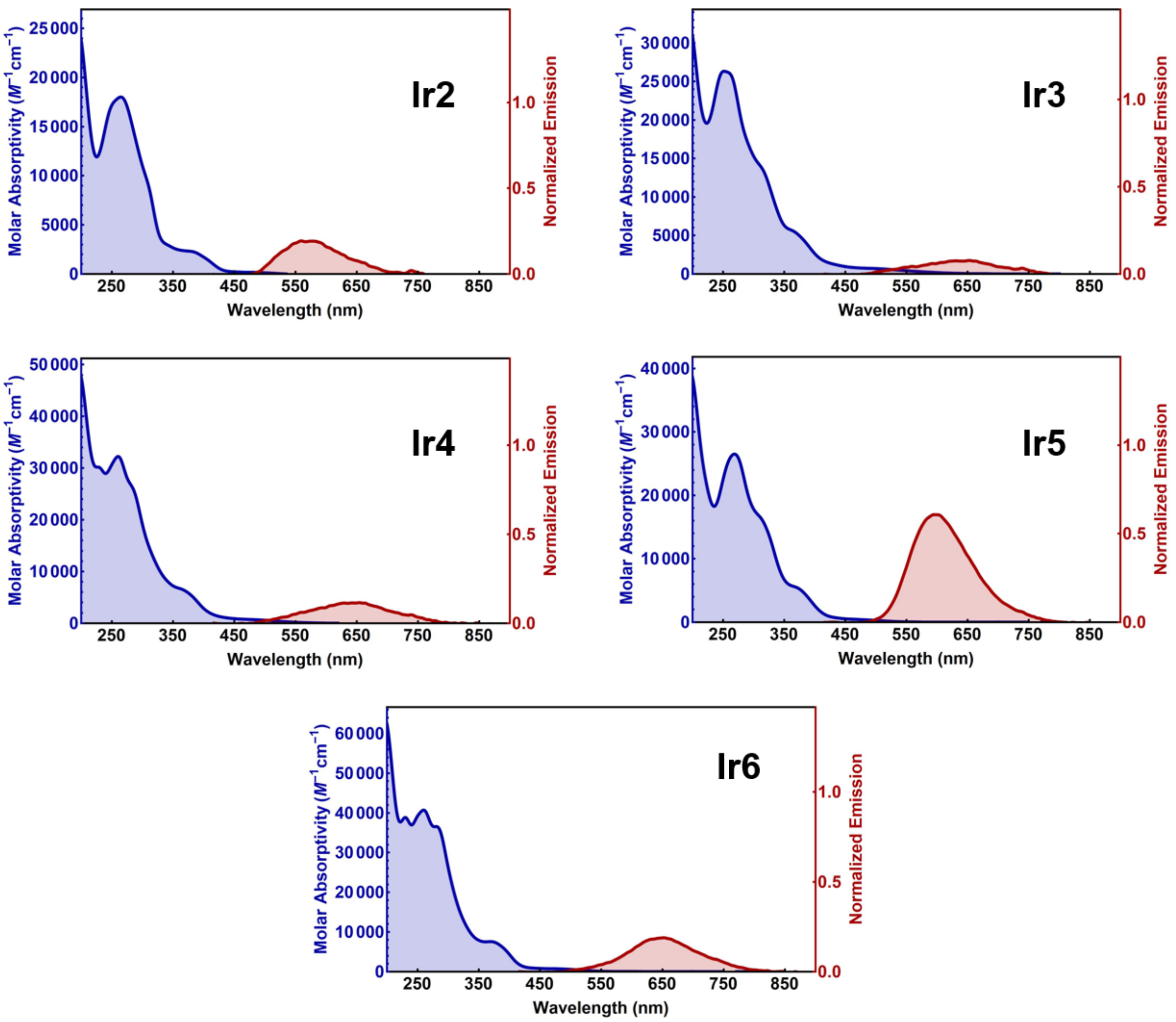

Figure S13: UV-vis absorption and emission spectra of **Ir2 – Ir6**. Emission spectra are normalized across the set to highlight differences in luminescent intensity.

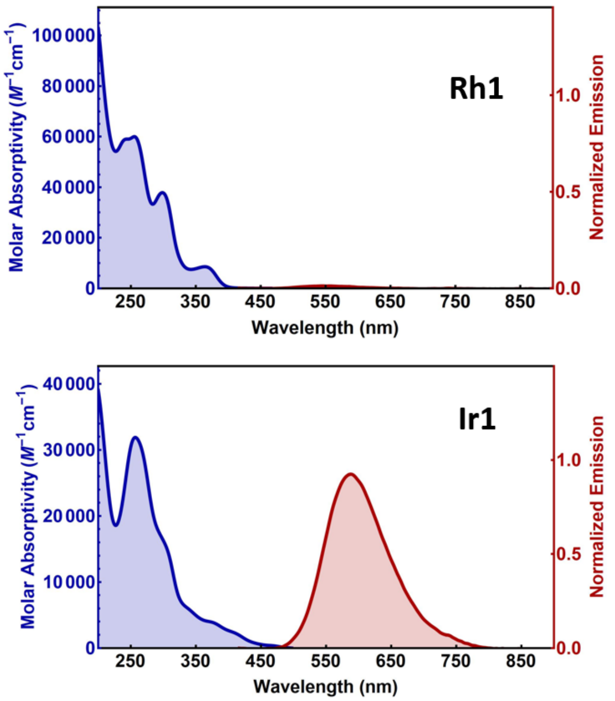

Figure S14: UV-vis absorption and emission spectra of the parent Ir(III) and Rh(III) complexes (containing 2-Phenylpyridine (ppy) and 2,2'-bipyridine (bpy)). Emission spectra are normalized across the set to highlight difference in luminescent intensity.

Table S3: Tabulated photophysical features between the HTSS (Green) and traditional synthesis (Blue).

Complex	Em_{max} (eV)	τ_o (μs)	Em_{max} (eV)	τ_o (μs)
**Rh2**	2.16	0.27	2.22	0.14
**Rh3**	2.14	0.52	2.22	0.62
**Rh4**	2.12	0.67	2.16	0.73
**Rh5**	2.23	0.65	2.23	0.41
**Rh6**	2.09	0.54	2.17	0.75

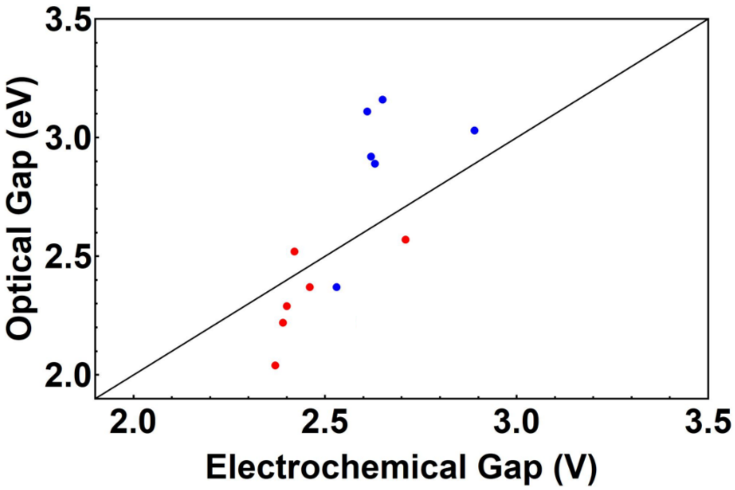

Figure S15: Correlations between the optical gap and the electrochemical gap for the 6 Rh(III) (blue) and Ir(III) (Red) studied here. The black line represents the y = x line.

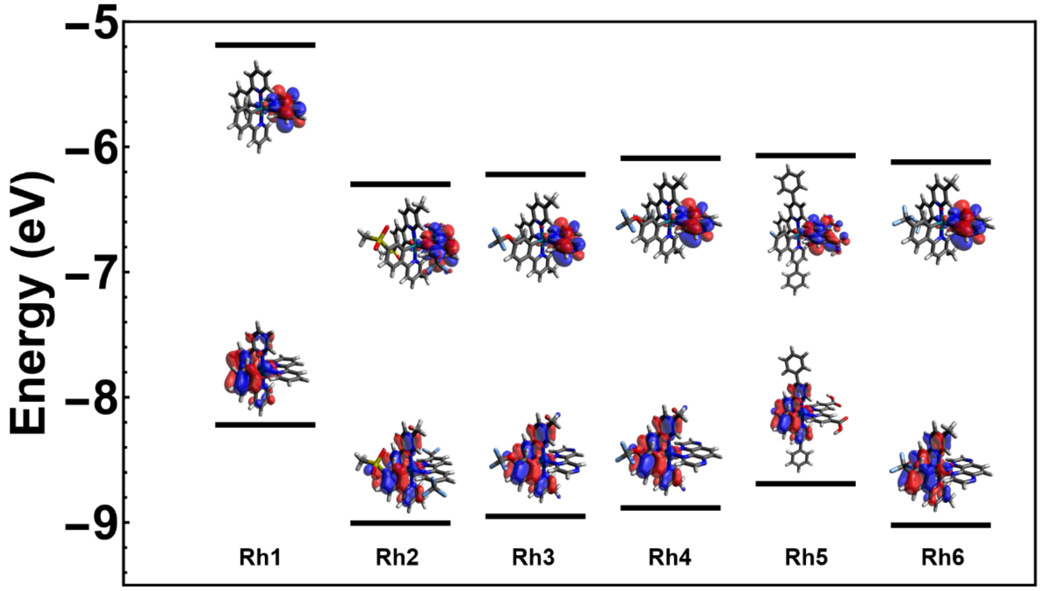

Figure S16: Frontier orbital diagrams for **Rh1** – **Rh6** (left to right) with HOMO and LUMO orbital distributions.

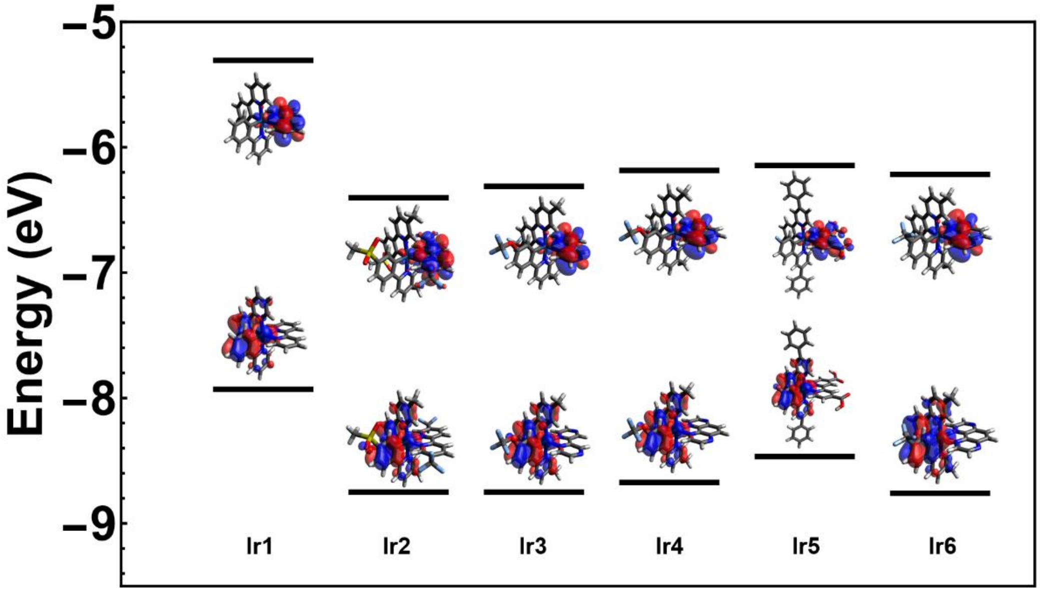

Figure S17: Frontier orbital diagrams for **Ir1 – Ir6** (left to right) with HOMO and LUMO orbital distributions.

Table S4: DFT calculated Rh(III)/Ir(III) coordination bond lengths.

Complex	Metal-N bond (N^N Ligand)	Metal-N bond (C^N Ligand)	Metal-C bond (C^N Ligand)
**Rh1**	2.220 Å	2.084 Å	2.016 Å
**Ir1**	2.175 Å	2.081 Å	2.030 Å
**Rh2**	2.210 Å	2.084 Å	2.012 Å
**Ir2**	2.169 Å	2.081 Å	2.026 Å
**Rh3**	2.201 Å	2.084 Å	2.017 Å
**Ir3**	2.157 Å	2.081 Å	2.032 Å
**Rh4**	2.219 Å	2.034 Å	2.015 Å
**Ir4**	2.173 Å	2.031 Å	2.029 Å
**Rh5**	2.197 Å	2.079 Å	2.018 Å
**Ir5**	2.157 Å	2.077 Å	2.032 Å
**Rh6**	2.220 Å	2.084 Å	2.016 Å
**Ir6**	2.175 Å	2.081 Å	2.030 Å

Table S5: Formation and stability of radical intermediate **Rh4** after reductive quenching. Top 4 pictures indicate the initial color of the sample and the observed change in color as a function of blue illumination time (T). The bottom four pictures are photos taken without blue illumination and highlight the stability of the formed radical after 40 minutes of blue light irradiation.

**Blue illumination time**			
Time = 0 min	T = 5 min	T =10 min	T = 40 min
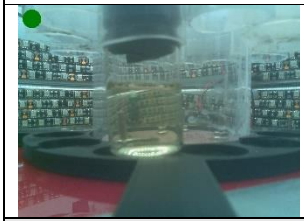	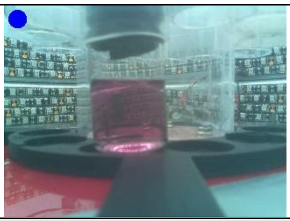	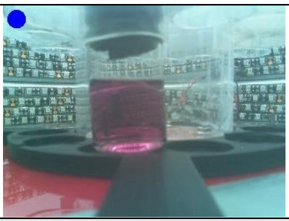	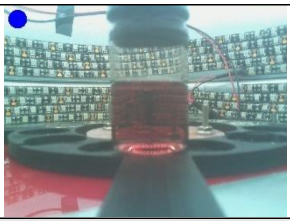
**Stability of radical intermediate**			
Time = 0 min	T = 30 min	T = 120 min	T = 18 hours
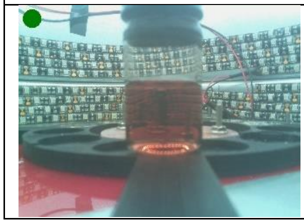	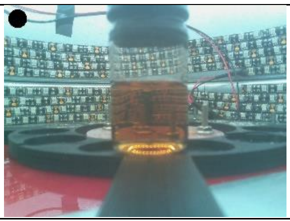	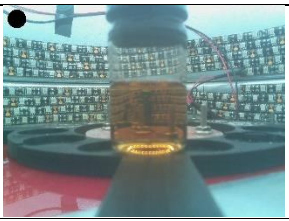	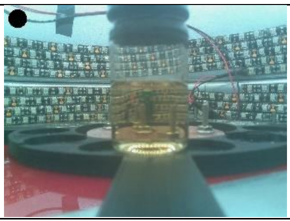

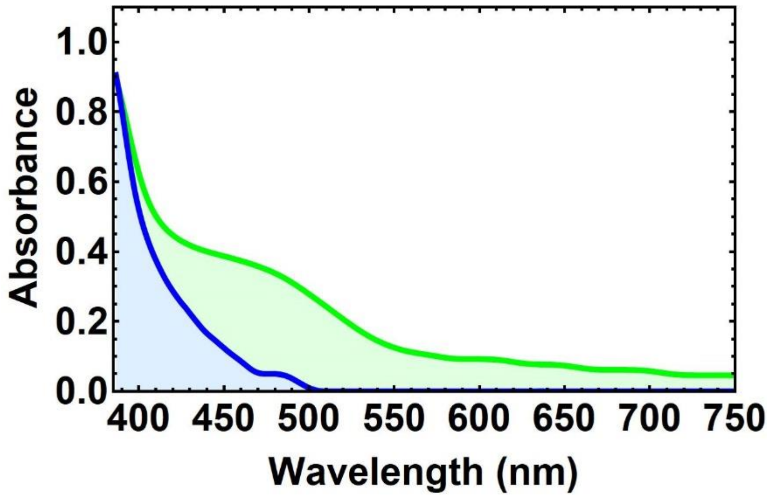

Figure S18: Normalized deaerated UV-vis spectra for **Rh4** in the presence of 0.18 M triethanolamine (TEOA) before (blue) and after (green) irradiation with 445 nm light for 10 minutes. This concentration of TEOA replicates the conditions use for the hydrogen evolution studies. Bathochromic shift is assigned to the absorption of the reduced radical intermediate responsible for transferring and electron the water reduction catalyst.

Table S6: Control experiments in water reduction systems

Conditions	Measured H_2(g) after 2 days of illumination (µmoles)
Optimized conditions	14.0
No TEOA	0.0
No K_2PdCl_4	0.24
No Rh	0.0

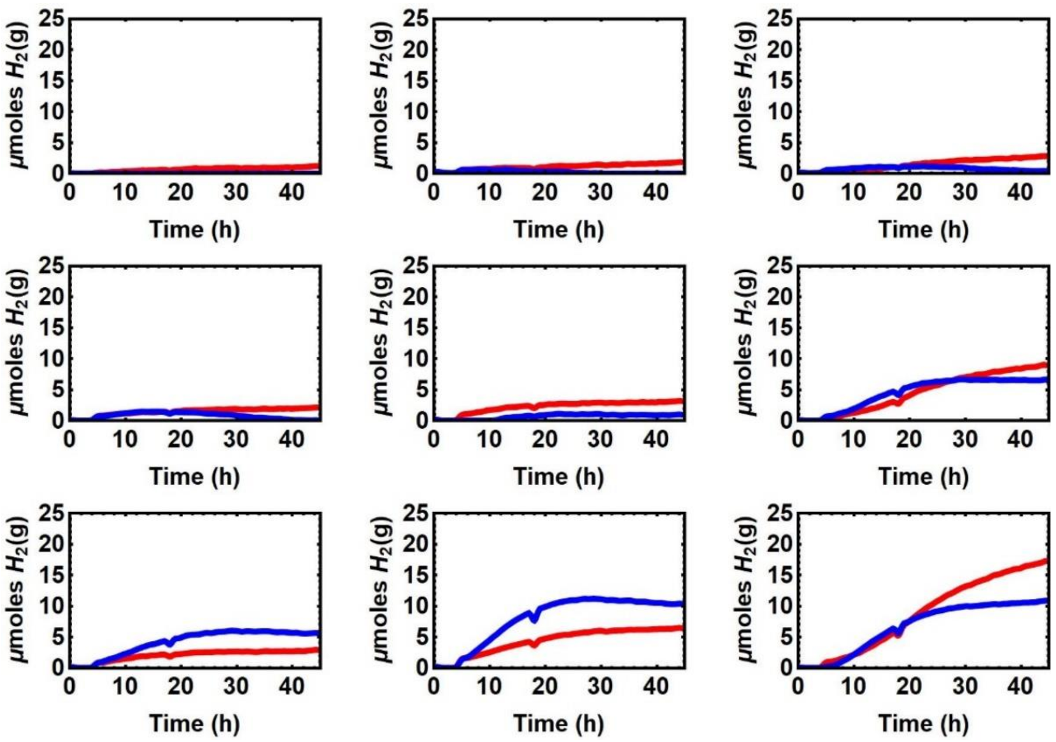

Figure S19: Overlayed plots of hydrogen evolution traces for **Rh4** (Blue) and **Rh6** (Red). Concentration of each photocatalyst increased from 0.25 mM to 0.5 mM to 1.0 mM across the columns. Concentration of K_2PdCl_4 precatalyst increased down the rows from 0.01 mM to 0.05 mM then 0.1 mM.

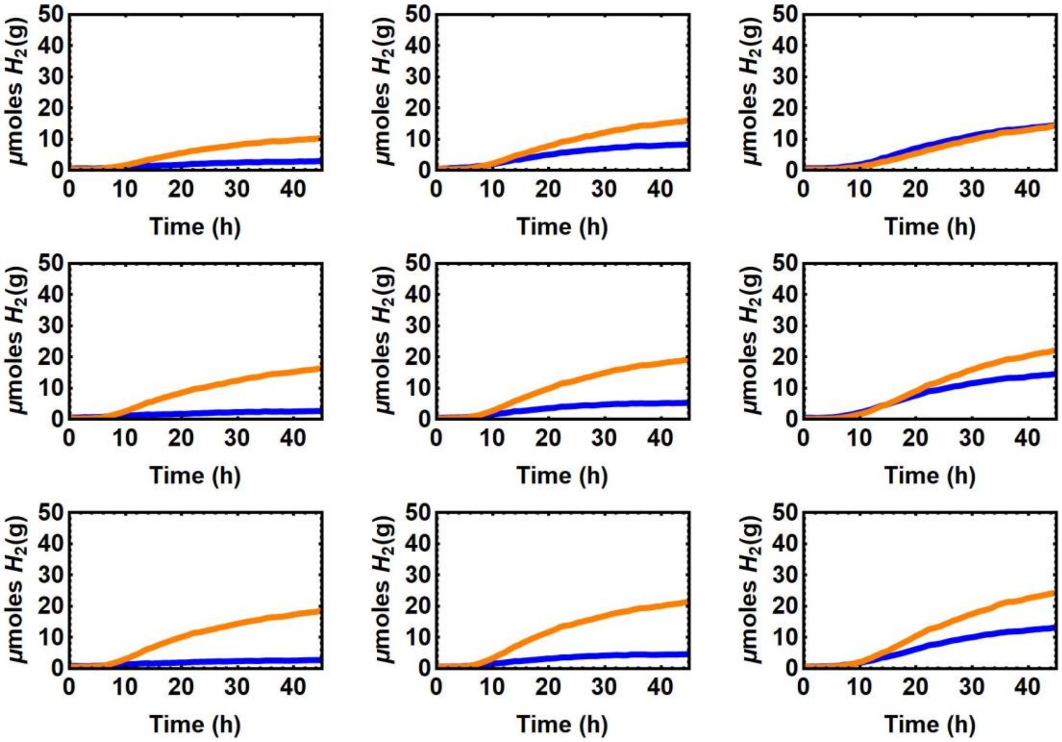

Figure S20: Overlayed plots of hydrogen evolution traces for **Rh4** (blue) and **Ir4** (orange). Concentration of each photocatalyst increased from 0.25 mM to 0.5 mM to 1.0 mM across the columns. Concentration of K_2PdCl_4 precatalyst increased down the rows from 0.025 mM to 0.05 mM then 0.075 mM.

NMR Characterization for Traditionally Synthesized Complexes

Rh2:– expected mass 887.698 – observed mass 887.0638

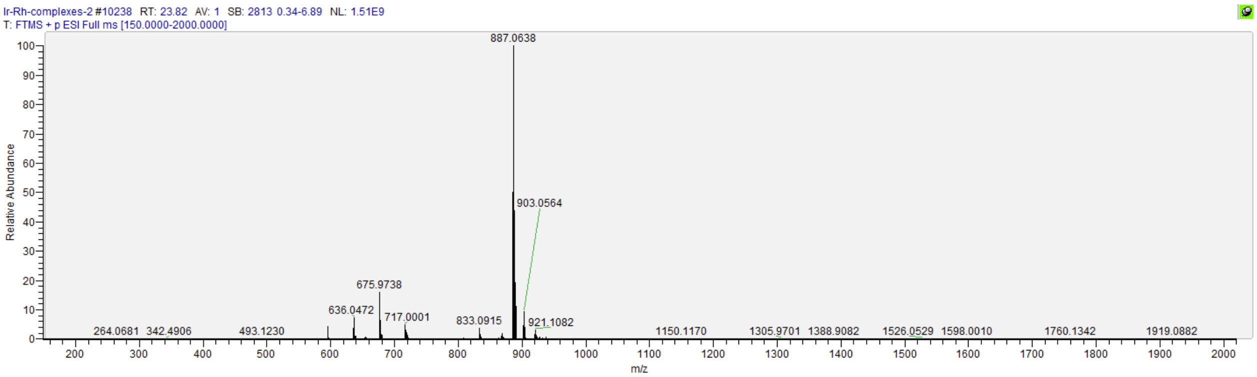

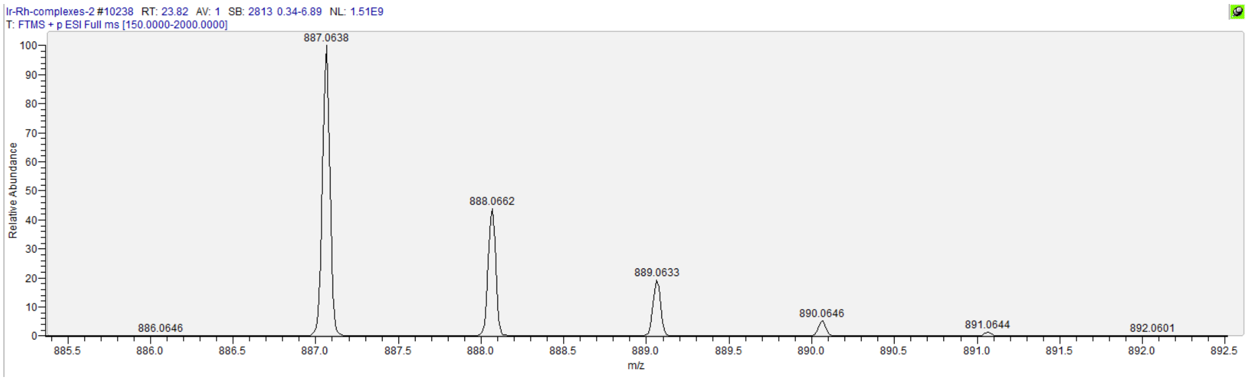

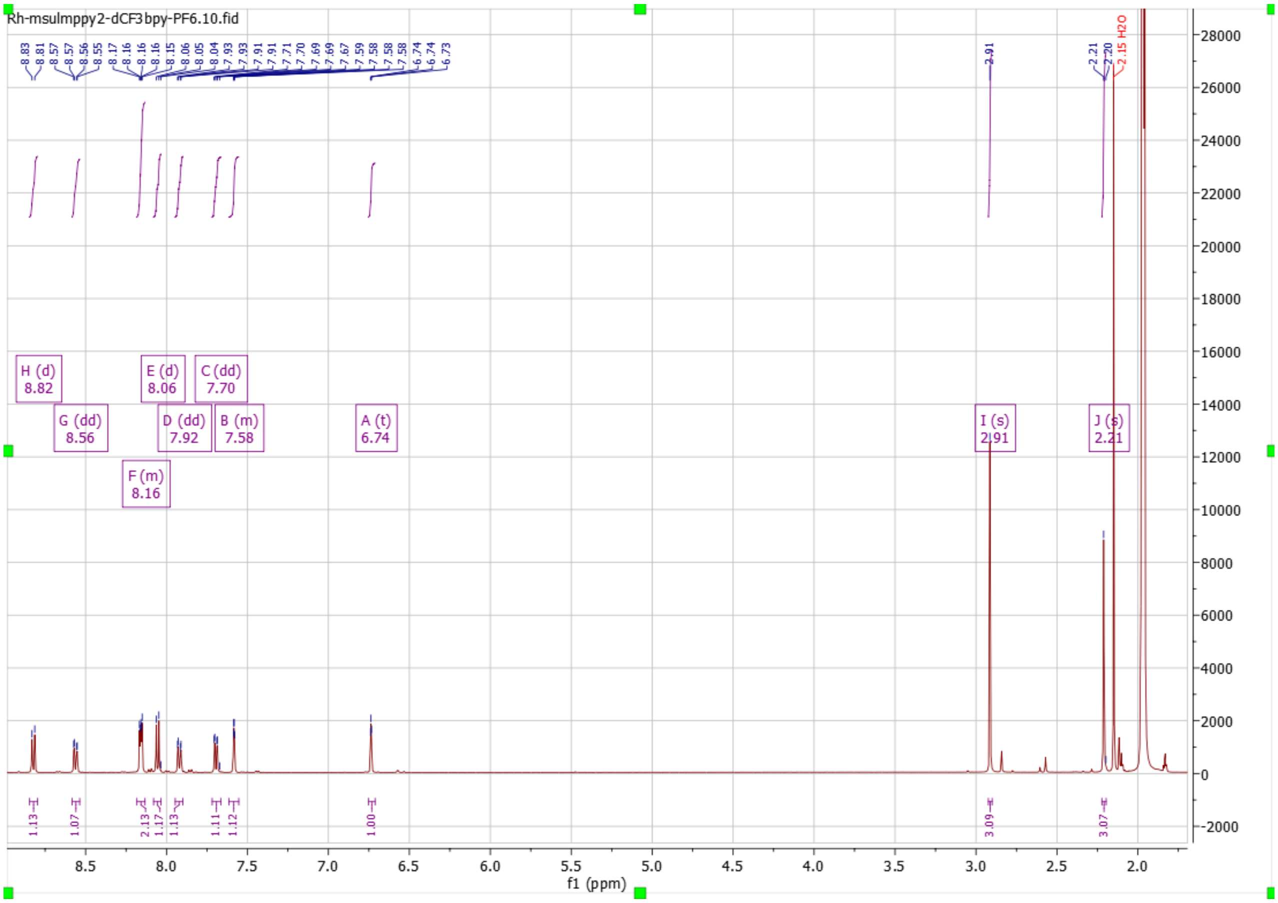

^{1}H NMR (500 MHz, Acetonitrile-d_3) δ 8.82 (d, *J* = 8.5 Hz, 2H), 8.56 (dd, *J* = 8.6, 2.2 Hz, 2H), 8.18 – 8.13 (m, 4H), 8.06 (d, *J* = 8.2 Hz, 2H), 7.92 (dd, *J* = 8.4, 2.0 Hz, 2H), 7.70 (dd, *J* = 8.2, 1.9 Hz, 2H), 7.62 – 7.55 (m, 2H), 6.74 (t, *J* = 1.4 Hz, 2H), 2.91 (s, 6H), 2.21 (s, 6H).

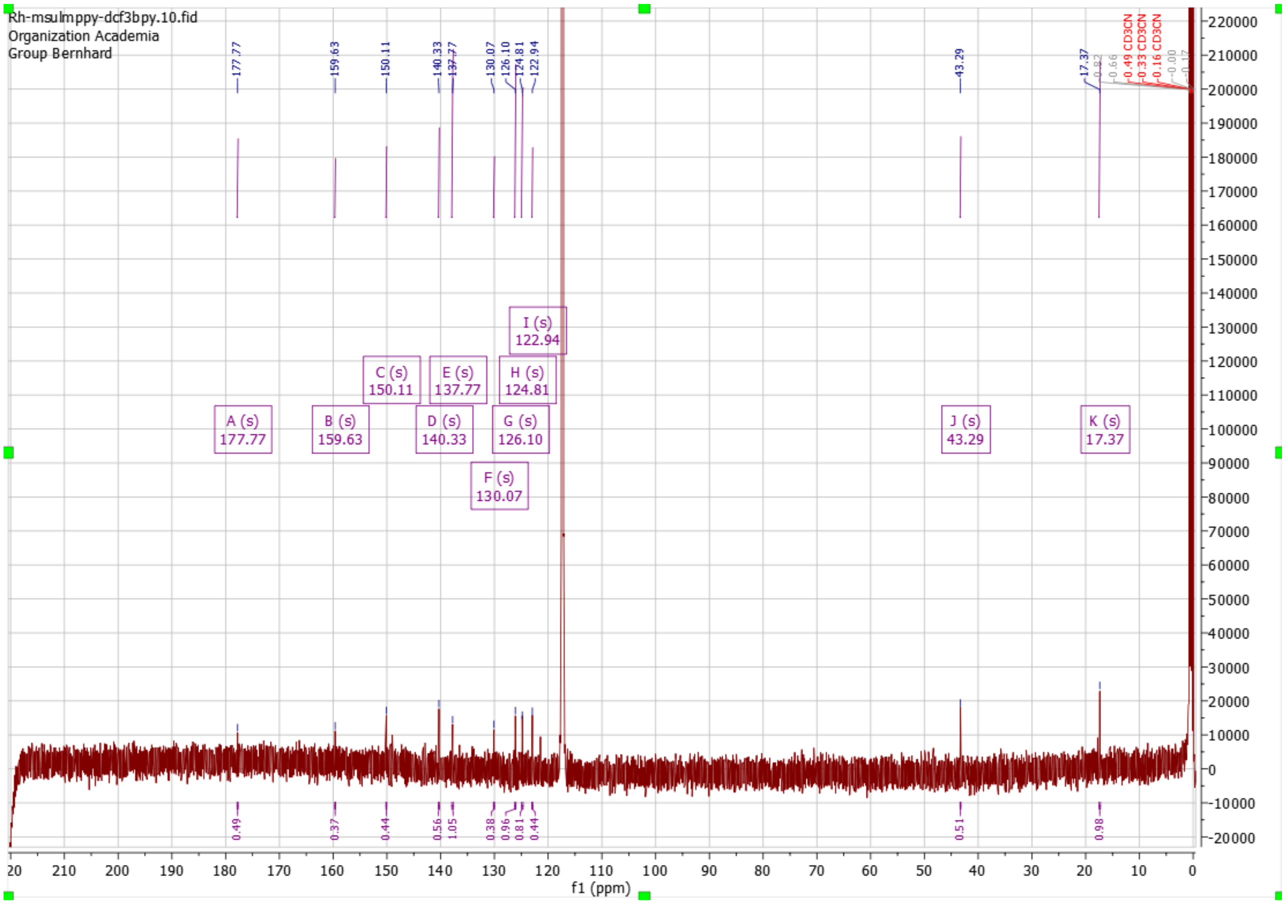

^{13}C NMR (126 MHz, Acetonitrile-d_3) δ 177.77, 159.63, 150.11, 140.33, 137.77, 130.07, 126.10, 124.81, 122.94, 43.29, 17.37.

Ir2: expected mass 977.009 – observed mass 977.1190

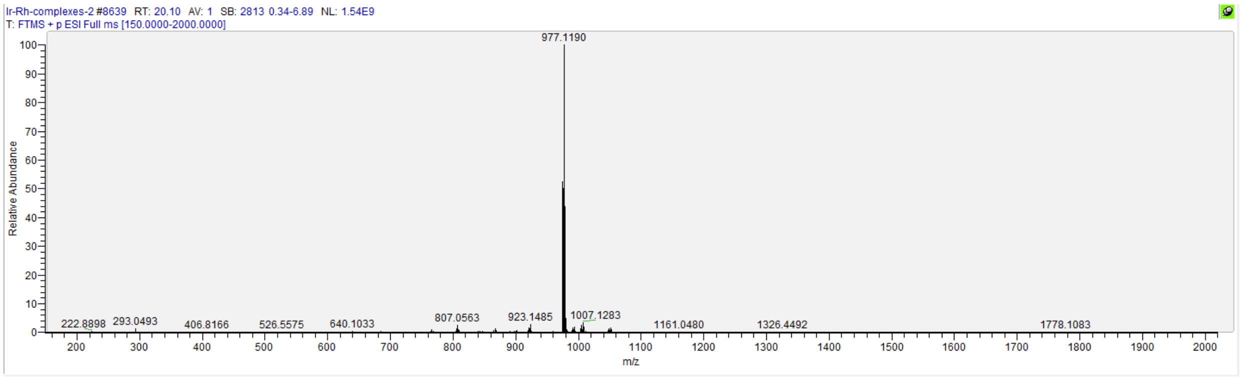

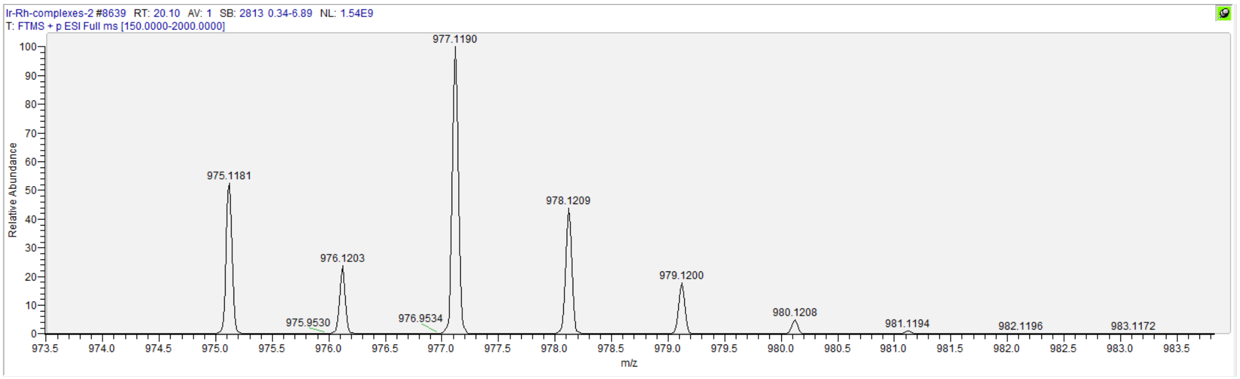

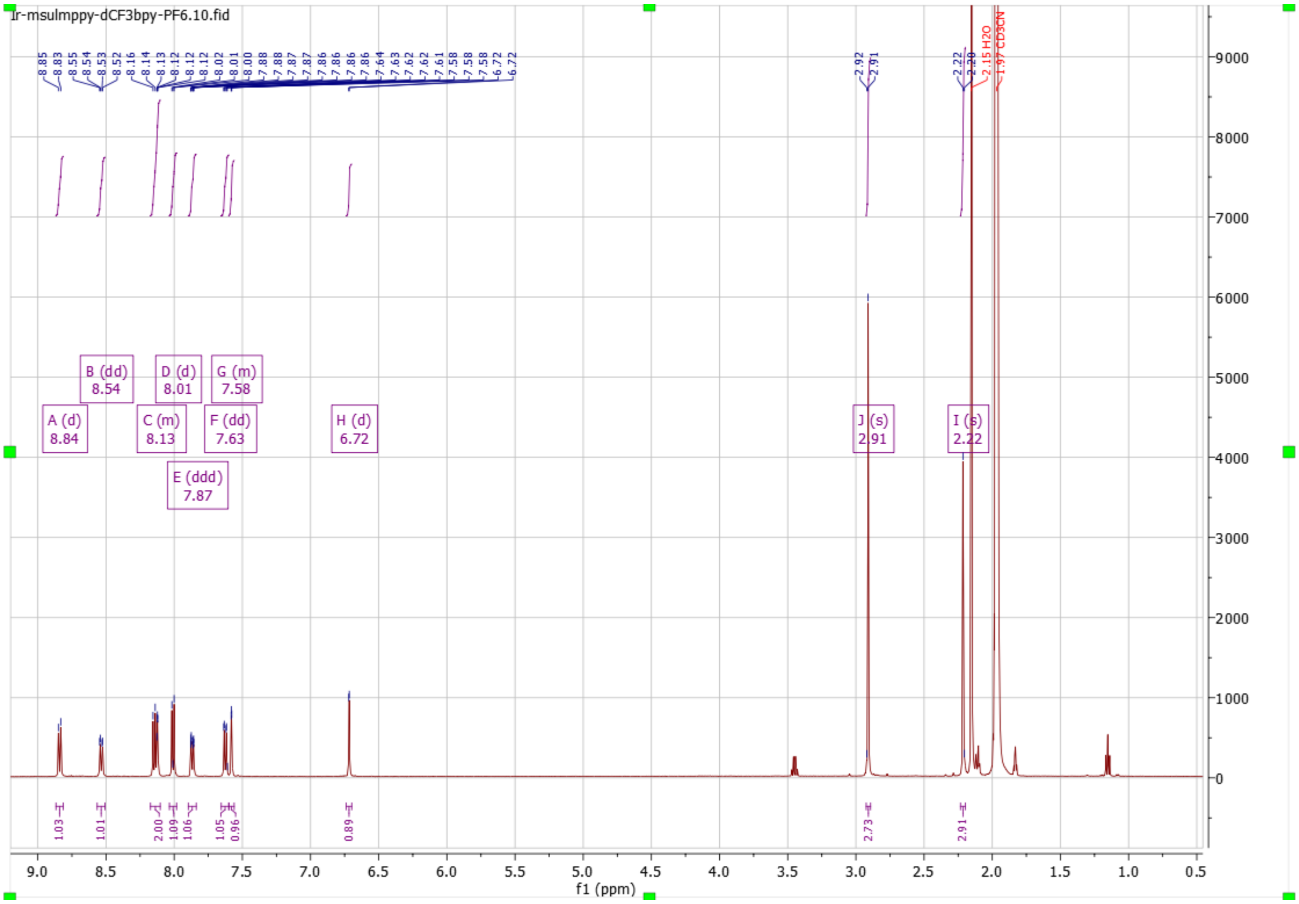

^{1}H NMR (500 MHz, Acetonitrile-d_3) δ 8.84 (d, *J* = 8.6 Hz, 2H), 8.54 (dd, *J* = 8.7, 2.2 Hz, 2H), 8.17 – 8.10 (m, 4H), 8.01 (d, *J* = 8.2 Hz, 2H), 7.87 (ddd, *J* = 8.2, 2.0, 0.9 Hz, 2H), 7.63 (dd, *J* = 8.2, 1.9 Hz, 2H), 7.60 – 7.56 (m, 2H), 6.72 (d, *J* = 1.9 Hz, 2H), 2.91 (s, 6H), 2.22 (s, 6H).

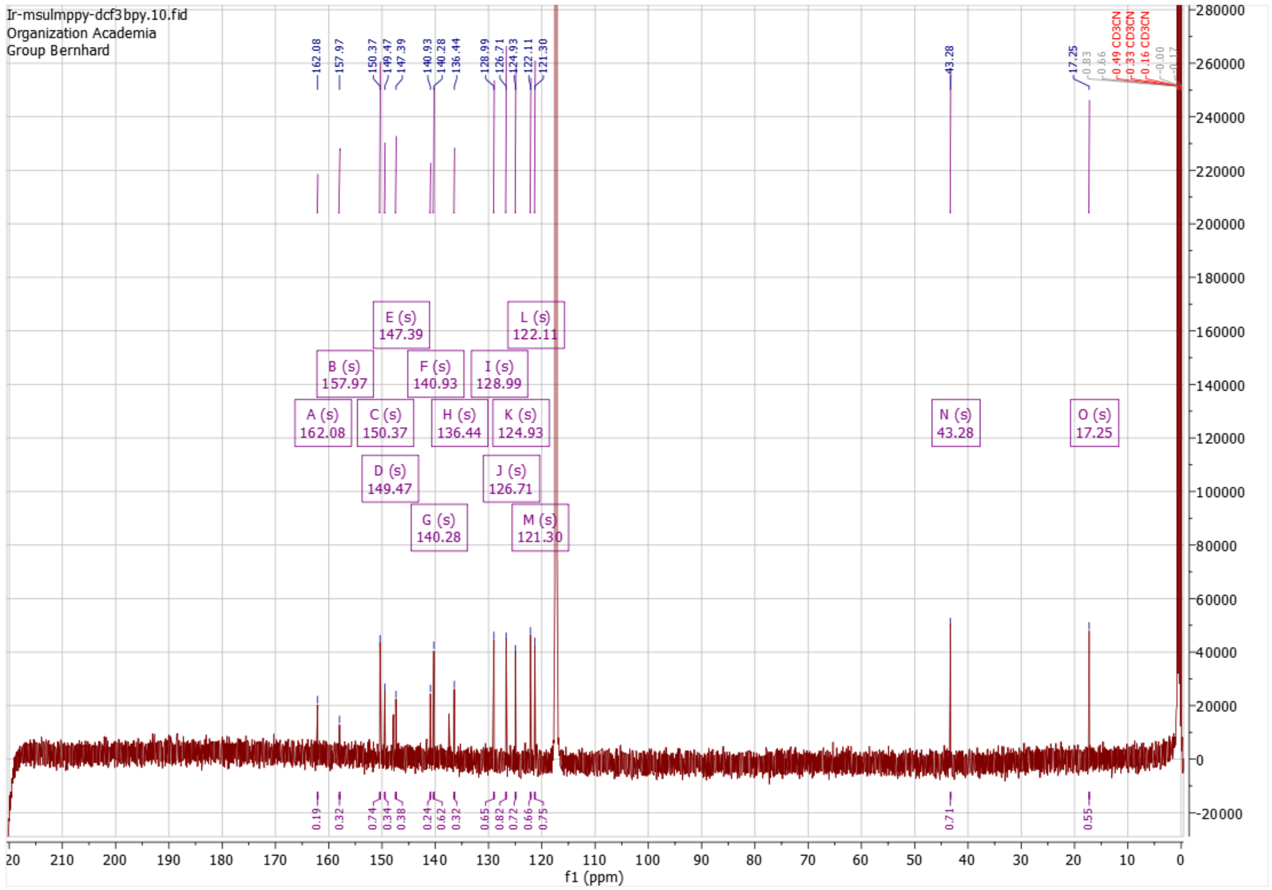

^{13}C NMR (126 MHz, Acetonitrile-d_3) δ 162.08, 157.97, 150.37, 149.47, 147.39, 140.93, 140.28, 136.44, 128.99, 126.71, 124.93, 122.11, 121.30, 43.28, 17.25.

Rh3: – expected mass 765.498 – observed mass 765.0914

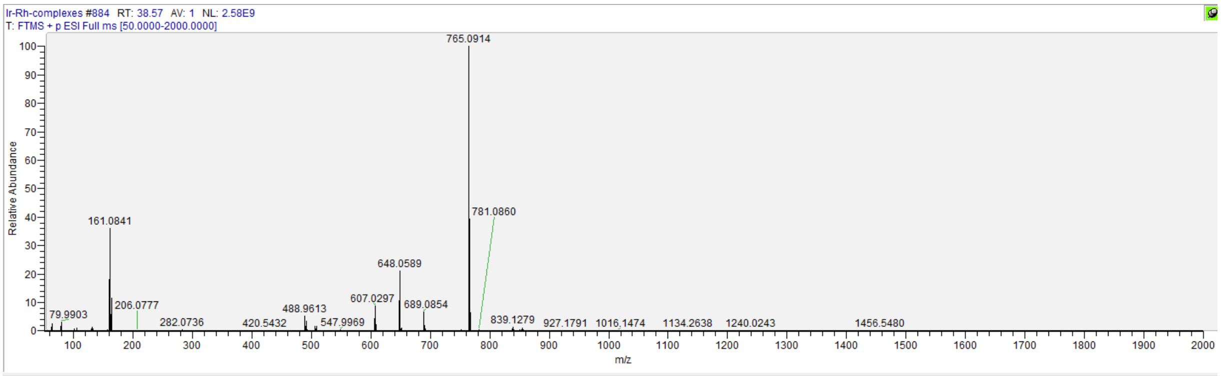

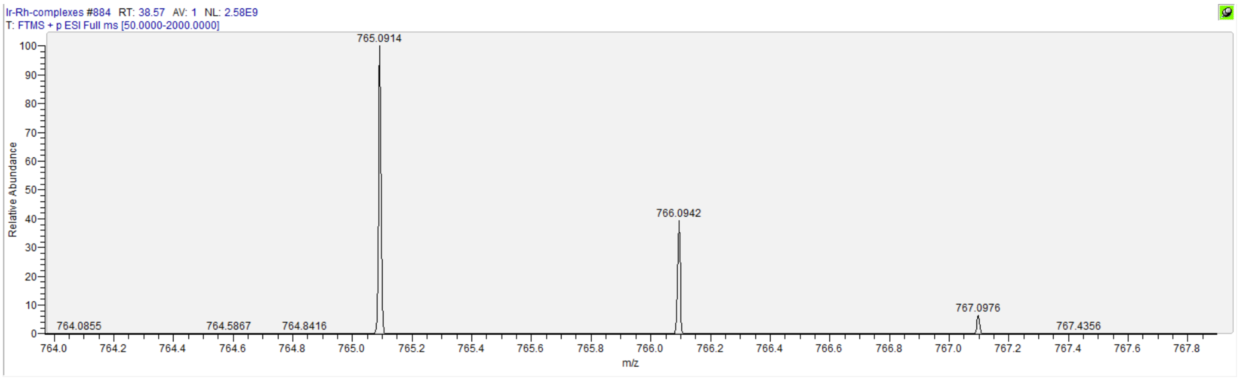

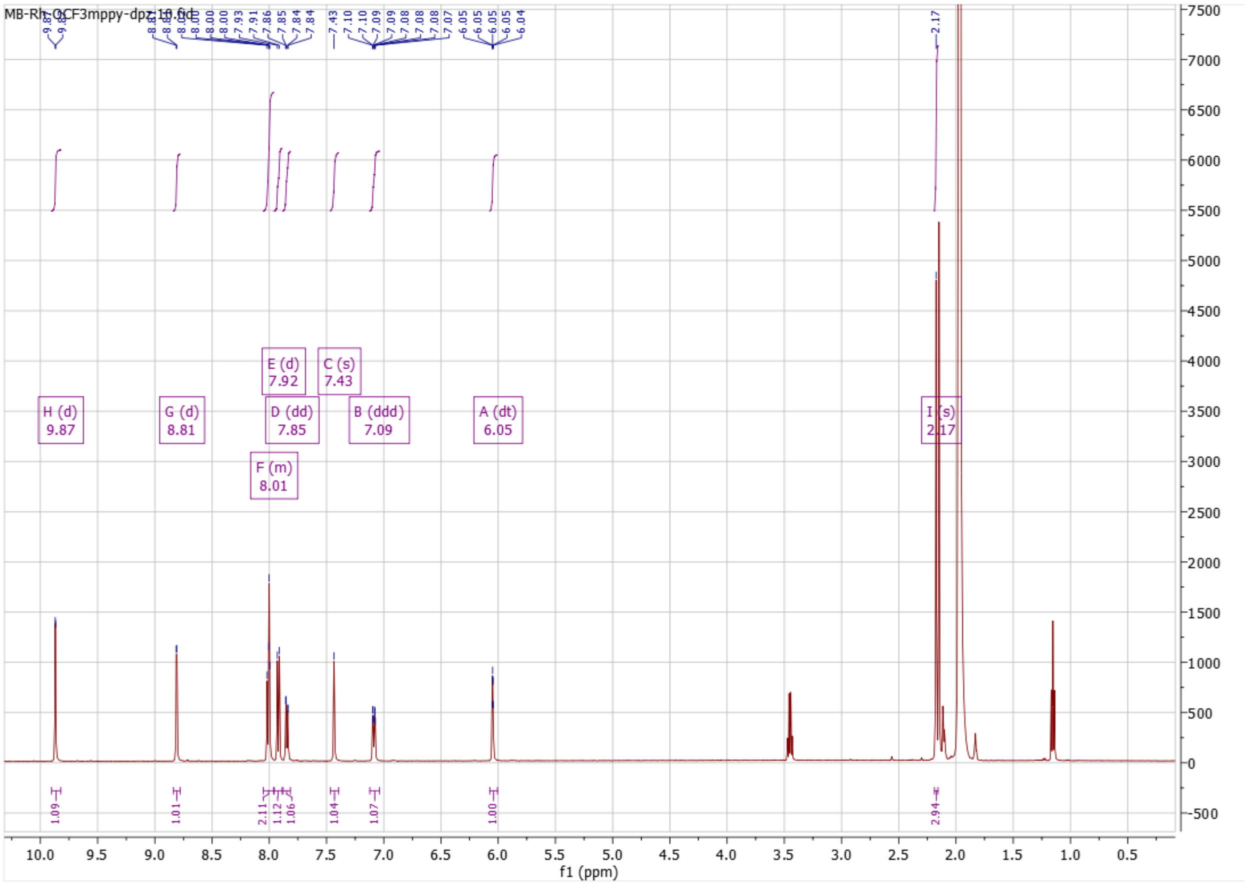

^{1}H NMR (500 MHz, Acetonitrile-d_3) δ 9.87 (d, *J* = 1.4 Hz, 2H), 8.81 (d, *J* = 2.8 Hz, 2H), 8.01 (d, *J* = 8.4 Hz, 4H), 7.92 (d, *J* = 8.6 Hz, 2H), 7.85 (dd, *J* = 8.2, 2.0 Hz, 2H), 7.43 (s, 2H), 7.11 – 7.05 (m, 2H), 6.07 – 6.03 (m, 2H), 2.17 (s, 6H).

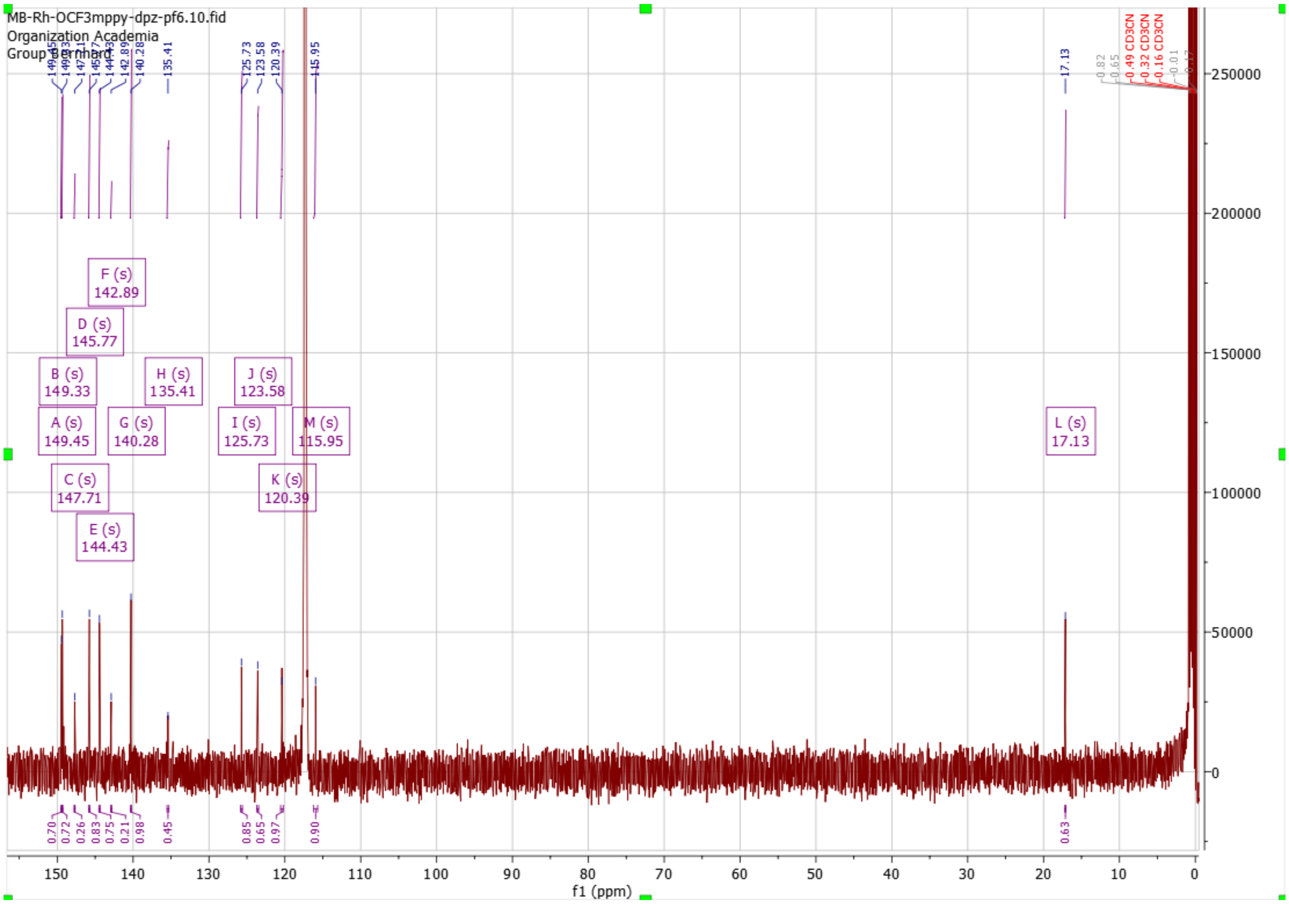

^{13}C NMR (126 MHz, Acetonitrile-*d*$_3$) δ 149.45, 149.33, 147.71, 145.77, 144.43, 142.89, 140.28, 135.41, 125.73, 123.58, 120.39, 115.95, 17.13.

Ir3: – expected mass 854.809 – observed mass 855.1491

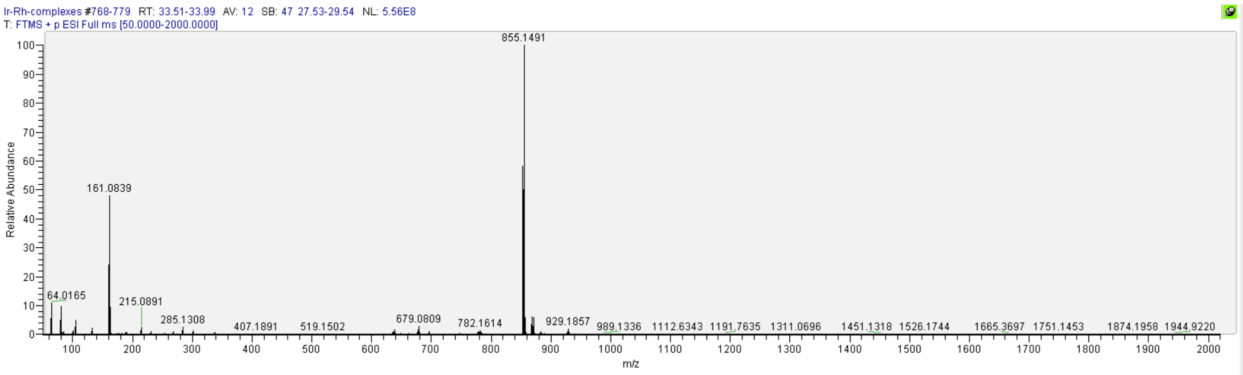

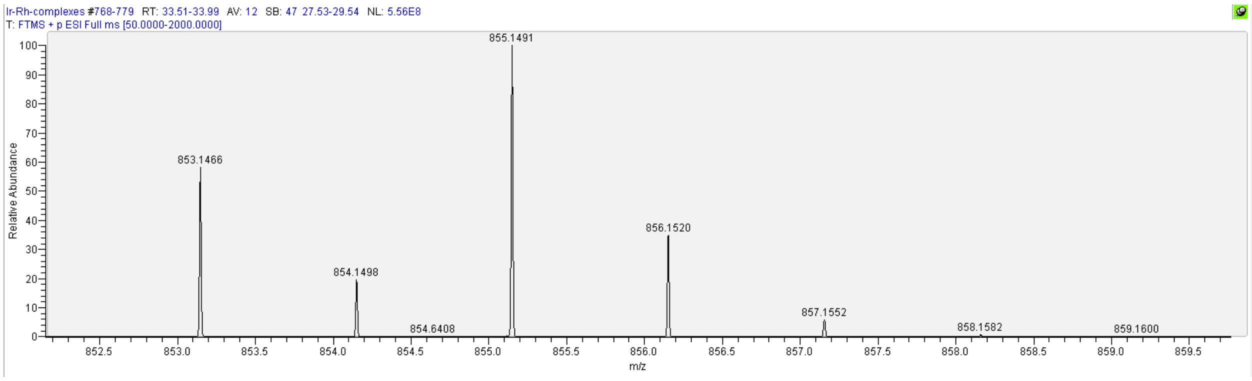

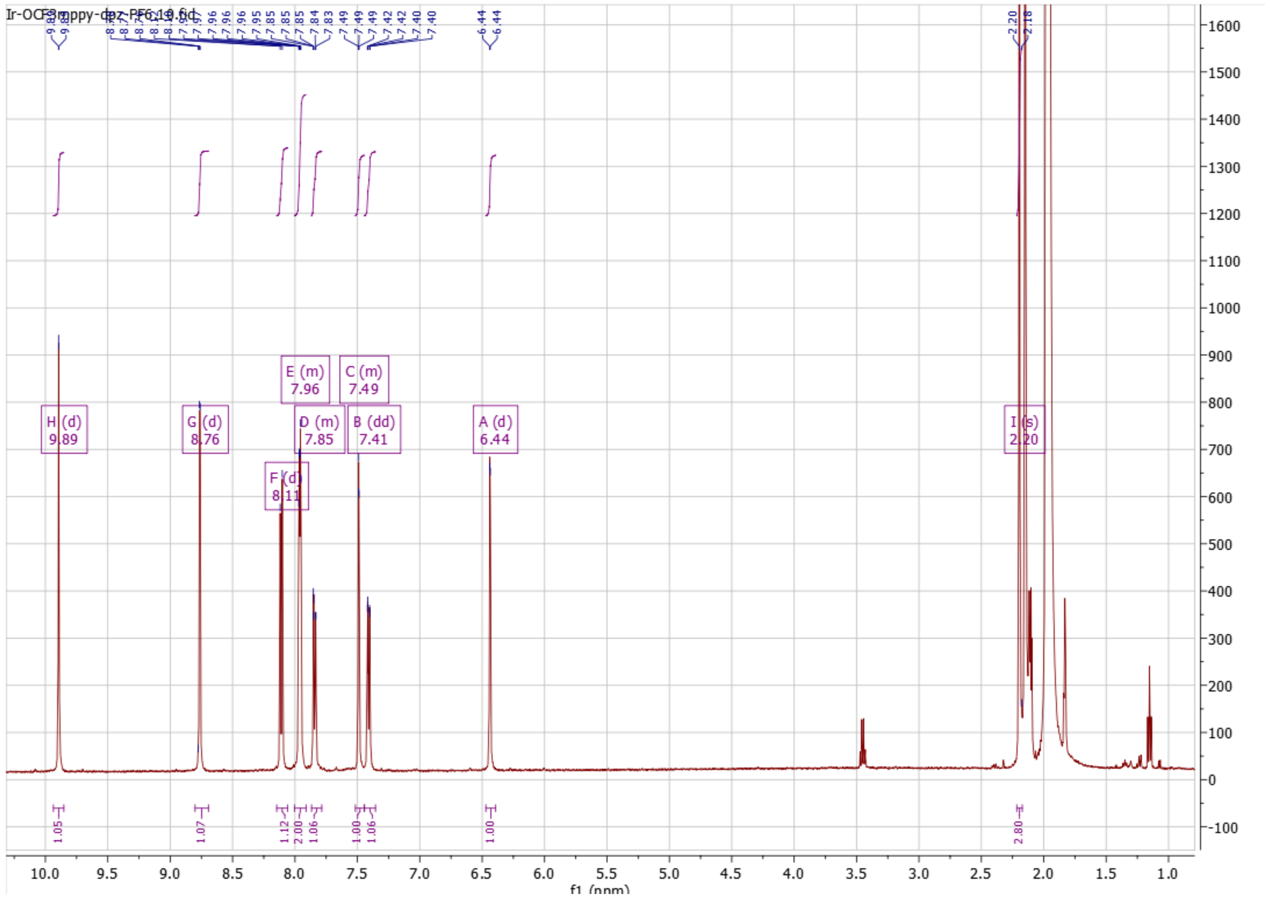

H NMR (500 MHz, Acetonitrile-d_3) δ 9.89 (d, *J* = 1.4 Hz, 2H), 8.76 (d, *J* = 3.0 Hz, 2H), 8.11 (d, *J* = 8.3 Hz, 2H), 8.00 – 7.91 (m, 4H), 7.87 – 7.79 (m, 2H), 7.52 – 7.44 (m, 2H), 7.41 (dd, *J* = 8.3, 1.9 Hz, 2H), 6.44 (d, *J* = 1.9 Hz, 2H), 2.20 (s, 6H).

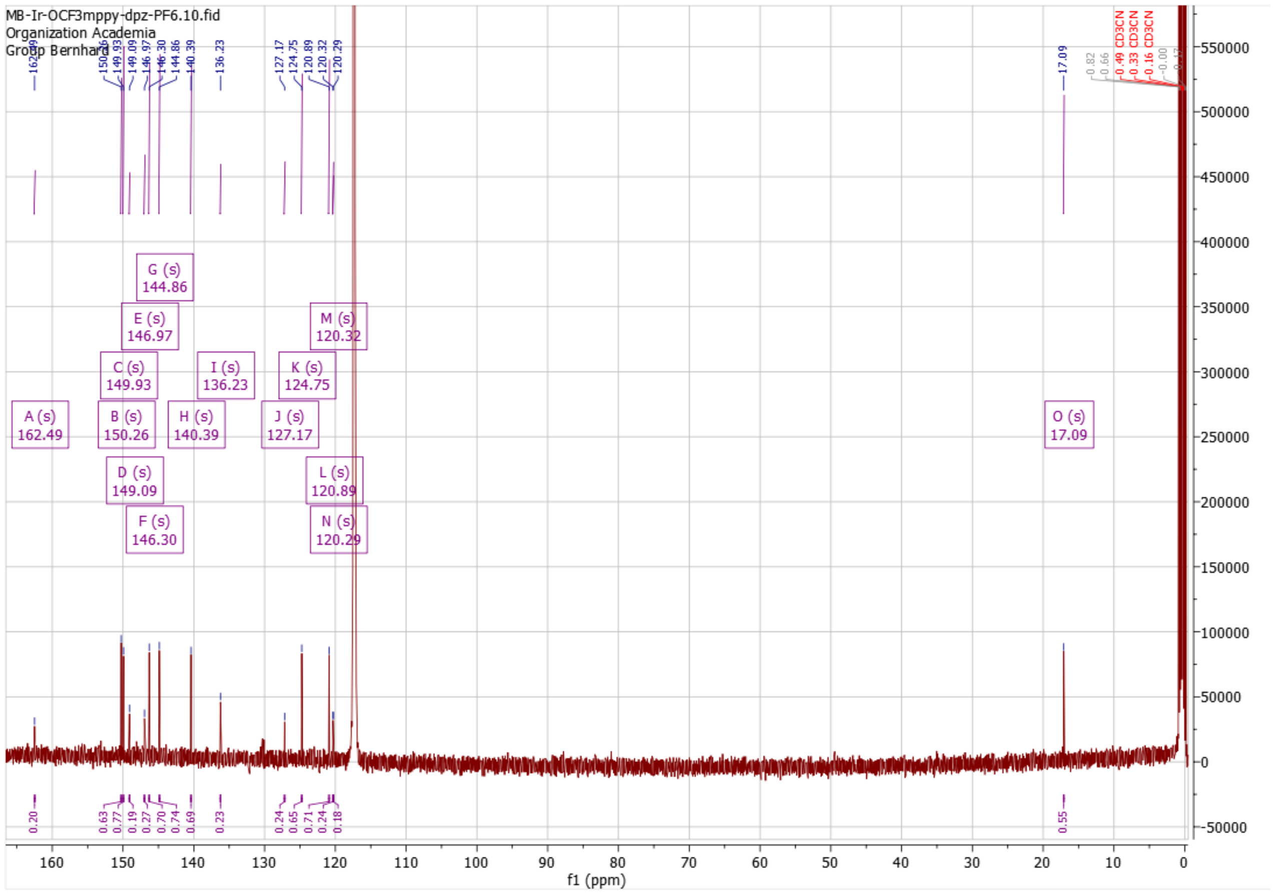

^{13}C NMR (126 MHz, Acetonitrile-d_3) δ 162.49, 150.26, 149.93, 149.09, 146.97, 146.30, 144.86, 140.39, 136.23, 127.17, 124.75, 120.89, 120.32, 120.29, 17.09.

Rh4: – expected mass 789.518 – observed mass 789.0916

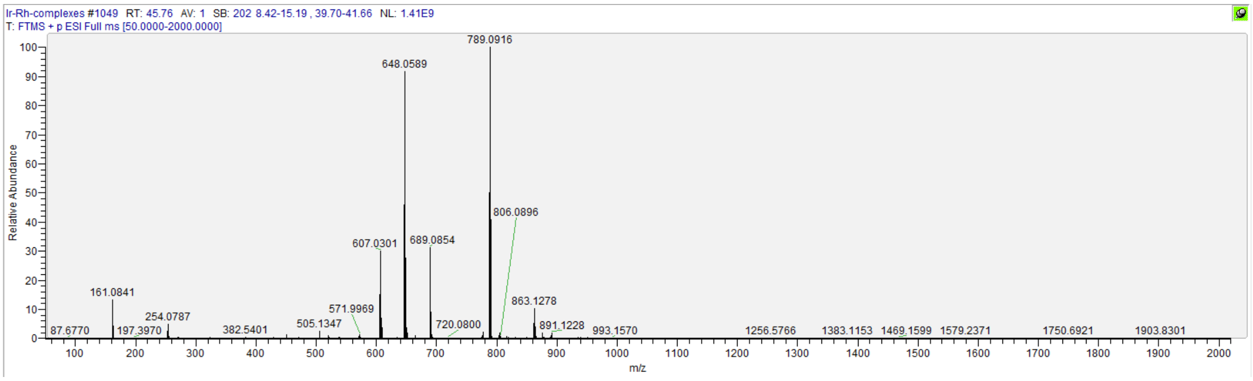

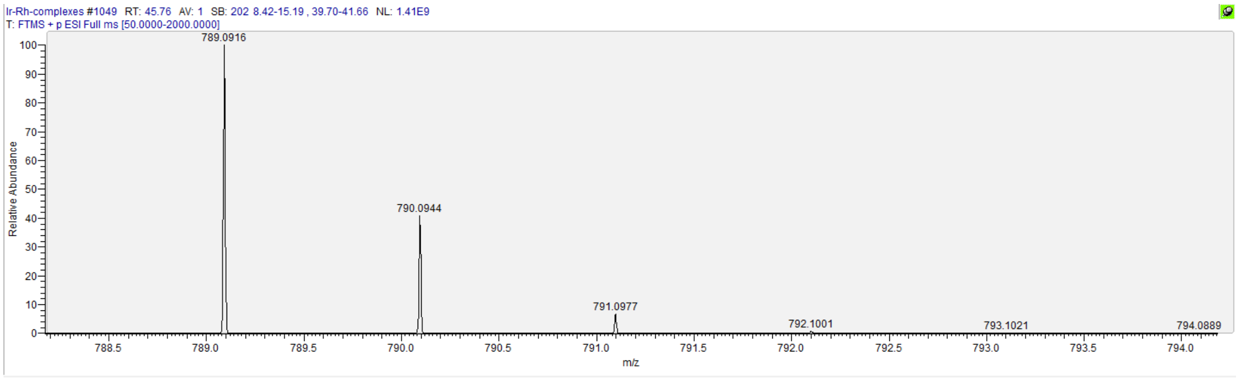

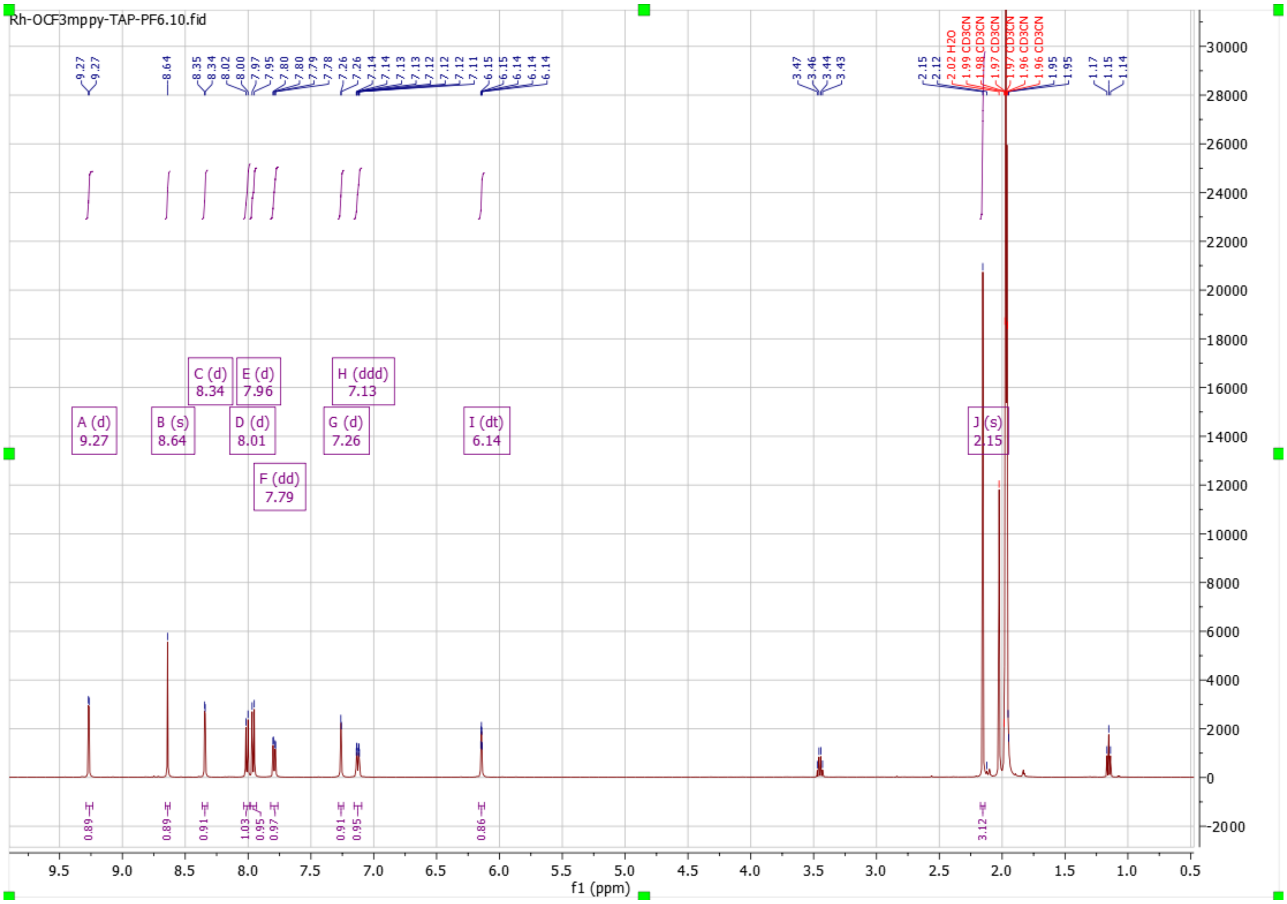

^{1}H NMR (500 MHz, Acetonitrile-d_3) δ 9.27 (d, *J* = 2.4 Hz, 2H), 8.64 (s, 2H), 8.34 (d, *J* = 2.4 Hz, 2H), 8.01 (d, *J* = 8.3 Hz, 2H), 7.96 (d, *J* = 8.5 Hz, 2H), 7.79 (dd, *J* = 8.4, 2.0 Hz, 2H), 7.26 (d, *J* = 1.7 Hz, 2H), 7.13 (ddd, *J* = 8.5, 2.5, 1.2 Hz, 2H), 6.14 (dt, *J* = 2.4, 1.2 Hz, 2H), 2.15 (s, 6H), .

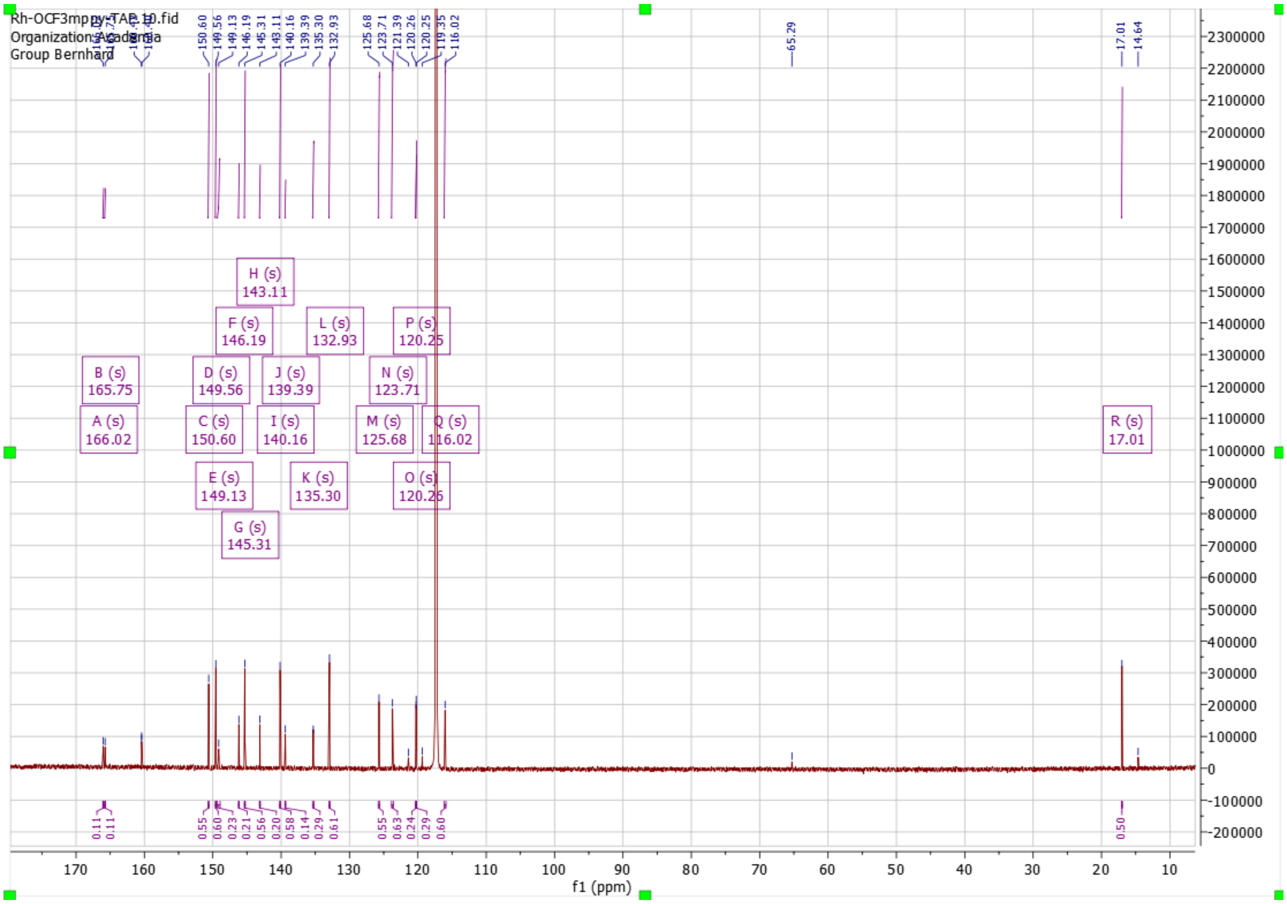

^{13}C NMR (126 MHz, Acetonitrile-d_3) δ 166.02, 165.75, 150.60, 149.56, 149.13, 146.19, 145.31, 143.11, 140.16, 139.39, 135.30, 132.93, 125.68, 123.71, 120.26, 120.25, 116.02, 17.01.

Ir4: – expected mass 878.829 – observed mass 879.1489

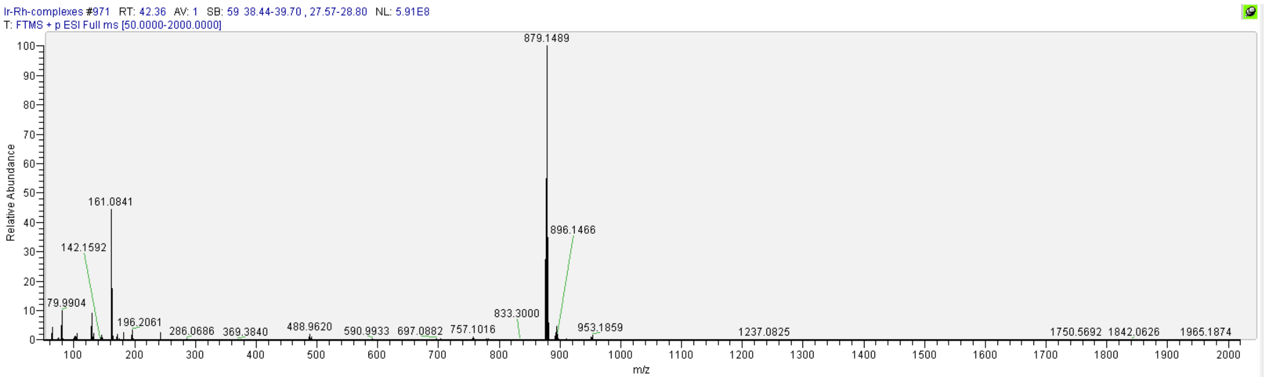

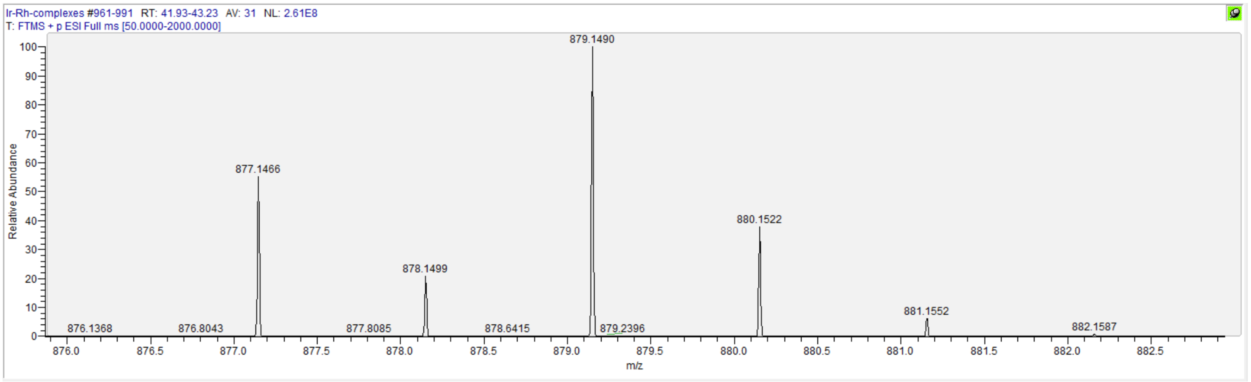

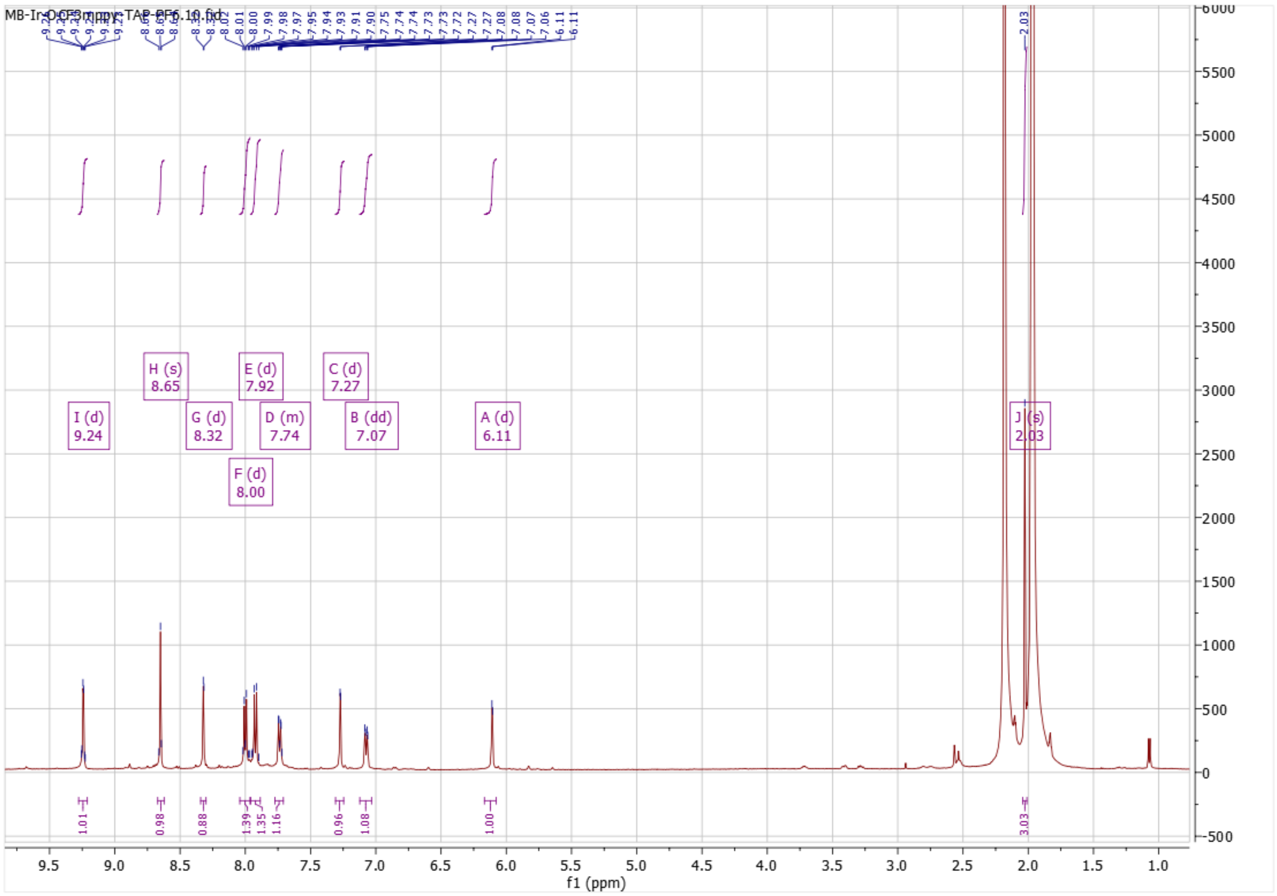

^{1}H NMR (500 MHz, Acetonitrile-d_3) δ 9.24 (d, *J* = 2.6 Hz, 2H), 8.65 (s, 2H), 8.32 (d, *J* = 2.6 Hz, 2H), 8.00 (d, *J* = 8.3 Hz, 2H), 7.92 (d, *J* = 8.5 Hz, 2H), 7.77 – 7.71 (m, 2H), 7.27 (d, *J* = 1.9 Hz, 2H), 7.07 (dd, *J* = 8.5, 2.5 Hz, 2H), 6.11 (d, *J* = 2.4 Hz, 2H), 2.03 (s, 6H).

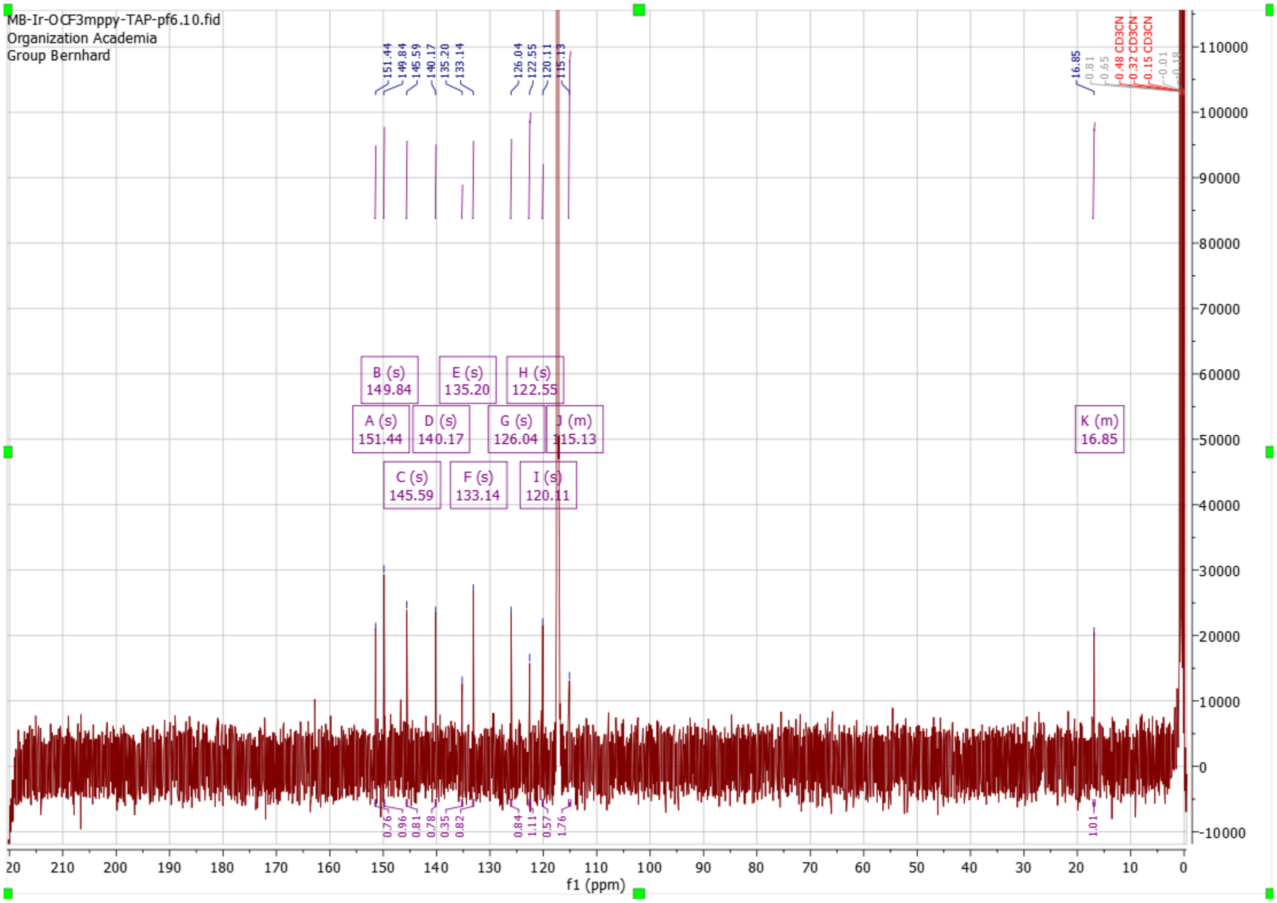

^{13}C NMR (126 MHz, Acetonitrile-d_3) δ 151.44, 149.84, 145.59, 140.17, 135.20, 133.14, 126.04, 122.55, 120.11, 115.28 – 114.89 (m), 17.04 – 16.70 (m).

Rh5: – expected mass 842.676 – observed mass 843.1240

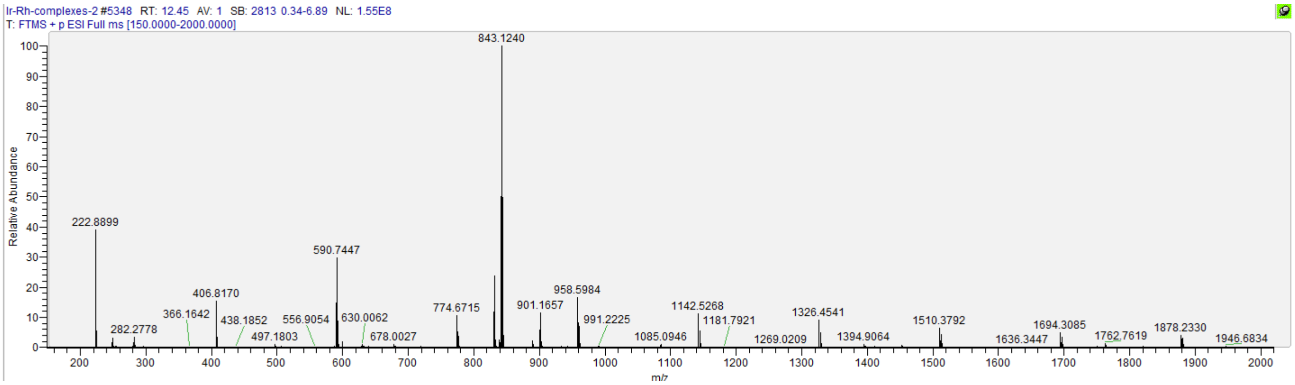

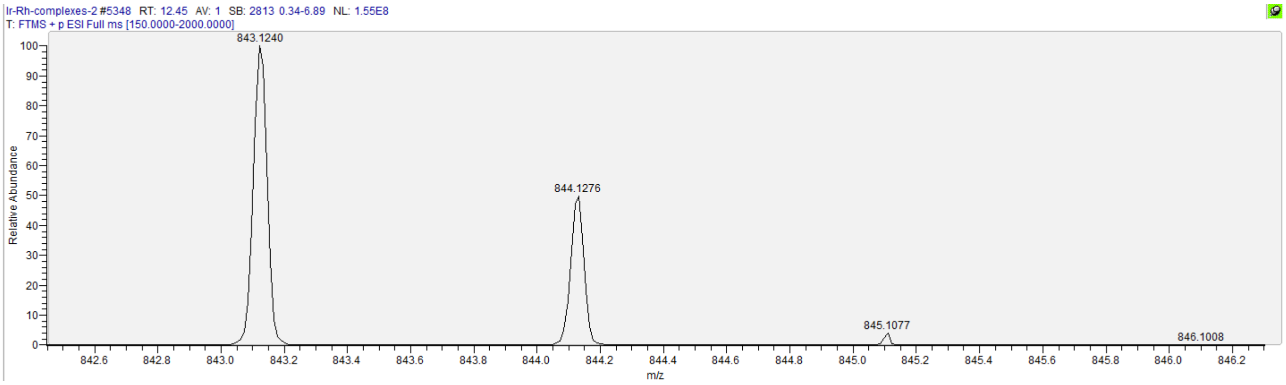

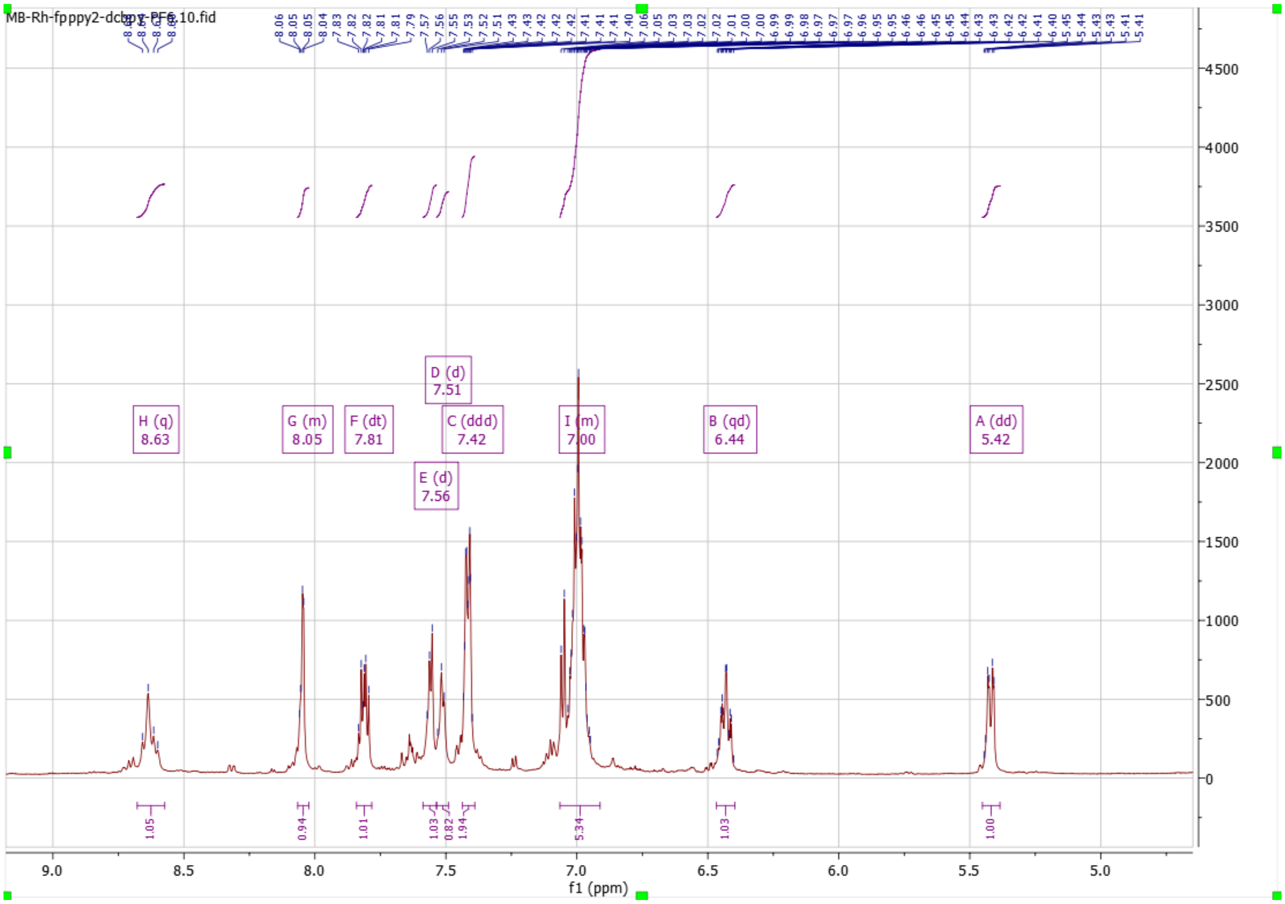

^{1}H NMR (500 MHz, Acetonitrile-d_3) δ 8.63 (q, *J* = 9.5, 8.1 Hz, 2H), 8.07 – 8.02 (m, 2H), 7.81 (dt, *J* = 8.8, 5.3 Hz, 2H), 7.56 (d, *J* = 5.2 Hz, 2H), 7.51 (d, *J* = 5.6 Hz, 2H), 7.42 (ddd, *J* = 7.7, 3.9, 1.8 Hz, 4H), 7.06 – 6.91 (m, 12H), 6.44 (qd, *J* = 8.7, 7.8, 3.5 Hz, 2H), 5.42 (dd, *J* = 9.0, 2.8 Hz, 2H).

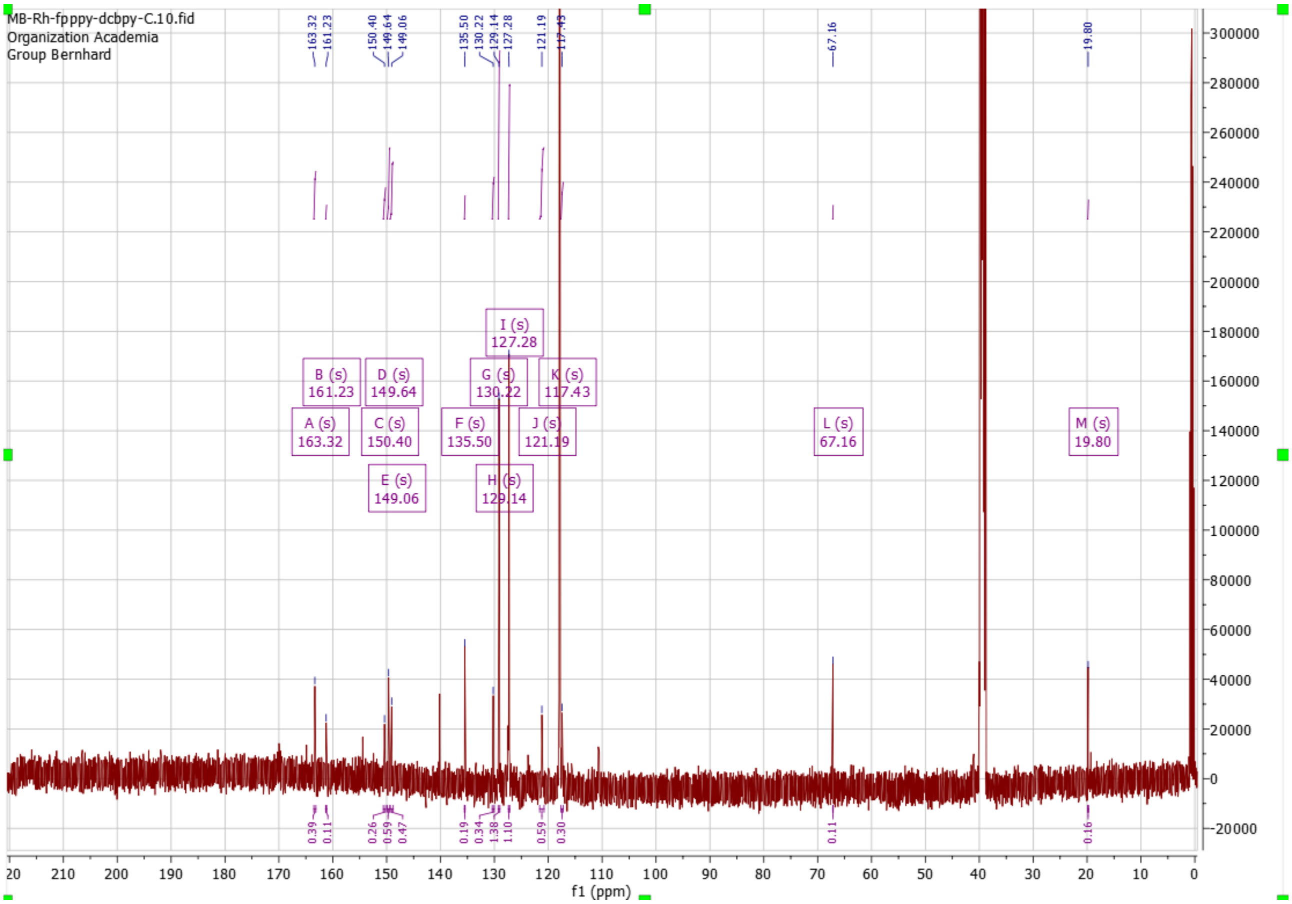

^{13}C NMR (126 MHz, Acetonitrile-d_3) δ 163.32, 161.23, 150.40, 149.64, 149.06, 135.50, 130.22, 129.14, 127.28, 121.19, 117.43, 67.16, 19.80.

Ir5: – expected mass 932.182 – observed mass 933.1830

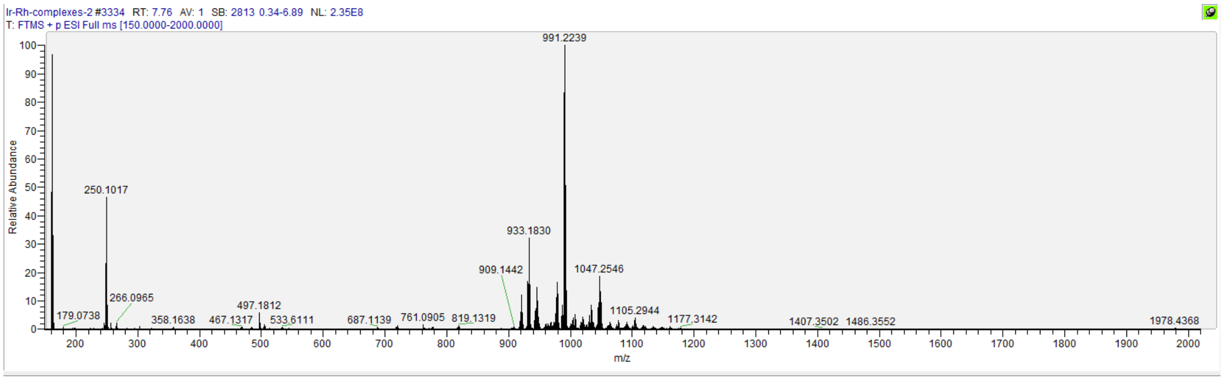

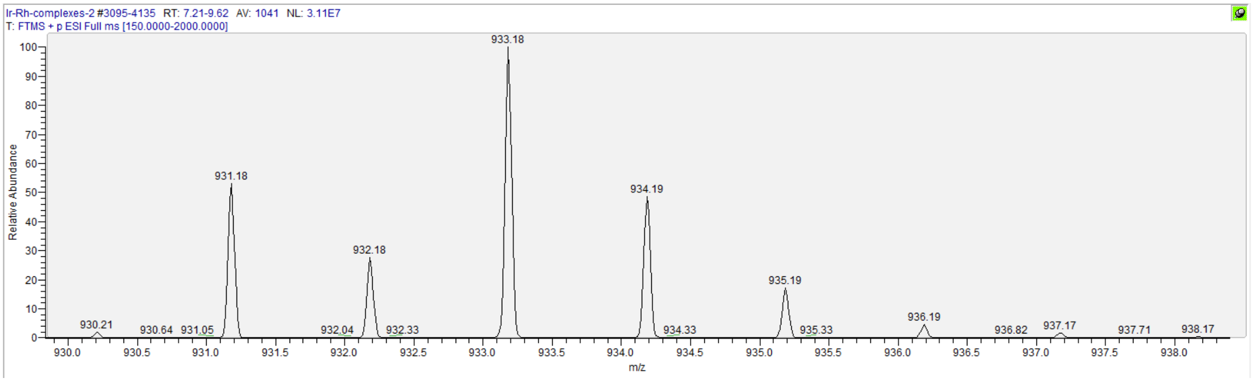

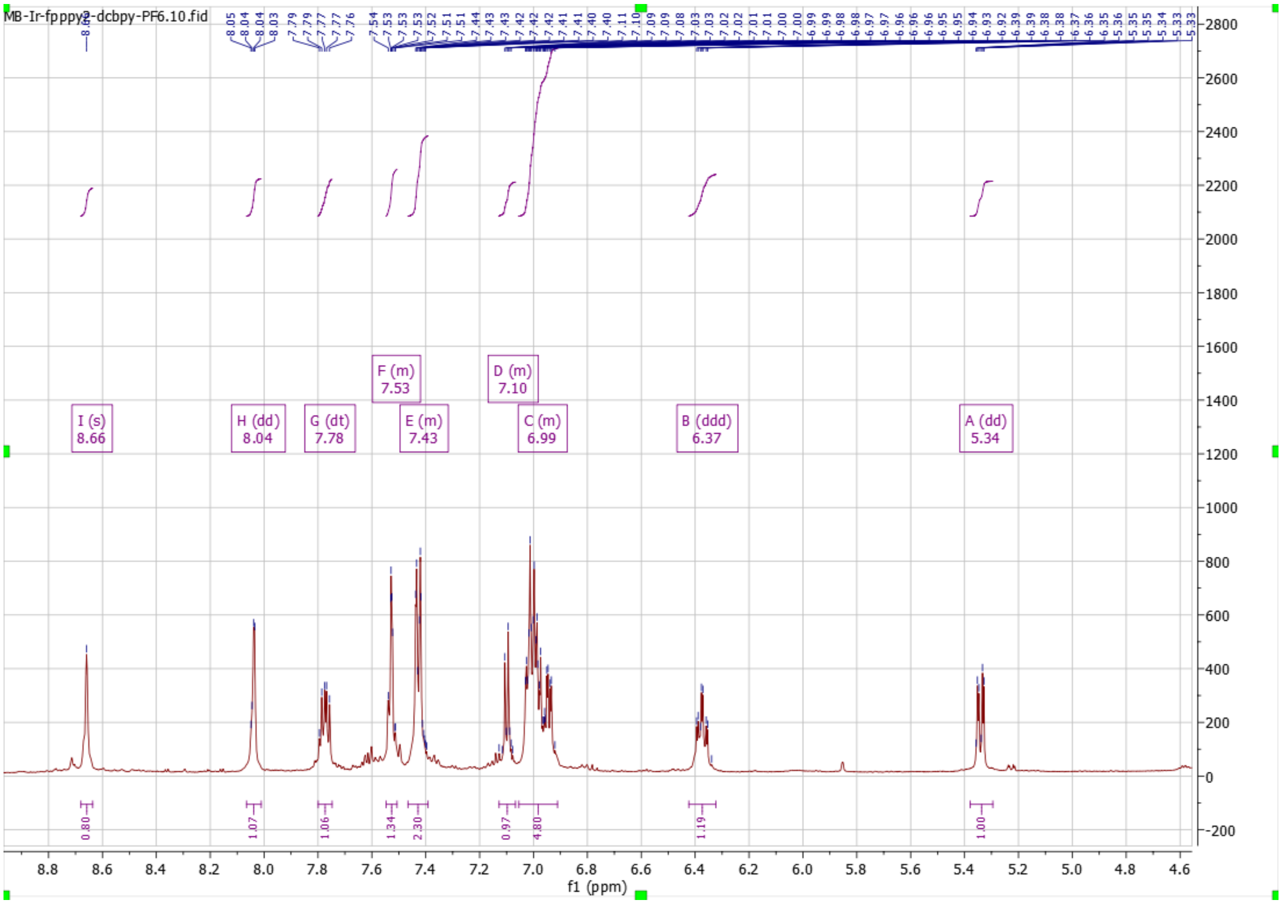

^{1}H NMR (500 MHz, Acetonitrile-d_3) δ 8.66 (s, 2H), 8.04 (dd, J = 4.9, 2.1 Hz, 2H), 7.78 (dt, J = 9.0, 5.1 Hz, 2H), 7.55 – 7.51 (m, 2H), 7.47 – 7.39 (m, 4H), 7.13 – 7.07 (m, 2H), 7.05 – 6.91 (m, 12H), 6.37 (ddd, J = 11.5, 8.0, 2.7 Hz, 2H), 5.34 (dd, J = 9.3, 2.6 Hz, 2H).

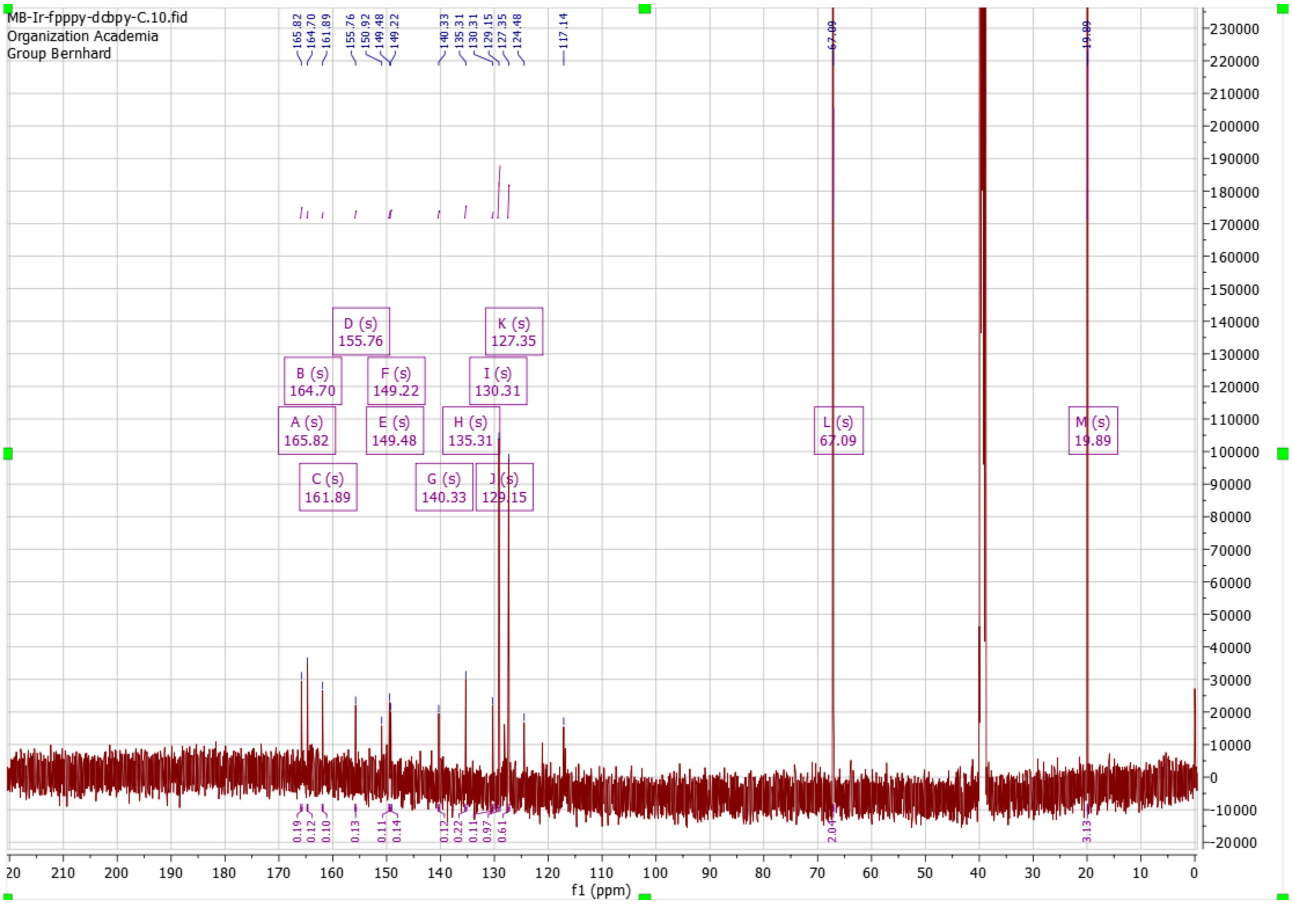

^{13}C NMR (126 MHz, Acetonitrile-d_3) δ 165.82, 164.70, 161.89, 155.76, 149.48, 149.22, 140.33, 135.31, 130.31, 129.15, 127.35, 67.09, 19.89.

Rh6: – expected mass 757.510 – actual mass 757.1017

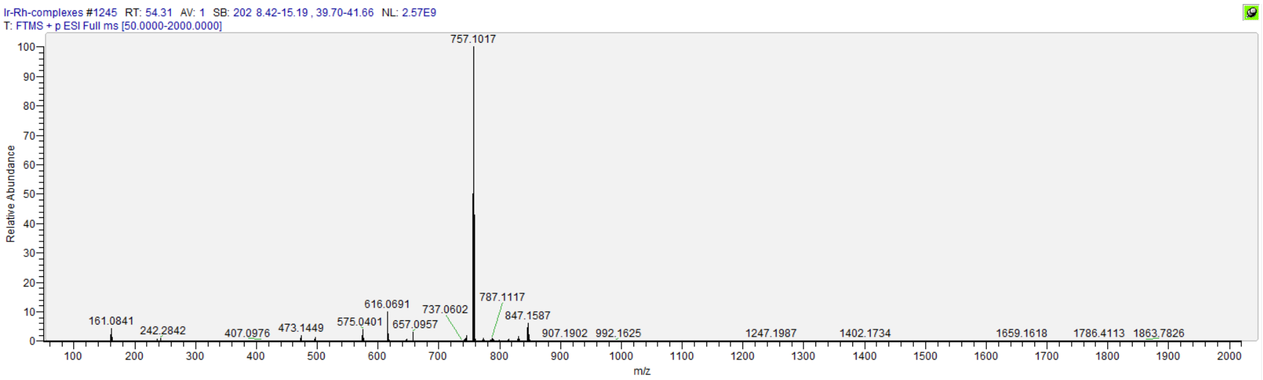

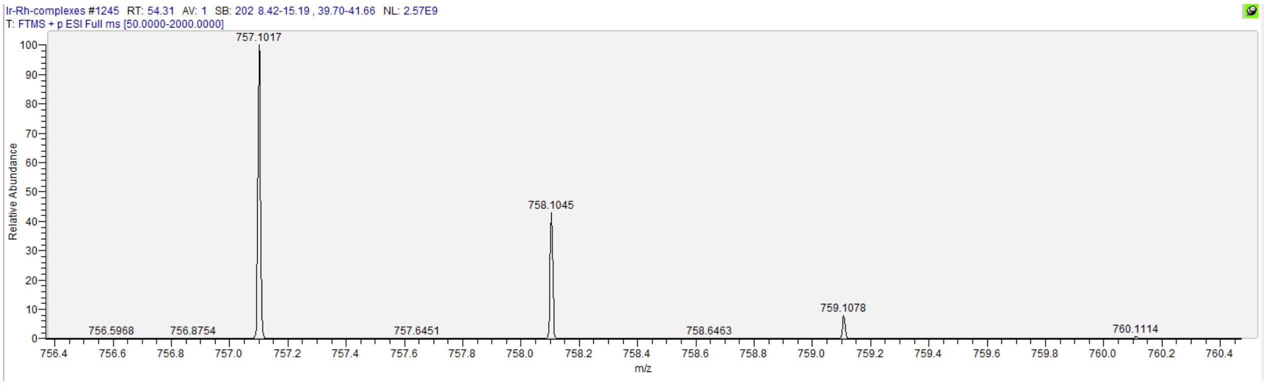

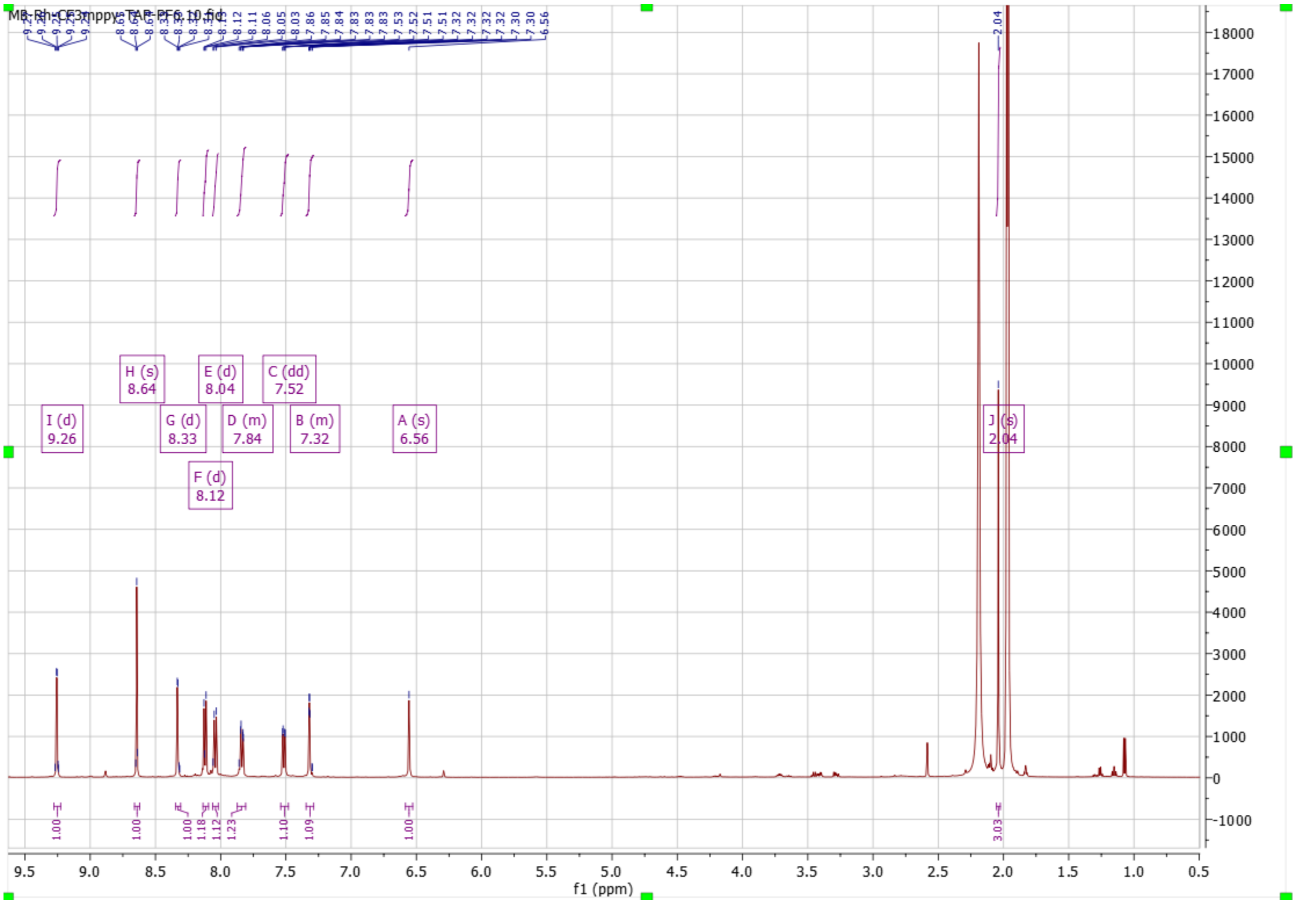

^{1}H NMR (500 MHz, Acetonitrile-d_3) δ 9.26 (d, *J* = 2.4 Hz, 2H), 8.64 (s, 2H), 8.33 (d, *J* = 2.3 Hz, 2H), 8.12 (d, *J* = 8.3 Hz, 2H), 8.04 (d, *J* = 8.1 Hz, 2H), 7.87 – 7.81 (m, 2H), 7.52 (dd, *J* = 8.3, 1.8 Hz, 2H), 7.34 – 7.29 (m, 2H), 6.56 (s, 2H), 2.04 (s, 6H).

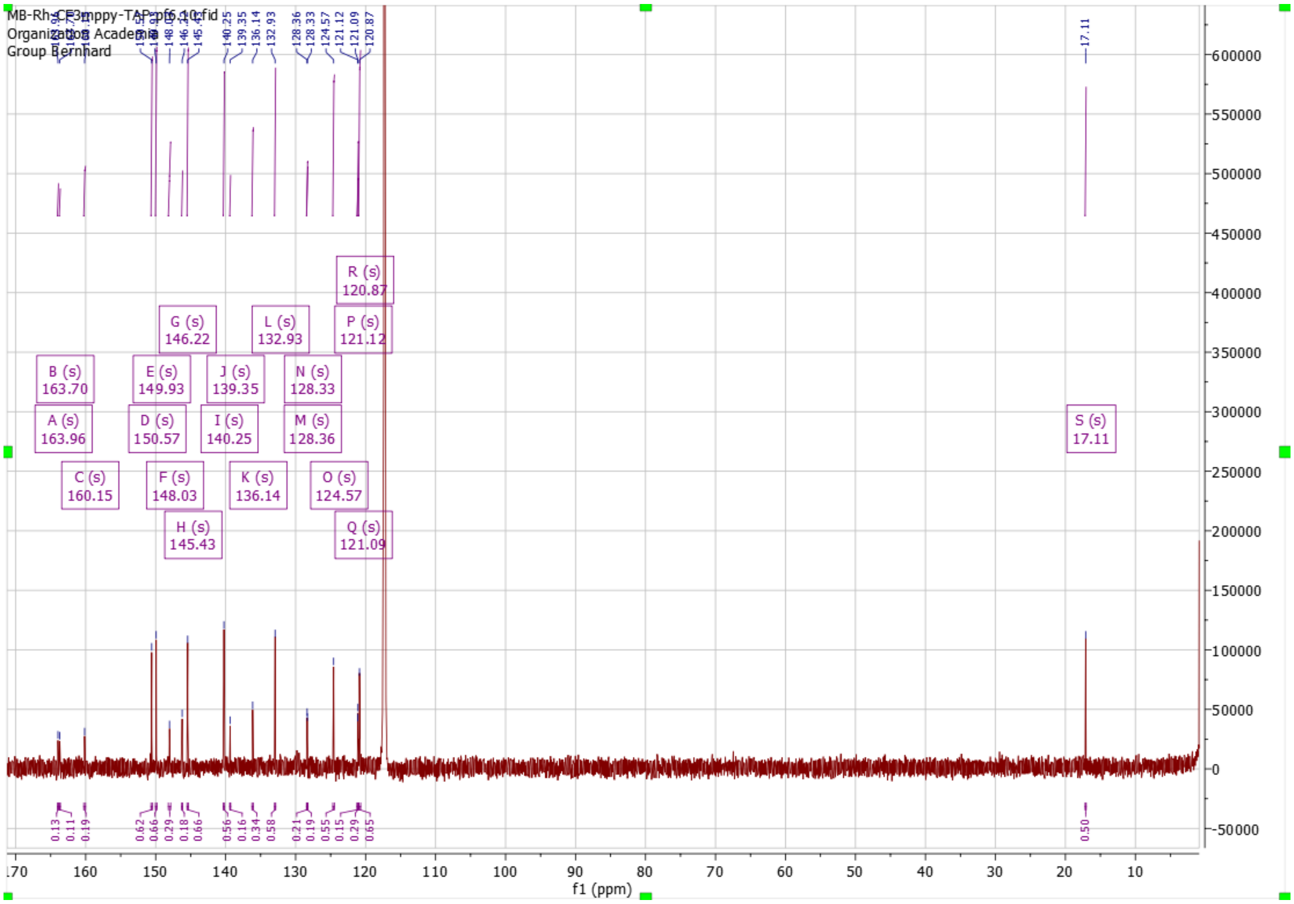

^{13}C NMR (126 MHz, Acetonitrile-d_3) δ 163.96, 163.70, 160.15, 150.57, 149.93, 148.03, 146.22, 145.43, 140.25, 139.35, 136.14, 132.93, 128.36, 128.33, 124.57, 121.12, 121.09, 120.87, 17.11.

Ir6: – expected mass 846.159 – observed mass 847.1586

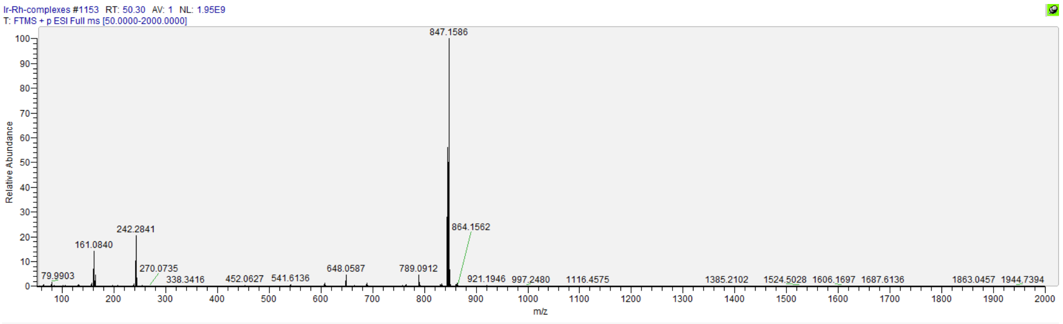

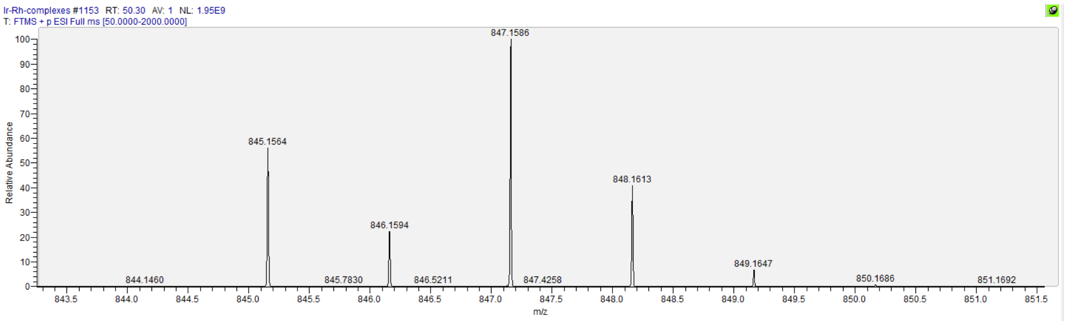

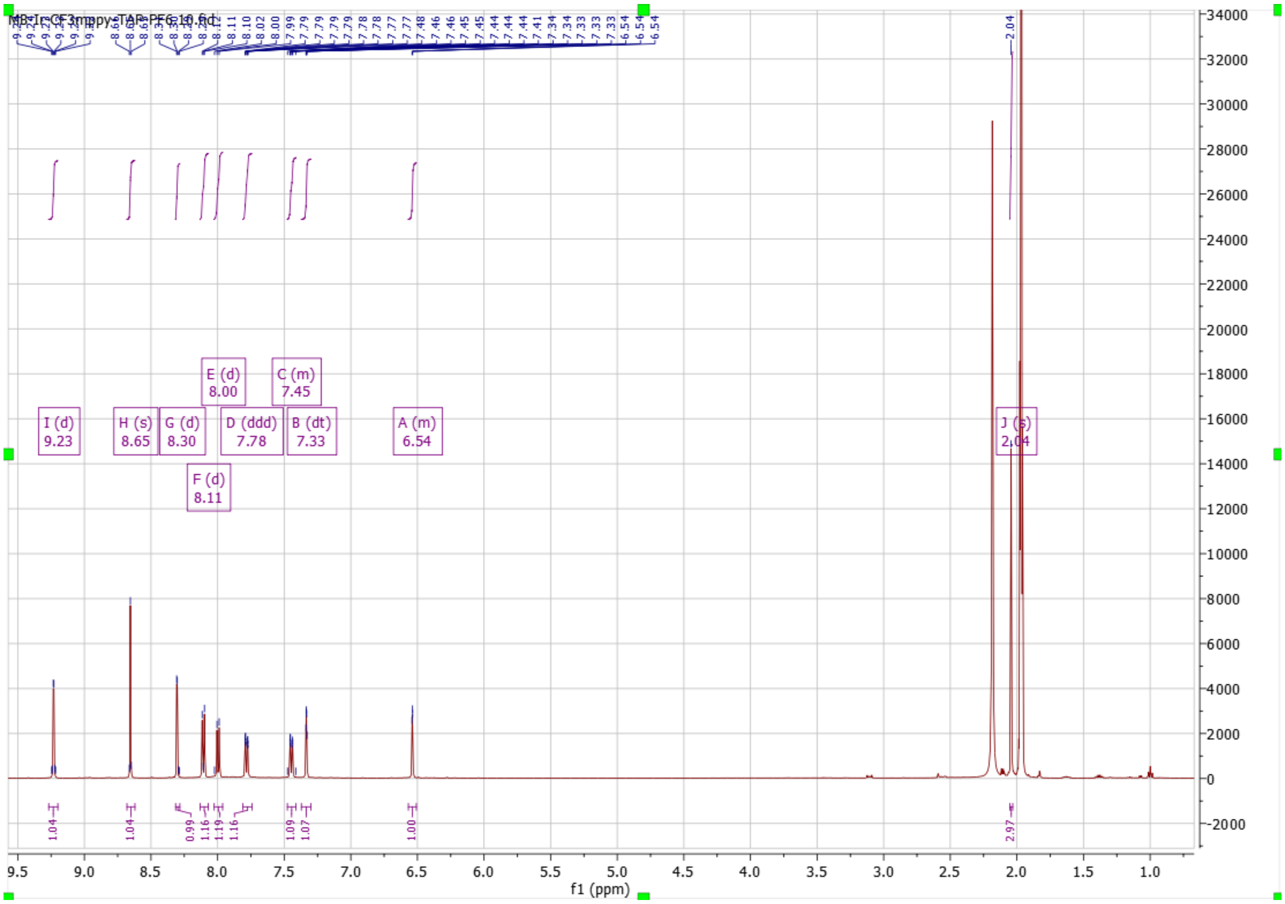

^{1}H NMR (500 MHz, Acetonitrile-d_3) δ 9.23 (d, J = 2.5 Hz, 2H), 8.65 (s, 2H), 8.30 (d, J = 2.5 Hz, 2H), 8.11 (d, J = 8.4 Hz, 2H), 8.00 (d, J = 8.2 Hz, 2H), 7.78 (ddd, J = 8.3, 1.8, 0.8 Hz, 2H), 7.48 – 7.41 (m, 2H), 7.33 (dt, J = 1.8, 0.8 Hz, 2H), 6.57 – 6.51 (m, 2H), 2.04 (s, 6H).

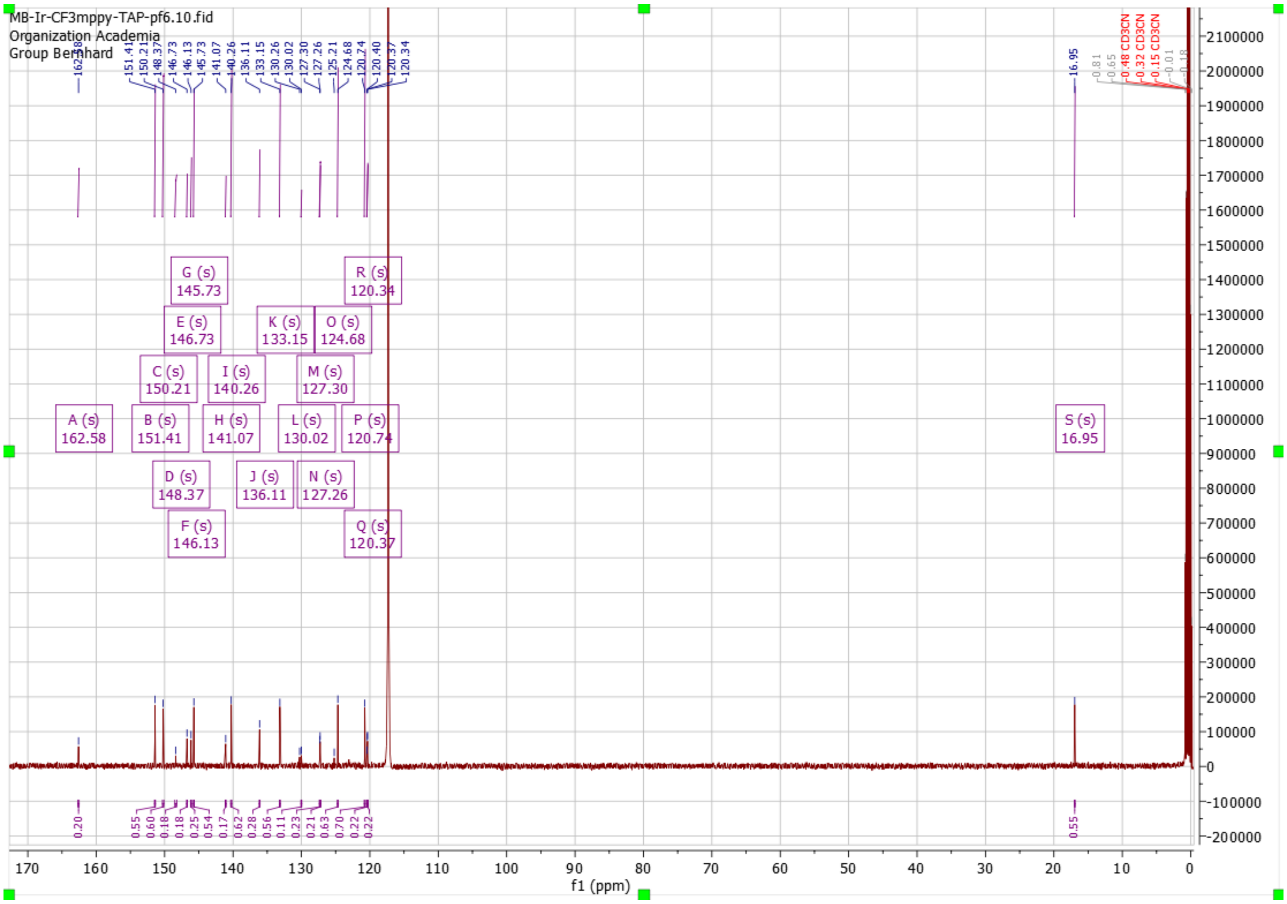

^{13}C NMR (126 MHz, Acetonitrile-d_3) δ 162.58, 151.41, 150.21, 148.37, 146.73, 146.13, 145.73, 141.07, 140.26, 136.11, 133.15, 130.02, 127.30, 127.26, 124.68, 120.74, 120.37, 120.34, 16.95.

References

1. DiLuzio, S.; Connell, T. U.; Mdluli, V.; Kowalewski, J.; Bernhard, S. Understanding Ir(III) Photocatalyst Structure-Activity Relationships: A Highly Parallelized Study of Light-Driven Metal Reduction Processes. *J. Am. Chem. Soc.* **2022**, *144* (3), 1431-1444.
